# A cell-free browning strategy: Exosomal miR-21a-5p from ADSCs targets PDCD4 to reshape adipose metabolism

**DOI:** 10.1016/j.isci.2026.116765

**Published:** 2026-07-14

**Authors:** Chengyao Xiao, Yue Hu, Tingting Wang, Bowen Yang, Yishu Wang, Hanqi Shui, Yunfei Li, Gaofeng Liang, Jingxia Du

**Affiliations:** 1 College of Basic Medicine and Forensic Medicine, Henan University of Science and Technology, Luoyang 471023, China

**Keywords:** adipose mesenchymal stem cells, exosome, miR-21a-5p, adipose tissue browning, PDCD4

## Abstract

Adipose-derived stem cell exosomes (ADSC-EXOs) serve as cell-free therapy, transporting signaling molecules that modulate adipose plasticity. However, the precise molecular mechanisms involved remain unclear. We used a high-fat diet-induced mouse obesity model and an MDI-induced 3T3-L1 cell differentiation model. We found that ADSC-EXOs improved systemic glucose and lipid metabolism, decreased PDCD4 and white adipocyte marker expression, and increased brown adipocyte marker expression along with the activation of the LXR-α/Akt pathway in inguinal adipose tissue. miR-21a-5p mimic or siPDCD4 transfection in 3T3-L1 cells recapitulated these effects, reducing lipid accumulation and promoting adipocyte browning, whereas PDCD4 overexpression produced the opposite effects. Mechanistically, miR-21a-5p directly targeted the 3′ UTR of PDCD4. These findings indicate that ADSC-EXOs facilitate white fat browning through the miR-21a-5p/PDCD4 axis and thus activate the LXR-α/Akt signaling pathway, offering a potential therapeutic strategy for obesity-related metabolic dysfunction.

## Highlights

• ADSC-EXOs improve glucose/lipid metabolism and promote white fat browning
• MiR-21a-5p in ADSC-EXOs targets PDCD4 to activate LXR-α/Akt signaling
• ADSC-EXOs offer a potential therapy for obesity-related metabolic dysfunction

## Graphical abstract

**Figure:**
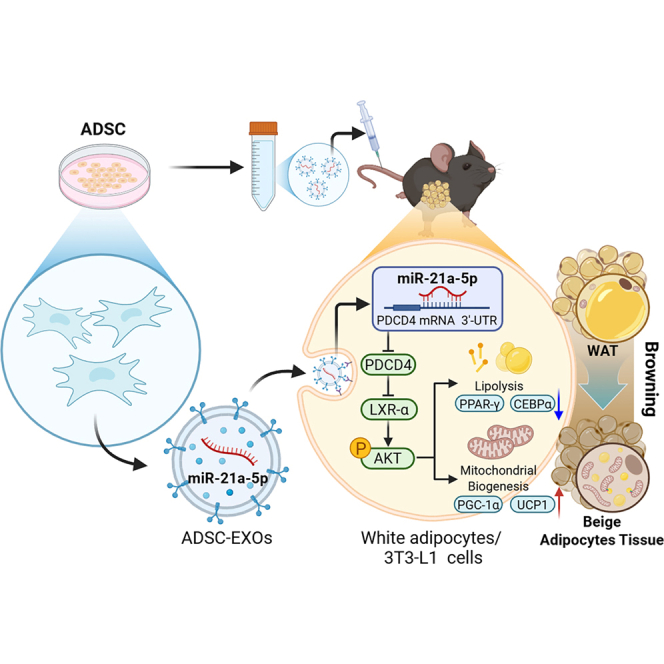

## Teaser

Molecular biology; Cell biology; Stem cells research

## Introduction

Adipose tissue stores energy as triglycerides (TGs) and releases it on demand, while also secreting hormones and cytokines that regulate metabolism, inflammation, and other physiological processes. Excessive accumulation of adipose tissue leads to obesity and metabolic disorders, including metabolic syndrome, type 2 diabetes mellitus, and non-alcoholic fatty liver disease (NAFLD).^1^^,^^2^ The three primary categories of adipose tissue are white (WAT), brown (BAT), and beige/brite.^3^ Beige adipocytes, possessing thermogenic capacity, can be induced within WAT depots and are distinguished by elevated levels of mitochondrial uncoupling protein-1 (UCP-1) expression.^3^^,^^4^ Consequently, regulating adipose plasticity to promote browning represents a potential optimal intervention strategy for obesity and metabolic diseases due to its association with weight loss and enhanced insulin sensitivity.^5^^,^^6^

Adipose-derived mesenchymal stem cells (ADSCs) are a key source of adipocytes, valued for their plentiful availability and straightforward isolation process.^7^ They hold broad application prospects in tissue repair and regeneration,^8^^,^^9^ and play an essential role in maintaining metabolic homeostasis.^10^ ADSC-derived exosomes (ADSC-EXOs) are naturally secreted nanovesicles, measuring 30–150 nm, that transport various signaling molecules such as proteins, lipids, and RNAs, including non-coding RNAs. They regulate target cells via autocrine or paracrine mechanisms.^11^^,^^12^ MicroRNAs (miRNAs) play a crucial role in exosome activities through post-transcriptional gene regulation, primarily by binding to the 3′ UTR of target messenger RNAs.^13^

Accumulating evidence underscores the key roles of miRNAs in adipogenesis and energy homeostasis. miR-193b-365 was initially identified as the miRNA responsible for sustaining brown adipocyte differentiation by suppressing the myogenic potential of preadipocytes.^14^ miR-10a-3p mitigates adipose inflammation through the TGF-β1/Smad3 pathway,^15^ whereas miR-206-3p hinders adipogenesis in 3T3-L1 cells by targeting c-Met and deactivating the PI3K/Akt pathway.^16^ miR-21a-5p is involved in metabolic regulation, with increased hepatic expression observed in animal models of MAFLD/nonalcoholic steatohepatitis (NASH).^17^ Aerobic exercise ameliorates hyperlipidemia by miR-21a-5p-mediated suppression of genes such as FABP7, HMGCR, ACAT1, and OLR1.^18^ Additionally, both intestinal exosome and hepatic overexpression of miR-21a-5p can influence hepatic lipid metabolism and the progression of NAFLD.^19^ MSC exosomes enhance cardio protection by increasing miR-21a-5p in recipient cells, which subsequently downregulates pro-apoptotic genes such as PDCD4 and PTEN.^20^ A process mediated through the miR-21-5p/PDCD4 axis, which underlies their neuroprotective effects in retinal ischemia-reperfusion injury.^21^

PDCD4, first recognized as a gene that increases expression during apoptosis, is widely expressed, with the liver exhibiting the highest levels.^22^ Through its MA-3 domains, it modulates translation and transcription by interacting with the RNA helicase eukaryotic translation initiation factor 4 A (eIF4A),^23^ while well-characterized as a tumor suppressor that inhibits proliferation, invasion, and metastasis, with its absence or reduced expression noted in various primary tumors.^24^^,^^25^^,^^26^^,^^27^ Emerging evidence links PDCD4 to inflammatory and metabolic diseases.^28^^,^^29^^,^^30^ Pdcd4 limits adipose-derived stem cell self-renewal and white-to-beige trans differentiation by repressing AKT signaling and lactate production, and Pdcd4 deficiency enhances stemness, promotes beige adipocyte formation, and protects against obesity.^31^ However, its potential role in promoting white fat browning remains unexplored. Bioinformatic predictions indicate that PDCD4 is potentially a direct target of miR-21a-5p, pending experimental confirmation.

To investigate the role of ADSC-EXOs in promoting white fat browning and to explore the associated molecular mechanisms, this study employed an *in vivo* high-fat diet (HFD)-induced mouse obesity model and an *in vitro* 3T3-L1 adipocyte differentiation model.

## Results

### Isolation and characterization of ADSC-EXOs

**Figure 1:**
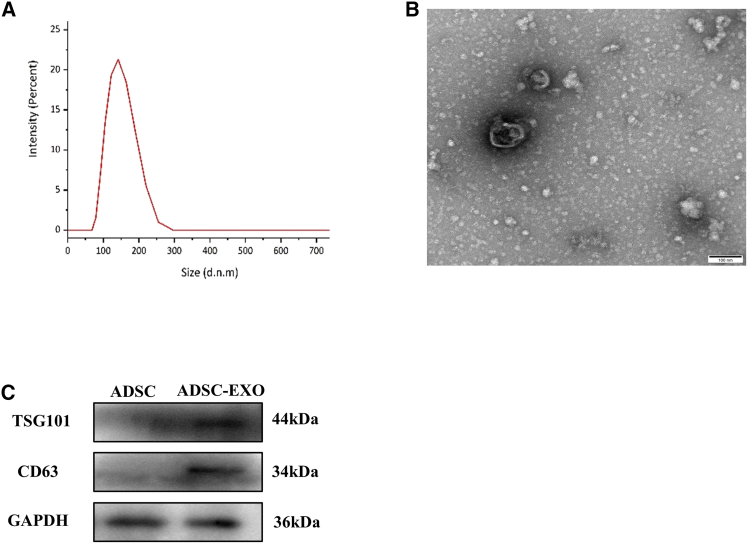
Characterization and extraction of ADSC-EXOs (A) Zetasizer Nano analysis of the diameter distribution of ADSC-EXOs. (B) TEM images illustrating ADSC-EXOs scale bars, 100 μm. (C) Validation of ADSC-EXOs by evaluating exosome protein markers TSG101 and CD36.

ADSC-EXOs were extracted from ADSC-conditioned supernatant via ultracentrifugation and characterized through NTA, TEM, and surface marker identification. NTA indicated that the isolated particles had a diameter distribution of 30–150 nm (Figure 1A). TEM imaging revealed the typical cup-shaped morphology with a bilayer lipid membrane (Figure 1B). Western blot confirmation of high CD63 and TSG101 expression validated the exosome nature of the vesicles (Figure 1C). The data collectively verify the successful isolation of exosomes derived from ADSCs.

### ADSC-EXOs improve glucose and lipid metabolism and promote white adipose tissue browning

**Figure 2:**
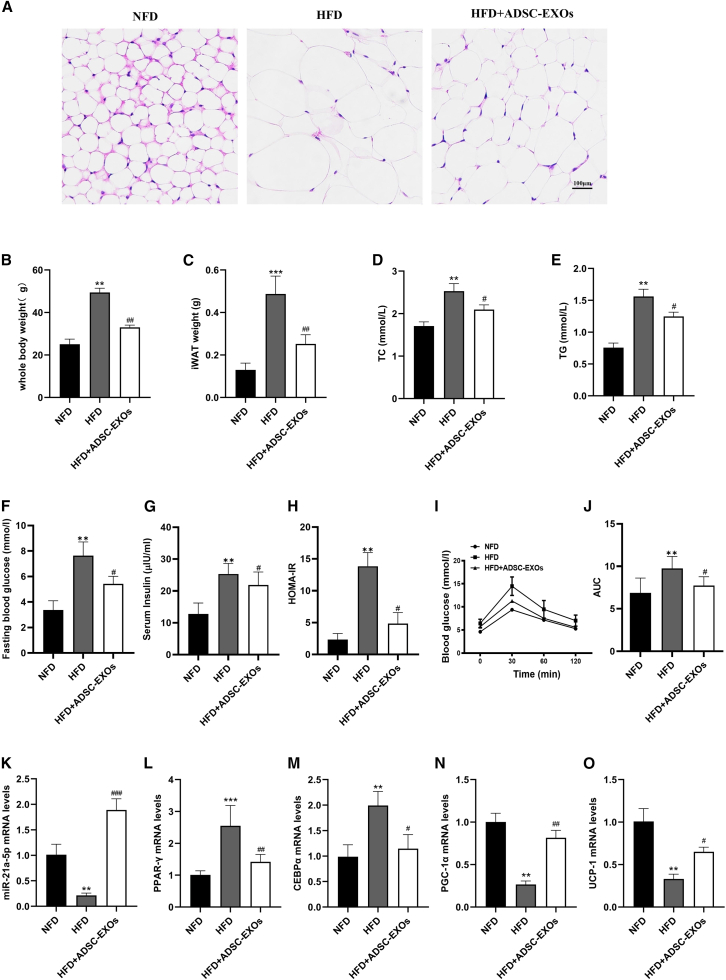
ADSC-EXOs alleviate lipid accumulation and improve glucose metabolism in high-fat diet-fed mice (A) Images representing adipose tissue stained with hematoxylin and eosin (H&E), with scale bars of 100 μm. (B) Body weight of mice. *n* = 6 biological replicates of each. Mean ± SD. ∗∗*p* < 0.01 compared to the NFD group, and ^##^*p* < 0.01 compared to the HFD group by one-way ANOVA with Tukey’s post hoc test. (C) Mouse adipose tissue weight. *n* = 6 biological replicates of each. Mean ± SD. ∗∗∗*p* < 0.001 compared to the NFD group, and ^##^*p* < 0.01 compared to the HFD group by one-way ANOVA with Tukey’s post hoc test. (D) TC content in serum. *n* = 6 biological replicates of each. Mean ± SD. ∗∗*p* < 0.01 compared to the NFD group, and ^#^*p* < 0.05 compared to the HFD group by one-way ANOVA with Tukey’s post hoc test. (E) TG content in serum. *n* = 6 biological replicates of each. Mean ± SD. ∗∗*p* < 0.01 compared to the NFD group, and ^#^*p* < 0.05 compared to the HFD group by one-way ANOVA with Tukey’s post hoc test. (F) Fasting serum glucose. *n* = 6 biological replicates of each. Mean ± SD. ∗∗*p* < 0.01 compared to the NFD group, and ^#^*p* < 0.05 compared to the HFD group by one-way ANOVA with Tukey’s post hoc test. (G) Serum insulin and (H) HOMA-IR. *n* = 6 biological replicates of each. Mean ± SD. ∗∗*p* < 0.01 compared to the NFD group, and ^#^*p* < 0.05 compared to the HFD group by one-way ANOVA with Tukey’s post hoc test. (I) Oral glucose tolerance test (OGTT). *n* = 6 biological replicates of each. (J) Area under the curve (AUC). *n* = 6 biological replicates of each. Mean ± SD. ∗∗*p* < 0.01 compared to the NFD group, and ^#^*p* < 0.05 compared to the HFD group by one-way ANOVA with Tukey’s post hoc test. (K) Quantification of miR-21a-5p expression in adipose tissue was performed using RT-qPCR. *n* = 3 biological replicates of each. Mean ± SD. ∗∗*p* < 0.01 compared to the NFD group, and ^###^*p* < 0.001 compared to the HFD group by one-way ANOVA with Tukey’s post hoc test. (L) Gene expression of white adipokines PPAR-γ and CEBPα (M), and brown adipokines PGC1α (N) and UCP-1 (O). *n* = 4 biological replicates of each. Mean ± SD. ∗∗∗*p* < 0.001 and ∗∗*p* < 0.01 compared to the NFD group, and ^##^*p* < 0.01, ^#^*p* < 0.05 compared to the HFD group by one-way ANOVA with Tukey’s post hoc test. TC, total cholesterol. TG, triglyceride. PPAR-γ, peroxisome proliferator-activated receptor-γ. CEBPα, CCAAT enhancer-binding protein α. UCP-1, mitochondrial uncoupling protein 1. PGC1-α, peroxisome proliferator-activated receptor gamma coactivator 1-alpha.

Male C57BL/6 mice underwent a 16-week HFD regimen, followed by a 4-week ADSC-EXOs treatment administered through tail-vein injection. Hematoxylin and eosin (H&E) staining revealed that a HFD resulted in larger adipocytes in the inguinal adipose tissue (Figure 2A), along with an increase in body weight and inguinal fat mass (Figures 2B and 2C), as well as higher serum total cholesterol (TC) and TG levels (Figures 2D and 2E). In contrast to the normal-fat diet (NFD) group, the HFD group exhibited pronounced glucose metabolism disorders, as evidenced by elevated fasting blood glucose (Figure 2F) and serum insulin levels (Figure 2G), along with aggravated insulin resistance and impaired oral glucose tolerance (Figures 2H–2J). Inguinal adipose tissue from mice on HFD group showed increased expression of white adipogenic markers PPAR-γ and CEBPα (Figures 2L and 2M), while the expression of brown/beige adipogenic markers UCP-1 and PGC-1α (Figures 2N and 2O), along with miR-21a-5p (Figure 2K), was notably reduced.

ADSC-EXOs treatment mitigated HFD-induced metabolic changes, leading to reductions in adipocyte size, body weight, fat mass, and serum TC/TG levels (Figures 2A–2E). Meanwhile, ADSC-EXOs significantly reduced fasting blood glucose (Figure 2F) and serum insulin levels (Figure 2G), ameliorated insulin resistance (Figure 2H), and improved oral glucose tolerance (Figures 2I and 2J). Moreover, ADSC-EXOs downregulated PPAR-γ and CEBPα expression while upregulating UCP-1 and PGC-1α expression, indicating enhanced white-to-beige adipocyte conversion (Figures 2L–2O). Notably, ADSC-EXOs reinstated miR-21a-5p levels in inguinal adipose tissue (Figure 2K). Together, these results demonstrate that ADSC-EXOs improve systemic glucose and lipid metabolism and promote white adipose tissue (WAT) browning in HFD-fed mice.

### ADSC-EXOs downregulate PDCD4 and activate the LXR-α/Akt pathway

**Figure 3:**
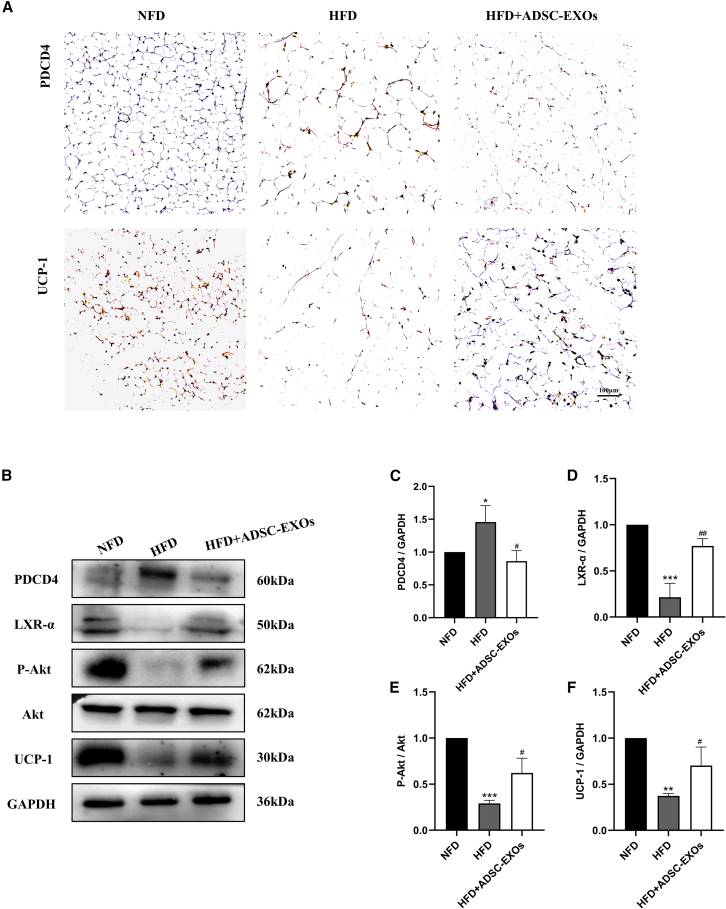
ADSC-EXOs modulate PDCD4 and downstream LXR-α/Akt signaling pathways (A) Representative images of immunohistochemical staining in adipose tissue from 3 independent mice; at least 3 fields per animal were examined (scale bars, 100 μm). (B) Representative western blot images. (C) Quantitative analysis of PDCD4 and LXR-α (D), *p*-Akt/Akt (E), and UCP-1 (F). *n* = 3 biological replicates of each. Mean ± SD. ∗∗∗*p* < 0.001, ∗∗*p* < 0.01, and ∗*p* < 0.05 compared to the NFD group, and ^##^*p* < 0.01, ^#^*p* < 0.05 compared to the HFD group by one-way ANOVA with Tukey’s post hoc test. PDCD4 stands for programmed cell death factor 4. LXR-α, liver X receptor. Akt refers to protein kinase B, while *p*-Akt denotes its phosphorylated form. UCP-1 stands for mitochondrial uncoupling protein 1.

We investigated the role of PDCD4 and the LXR-α/Akt pathway in inguinal adipose tissue to understand the mechanism of ADSC-EXOs-induced white-to-beige adipose tissue conversion. Immunohistochemistry and western blot analyses demonstrated that a HFD notably elevated PDCD4 expression (Figures 3A–3C), while reducing the protein levels of UCP-1 (Figures 3A, 3B, and 3F), LXR-α (Figures 3B and 3D), and phosphorylated Akt (Figures 3B and 3E). ADSC-EXOs treatment reversed these changes, reducing PDCD4 levels while enhancing UCP-1 expression and LXR-α/Akt pathway activation. These findings suggest that ADSC-EXOs promote WAT browning partly through suppressing PDCD4 and subsequently activating the LXR-α/Akt signaling axis.

### PDCD4 expression increases during 3T3-L1 adipogenic differentiation

**Figure 4:**
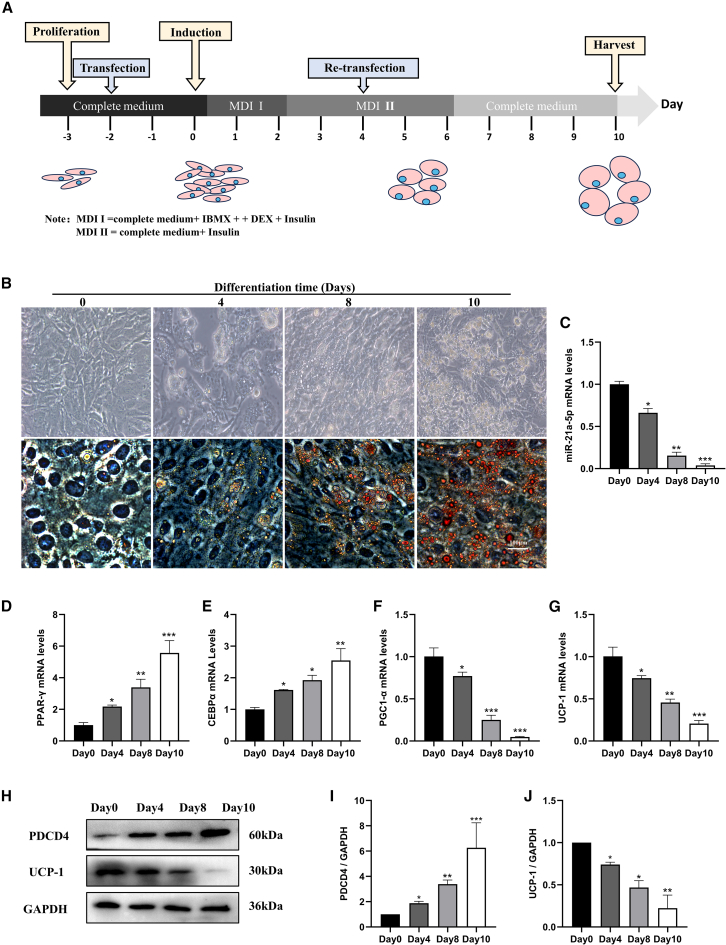
The induction, differentiation, and maturation process of 3T3-L1 cells and the changes in lipid metabolism (A) Flowchart of 3T3-L1 cell induced differentiation and transfection. (B) Representative Oil Red O staining images scale bars, 100 μm. (C) Expression levels of miR-21a-5p in 3T3-L1 cells. *n* = 3 biological replicates of each. Mean ± SD. ∗∗∗*p* < 0.001, ∗∗*p* < 0.01, and ∗*p* < 0.05 compared to the Day 0 group by one-way ANOVA with Tukey’s post hoc test. Gene expression of white adipokines PPAR-γ (D) and CEBPα (E), and brown adipokines PGC1α (F) and UCP-1 (G) in 3T3-L1 cells. *n* = 3 biological replicates of each. Mean ± SD. ∗∗∗*p* < 0.001, ∗∗*p* < 0.01, and ∗*p* < 0.05 compared to the Day 0 group by one-way ANOVA with Tukey’s post hoc test. (H) Representative western blotting images of PDCD4 and UCP-1. Quantitative analysis of PDCD4 (I) and UCP-1 (J). *n* = 3 biological replicates of each. Mean ± SD. ∗∗∗*p* < 0.001, ∗∗*p* < 0.01, and ∗*p* < 0.05 compared to the Day 0 group by one-way ANOVA with Tukey’s post hoc test. PPAR-γ stands for peroxisome proliferator-activated receptor-γ. CEBPα, CCAAT enhancer-binding protein α. UCP-1, mitochondrial uncoupling protein 1. PGC1-α, peroxisome proliferator-activated receptor gamma coactivator 1α. PDCD4, programmed cell death factor 4.

We subsequently assessed PDCD4 dynamics during 3T3-L1 differentiation triggered by the MDI cocktail. Oil Red O staining showed progressive lipid accumulation from day 0 to day 10 (Figures 4A and 4B). In line with adipogenesis, mRNA expression of white adipogenic markers (PPAR-γ and CEBPα) increased, while those of brown/beige markers (UCP-1 and PGC-1α) decreased (Figures 4D–4G). miR-21a-5p expression progressively declined throughout differentiation (Figure 4C). Western blot analysis further demonstrated that PDCD4 protein levels increased over time, mirroring lipid accumulation and contrasting with the downregulation of UCP-1 (Figures 4H–4J). These findings strongly suggest that PDCD4 serves as a regulatory factor in adipocyte differentiation and lipid metabolism.

### miR-21a-5p facilitates browning by targeting PDCD4 and triggering LXR-α/Akt signaling

**Figure 5:**
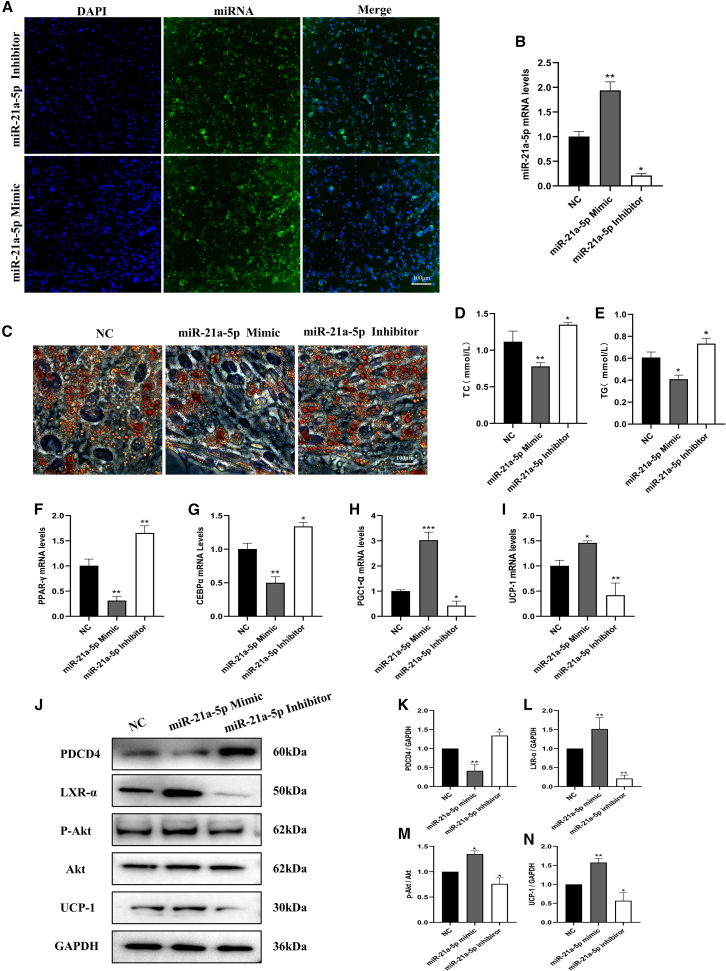
miR-21a-5p mimic promotes brown-like differentiation of 3T3-L1 cells by regulating PDCD4 and the downstream LXR-α/AKT signaling pathway (A) The transfection efficiency of miR-21a-5p mimic or inhibitor was detected by confocal microscopy. (B) miR-21a-5p expression in 3T3-L1 cells. *n* = 3 biological replicates of each. Mean ± SD. ∗∗*p* < 0.01 and ∗*p* < 0.05 compared to the NC group by Student’s *t* test. (C) Oil red O staining images scale bars, 100 μm. (D) TC contents and TG contents (E) in cell lysates. *n* = 3 biological replicates of each. Mean ± SD. ∗∗*p* < 0.01 and ∗*p* < 0.05 compared to the NC group by Student’s *t* test. (F and G) The expression of white adipokines PPAR-γ and CEBPα, as well as brown adipokines PGC1α and UCP-1 (H–I). *n* = 3 biological replicates of each. Mean ± SD. ∗∗∗*p* < 0.001, ∗∗*p* < 0.01, and ∗*p* < 0.05 compared to the NC group by Student’s *t* test. (J) Representative western blot images. (K) Quantitative analysis of PDCD4, LXR-α (L), Akt/p-Akt (M), and UCP-1 (N). *n* = 3 biological replicates of each. Mean ± SD. ∗∗*p* < 0.01 and ∗*p* < 0.05 compared to the NC group by Student’s *t* test. PPAR-γ, peroxisome proliferator-activated receptor-γ. CEBPα, CCAAT enhancer-binding protein α. UCP-1, mitochondrial uncoupling protein 1. PGC1-α, peroxisome proliferator-activated receptor gamma coactivator 1-alpha. PDCD4, programmed cell death factor 4. LXR-α, liver X receptor.

3T3-L1 cells were transfected with a miR-21a-5p mimic or inhibitor to evaluate its functional role. The successful transfection and overexpression of miR-21a-5p were validated (Figures 5A and 5B). The miR-21a-5p mimic led to a reduction in lipid droplet content and TC and TG levels, as shown in Figures 5C–5E. It also downregulated PPAR-γ and CEBPα mRNA while upregulating PGC-1α and UCP-1 (Figures 5F–5I). While the miR-21a-5p inhibitor elicited opposing effects.

The miR-21a-5p mimic downregulated PDCD4 levels, as revealed by western blot analysis (Figures 5J and 5K), while increased the protein levels of UCP-1, LXR-α, and phosphorylated Akt (Figures 5J, 5L, and 5N). Conversely, the miR-21a-5p inhibitor counteracted these effects. miR-21a-5p likely induces a beige-like adipocyte phenotype by inhibiting PDCD4, which activates the LXR-α/Akt pathway.

### Effects of PDCD4 knockdown (siPDCD4) on adipocyte browning in 3T3-L1 cells

**Figure 6:**
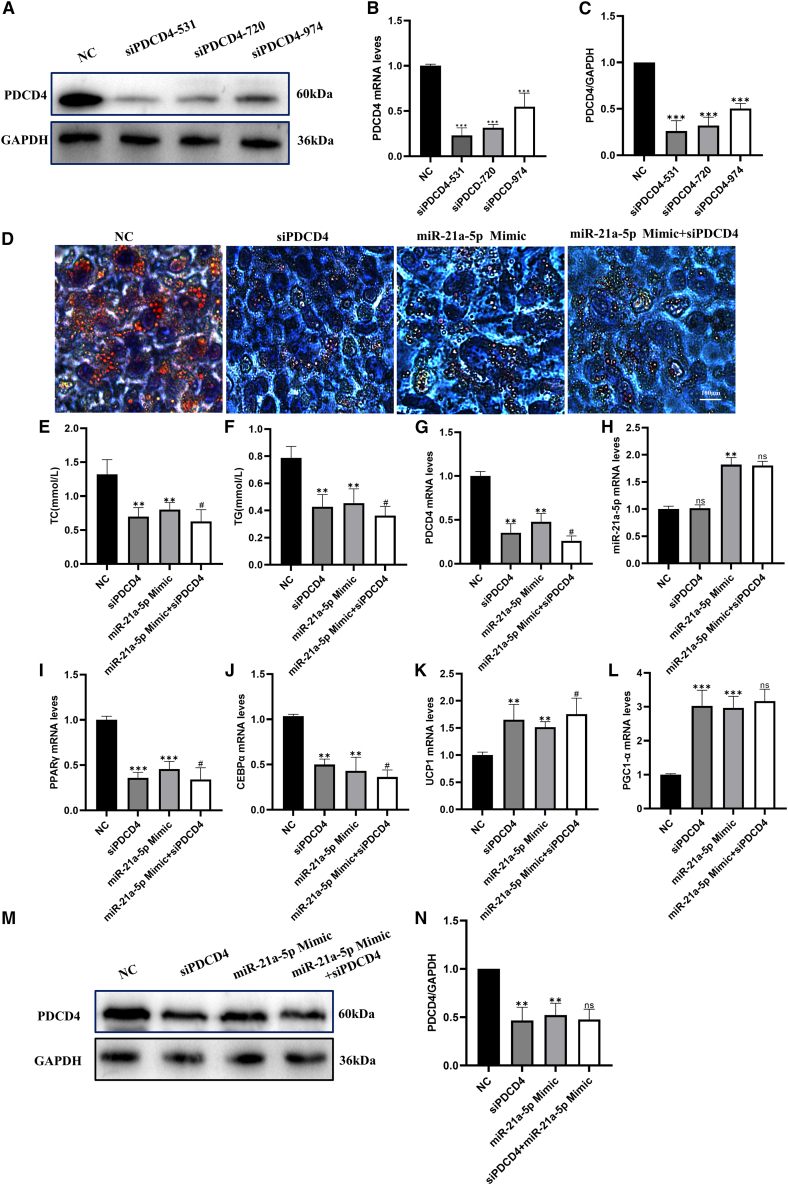
Effects of PDCD4 knockdown (siPDCD4) on adipocyte browning in 3T3-L1 cells (A) Silencing efficiency of siPDCD4 evaluated by western blot. (B) qPCR analysis of PDCD4 mRNA levels following siPDCD4 transfection. *n* = 4 biological replicates of each. Mean ± SD. ∗∗∗*p* < 0.001 compared with the NC group by Student’s *t* test. (C) Quantification of PDCD4 protein expression levels. *n* = 3 biological replicates of each. Mean ± SD. ∗∗∗*p* < 0.001 compared with the NC group by Student’s *t* test. (D) Oil red O staining of lipid droplets in each group. (E) TC levels and (F) TG levels in each group. *n* = 6 biological replicates of each. Mean ± SD. ∗∗*p* < 0.01 compared with the NC group, and ^#^*p* < 0.05 compared with the miR-21a-5p mimic group by Student’s *t* test. (G) PDCD4 mRNA expression levels, (H)miR-21a-5p expression levels, (I) PPARγ mRNA expression levels, (J) CEBPα mRNA expression levels, (K) UCP1 mRNA expression levels, (L) PGC-1α mRNA expression levels in each group. *n* = 4 biological replicates of each. Mean ± SD. ∗∗∗*p* < 0.001 and ∗∗*p* < 0.01 compared with the NC group, and ^#^*p* < 0.05 compared with the miR-21a-5p mimic group by Student’s *t* test. (M) Representative images of PDCD4 protein expression in each group. (N) Quantification of PDCD4 protein expression. *n* = 3 biological replicates of each. Mean ± SD. ∗∗*p* < 0.01 compared with the NC group by Student’s *t* test. PPAR-γ, peroxisome proliferator-activated receptor-γ. CEBPα, CCAAT enhancer-binding protein α. UCP-1, mitochondrial uncoupling protein 1. PGC1-α, peroxisome proliferator-activated receptor gamma coactivator 1-alpha. PDCD4, programmed cell death factor 4. TC, Total cholesterol. TG, Triglyceride.

To investigate the role of PDCD4 in adipocyte browning, we first validated the silencing efficiency of PDCD4-specific siRNA (siPDCD4) in 3T3-L1 adipocytes, and the results are shown in Figures 6A–6C. Following successful knockdown of PDCD4, we observed that siPDCD4 treatment significantly suppressed lipid accumulation in adipocytes, as demonstrated by reduced Oil Red O staining and TC/TG levels in Figures 6D–6F. Moreover, PDCD4 silencing markedly promoted the browning of adipocytes, evidenced by the changes in key browning-related marker expression presented in Figures 6I–6L. Notably, these effects induced by siPDCD4-namely reduced lipid aggregation and enhanced browning-closely resembled those observed following treatment with miR-21a-5p mimic alone, further supporting the notion that PDCD4 downregulation is a key event mediating the browning process.

### PDCD4 overexpression significantly reverses the effects of miR-21a-5p on adipocyte browning

**Figure 7:**
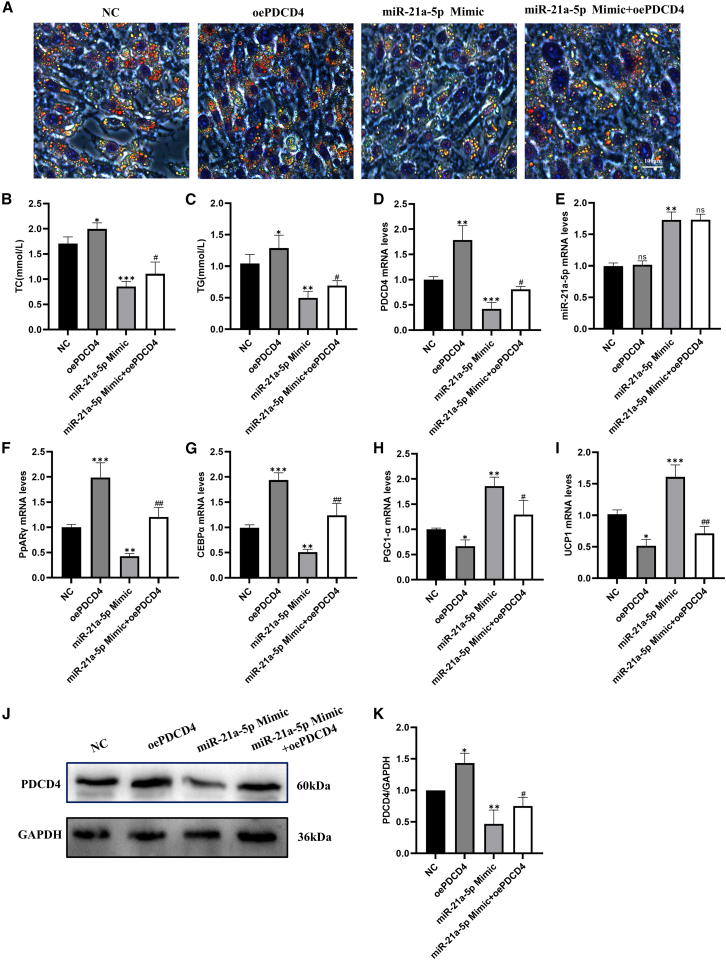
PDCD4 overexpression (oePDCD4) reverses the effects of miR-21a-5p on adipocyte browning in 3T3-L1 cells (A) Oil red O staining of lipid droplets in each group. (B) Total cholesterol (TC) levels in each group. (C) Triglyceride (TG) levels in each group. *n* = 6 biological replicates of each. Mean ± SD. ∗∗∗*p* < 0.001, ∗∗*p* < 0.01, and ∗*p* < 0.05 compared with the NC group, and ^#^*p* < 0.05 compared with the miR-21a-5p mimic group by Student’s *t* test. (D) PDCD4 mRNA expression levels, (E) miR-21a-5p expression levels, (F) PPARγ mRNA expression levels, (G) CEBPα mRNA expression levels, (H) PGC-1α mRNA expression levels, (I) UCP1 mRNA expression levels in each group. *n* = 4 biological replicates of each. Mean ± SD. (J) Representative images of PDCD4 protein expression in each group. ∗∗∗*p* < 0.001, ∗∗*p* < 0.01, and ∗*p* < 0.05 compared with the NC group, and ^##^*p* < 0.01, ^#^*p* < 0.05 compared with the miR-21a-5p mimic group by Student’s *t* test. (K) Quantification of PDCD4 protein expression. *n* = 3 biological replicates of each. Mean ± SD. ∗∗*p* < 0.01 and ∗*p* < 0.05 compared with the NC group, and ^#^*p* < 0.05 compared with the miR-21a-5p mimic group by Student’s *t* test. PPAR-γ, peroxisome proliferator-activated receptor-γ. CEBPα, CCAAT enhancer-binding protein α. UCP-1, mitochondrial uncoupling protein 1. PGC1-α, peroxisome proliferator-activated receptor gamma coactivator 1-alpha. PDCD4, programmed cell death factor 4.

To verify that PDCD4 downregulation is necessary for miR-21a-5p to promote adipocyte browning. We performed the rescue experiments by co-transfecting miR-21a-5p mimic with a PDCD4 overexpression vector into 3T3-L1 adipocytes. The results are presented in Figure 7. As expected, miR-21a-5p mimic alone significantly reduced lipid aggregation (Figures 7A–7C) and promoted adipocyte browning (increased PGC-1α and UCP-1 expression, downregulated PPAR-γ and CEBPα expression) (Figures 7F–7I). However, PDCD4 overexpression alone produced the opposite effect to the miR-21a-5p mimic, promoting lipid aggregation and inhibiting adipocyte browning. Importantly, co-expression of PDCD4 efficiently rescued PDCD4 expression and significantly attenuated the pro-browning effects induced by the miR-21a-5p mimic. These results demonstrate that PDCD4 downregulation is necessary for miR-21a-5p to promote adipocyte browning.

### PDCD4 is directly targeted of miR-21a-5p

**Figure 8:**
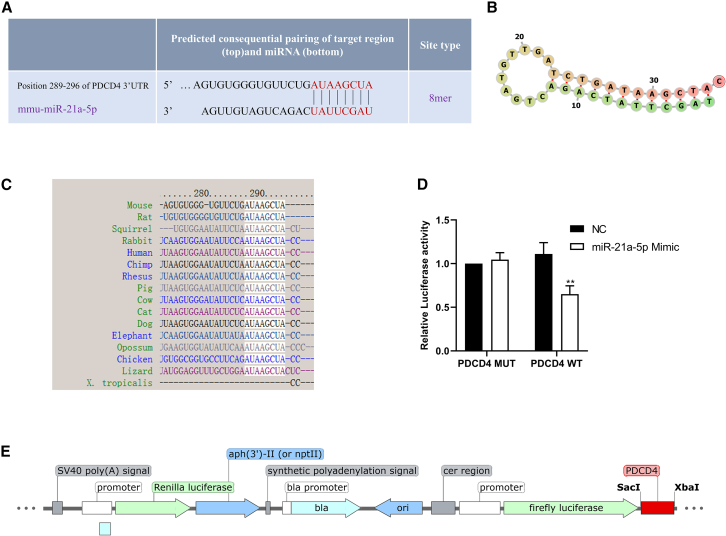
PDCD4 serves as a direct target of miR-21a-5p in 3T3-L1 cells (A) The binding targets of miR-21a-5p and PDCD4 were predicted using Target scan and miRNA walk. (B and C) The mature miR-21a-5p sequence is highly conserved across species. (D) Relative dual-luciferase report activity was assessed. *n* = 3 biological replicates of each. Mean ± SD. ∗∗*p* < 0.01 compared with the PDCD4-MUT+ NC group by Student’s *t* test. (E) A schematic of the dual luciferase reporter gene pmir-GLO-PDCD4 3′ UTR. NC, Negative Control. PDCD4-MUT, PDCD4 mutant plasmid. PDCD4-WT, PDCD4 wild-type plasmid.

Bioinformatic analysis with Target Scan and miRDB identified PDCD4 as a potential target of miR-21a-5p (Figures 8A–8C). To confirm this, the 3′-UTR sequences of PDCD4, either wild-type (WT) or mutant (MUT), were inserted into a pmirGLO dual-luciferase reporter vector (Figure 8E). Co-transfection of the miR-21a-5p mimic with the WT reporter significantly decreased luciferase activity, whereas the MUT reporter showed no change (Figure 8D). The data demonstrate that miR-21a-5p directly interacts with the 3′-UTR of PDCD4, highlighting its regulatory function in PDCD4 during 3T3-L1 browning differentiation.

## Discussion

This study demonstrates that ADSC-EXOs facilitate white fat browning, with miR-21a-5p from ADSC-EXOs potentially playing a key role by targeting PDCD4 and activating the LXR-α/Akt signaling pathway, influencing the browning differentiation of 3T3-L1 cells. Targeting ADSC-EXO-miR-21a-5p or PDCD4 could offer a promising pharmaceutical strategy for metabolic disease interventions.

Obesity arises from the buildup of systemic energy as lipids in white adipocytes, causing WAT deposition.^32^ Research indicates adipose tissue functions beyond merely storing fat, presenting potential therapeutic targets for metabolic disturbance.^33^ Adipose tissue comprises a diverse cellular composition: Approximately one-third consists of mature adipocytes, while the remaining two-thirds include a heterogeneous mix of precursor cells such as preadipocytes, mesenchymal stromal cells, immune cells (e.g., macrophages), and stromal vascular fraction (SVF) cells.^34^ These components form an intricate communication network that modulates adipose tissue expansion and function. Current obesity treatment strategies primarily focus on reducing energy intake, but these approaches have shown limited efficacy.^35^^,^^36^ Recent focus has shifted toward interventions that enhance energy expenditure by activating brown and beige adipocytes in adults, which are considered a promising alternative.^37^

WAT is distributed across the body in anatomically distinct depots, primarily located in the abdominal cavity and subcutaneous regions. Its expansion starts shortly after birth, and morphologically, WAT consists of large, unilocular lipid droplets with few mitochondria, giving it an ivory or yellowish appearance. In contrast, BAT is far less abundant in humans and diminishes with age, persisting mainly near deeper organs. BAT is distinguished by its multiple small lipid droplets, high mitochondrial density, and brown color attributed to the hemoglobin cofactor in cytochrome oxidase.^38^ Brown adipocytes generate heat through UCP-1, a mitochondrial UCP (proton channel) that dissipates the proton gradient to produce heat instead of ATP.^39^ Beige adipocytes, expressing UCP-1, can develop thermogenic properties akin to brown adipocytes within WAT when exposed to certain environmental, genetic, or pharmacological stimuli.^40^ This adaptive process, known as WAT browning, allows beige fat to transform chemical energy into heat, similar to the metabolic function of BAT.

To investigate obesity-related metabolic regulation, we developed an *in vivo* obese mouse model through HFD feeding and an *in vitro* preadipocyte differentiation model using 3T3-L1 cells. Our study demonstrated that ADSC-EXOs significantly reduced lipid accumulation in the adipose tissue of HFD-fed mice and decreased serum TC and TG levels. Adipogenesis is mainly controlled by transcription factors such as PPAR-γ and CEBPα,^41^ while UCP-1 and PGC-1α are crucial for BAT thermogenesis and WAT browning.^42^ Notably, UCP-1, the dominant thermogenic protein in BAT, serves as a critical driver of adaptive thermogenesis.^43^ Consistent with previous studies,^44^ we observed that ADSC-EXOs modulate adipogenesis and thermogenesis by upregulating UCP-1 and PGC-1α expression, while downregulating PPAR-γ and CEBPα expression. The above results indicate that ADSC-EXOs mitigate obesity-associated lipid accumulation and dyslipidemia.

Extensive research has demonstrated that miRNAs are vital regulators in biological processes.^45^^,^^46^ miRNAs can be secreted through exosomes, functioning as key regulators in intercellular signaling and tissue function modulation.^47^ Notably, adipose-derived exosome miRNAs can exert systemic effects by influencing gene expression in distant tissues.^13^ Of particular interest are miRNAs that regulate adipose plasticity and function.^48^ A more comprehensive understanding of miRNA-mediated regulation of BAT thermogenesis and WAT browning processes could reveal novel therapeutic targets for combating obesity and related metabolic disturbance. ADSCs generate exosomes with elevated levels of miR-21 and miR-21a-5p expression.^49^^,^^50^^,^^51^ To further explore these mechanisms, we utilized 3T3-L1 cells, a widely recognized *in vitro* adipogenesis model. After inducing differentiation using the MDI protocol (IBMX, dexamethasone, and insulin),^52^ we tracked miR-21a-5p expression during adipogenesis. During 3T3-L1 differentiation (days 0–10), miR-21a-5p levels progressively decreased, aligning with reduced thermogenic markers (UCP-1, PGC-1α) and elevated expression of lipogenic genes (PPAR-γ, CEBPα) in mature adipocytes. The findings indicate a strong likelihood of miR-21a-5p′s involvement in adipocyte differentiation and metabolic reprogramming. During 3T3-L1 differentiation, transfection with a miR-21a-5p mimic or inhibitor revealed that the mimic significantly reduced PPAR-γ and CEBPα levels while increasing UCP-1 and PGC-1α, aligning with *in vivo* findings. This indicates that miR-21a-5p inhibits adipogenesis and enhances thermogenic potential. Our findings underscore the therapeutic potential of ADSC-EXOs and miR-21a-5p in regulating adiposity and promoting fat browning.

PDCD4 has been implicated in various inflammatory metabolic disorders. Research indicates that a lack of PDCD4 alleviates diet-induced obesity, WAT inflammation, insulin resistance, and improves hepatic lipid accumulation.^28^^,^^53^ Elevated PDCD4 levels could play a role in the development of polycystic ovary syndrome (PCOS) by influencing metabolic issues such as obesity, insulin resistance, and dyslipidemia.^54^ The results suggest that PDCD4 could be an effective therapeutic target for metabolic diseases associated with obesity. PDCD4 prevents NASH progression in mice by suppressing CIITA-driven MHCII expression in hepatocytes, thereby blocking their transformation into antigen-presenting cells that activate CD4^+^ T cells and promote inflammation and lipid accumulation.^55^ These findings suggest that PDCD4 exerts context-dependent, tissue-specific roles in metabolic diseases-protecting against NASH by acting within hepatocytes, while its deficiency improves systemic obesity and insulin resistance through effects on adipose and immune cells.

LXR-α is prominently enriched in metabolically active tissues such as liver, intestine, kidney, spleen, and adipose tissue.^56^^,^^57^ LXR-α is a crucial transcriptional regulator essential for cholesterol and fatty acid metabolism, lipid homeostasis, and increased energy expenditure.^58^^,^^59^ These data raise the possibility that the suppression of LXR-α expression serves as a central mechanism through which PDCD4 dysregulates metabolic homeostasis, a hypothesis that merits further investigation.

We analyzed PDCD4 protein expression and LXR-α/Akt pathway components in inguinal adipose tissue and MDI-differentiated 3T3-L1 cells to determine if miR-21a-5p promotes fat browning via PDCD4 and its downstream pathways. The study found increased PDCD4 expression in inguinal adipose tissue and differentiated 3T3-L1 adipocytes, while LXR-α expression and Akt phosphorylation levels were decreased. This pattern was reversed by treatment with ADSC-EXOs.

Transfection with a miR-21a-5p mimic reduced PDCD4 expression and enhanced phosphorylation of LXR-α and Akt, while transfection with a miR-21a-5p inhibitor or negative controls showed no such effects. Knockdown of PDCD4 by siRNA alone significantly promoted adipocyte browning. Conversely, the browning effect mediated by miR-21a-5p was markedly attenuated upon co-transfection with a PDCD4-overexpressing vector. These results establish that PDCD4 downregulation is essential for miR-21a-5p to induce adipocyte browning. The dual-luciferase reporter assay verified that miR-21a-5p directly targets the 3′UTR of PDCD4. These findings suggest that miR-21a-5p mitigates obesity by targeting PDCD4, which activates the LXR-α/Akt/UCP-1 signaling pathway and facilitates white-to-beige adipocyte trans differentiation in 3T3-L1 cells.

Collectively, this study shows that ADSC-EXOs facilitate the browning of WAT by delivering miR-21a-5p, which inhibits 3T3-L1 adipocyte differentiation and reduces obesity. miR-21a-5p targets PDCD4, activating the LXR-α/Akt signaling pathway. The study indicates that ADSC-EXOs and PDCD4 could serve as therapeutic targets, with promoting white-to-beige fat transdifferentiation offering a promising approach for treating obesity and related metabolic disorders.

### Limitations of the study

In this study, the functional role of miR-21a-5p was examined only at the cellular level using *in vitro* overexpression experiments, without *in vivo* validation. Although ADSC-Exos exert significant effects in mice, it remains unclear whether these effects are dependent on miR-21a-5p. Future studies employing adipose-specific overexpression of miR-21a-5p *in vivo* are warranted to further establish its causal role and physiological relevance.

The present findings were obtained using the 3T3-L1 cell line, and whether miR-21a-5p exerts similar pro-thermogenic effects in primary subcutaneous or brown adipocytes remains to be determined. Future studies using primary adipocyte cultures are warranted to further validate the physiological role of miR-21a-5p in thermogenesis.

In this study, we exclusively employed male mice to avoid potential interference from estrogen cycle fluctuations in female mice. However, the lack of female animal cohorts prevents us from assessing sex-related effects on our data, and we recognize this as an important limitation. Subsequent studies will include both sexes to fully clarify sex-dependent differences.

## Resource availability

### Lead contact

Further information and requests for resources should be directed to and will be fulfilled by the lead contact, Dr. Jingxia Du (dujingxia2005@163.com).

### Materials availability

Data and materials from the study will be available upon request of the lead contact.

### Data and code availability

• All data reported in this paper will be shared by the lead contact upon request.
• This paper does not report original code.
• Any additional information required to reanalyze the data reported in this paper is available from the lead contact upon request.

## Acknowledgments

This research was funded by the Henan Province Scientific and Technology Research Project (grant no. 262102310193), and College Student Innovation and Entrepreneurship Training Program of Henan Province (grant no. S202510464081).

## Author contributions

C.X.: established of the pathological models in mice and cell, molecular biological experiment, original draft. Y.H.: validation, gain- and loss-of-function, molecular biological experiment, formal analysis, data curation. T.W.: established of the pathological models in mice. B.Y.: investigation, abstract diagram. Y.W.: validation. H.S.: molecular biological experiment. Y.L.: data analysis. G.L.: manuscript editing, project administration. J.D.: supervision, securing funding, manuscript review. All authors read and approved the final manuscript for submission.

## Declaration of interests

The authors declare no competing interests.

## STAR★Methods

### Key resources table

**Table:** 

REAGENT or RESOURCE	SOURCE	IDENTIFIER
**Antibodies**		

UCP1(E9Z2V) XP Rabbit mAb	Cell Signaling Technology	Cat#72298 S; RRID: AB_2936479
PDCD4(D29C6) XP Rabbit mAb	Cell Signaling Technology	Cat#9535 S; RRID: AB_2162318
NR1H3 (LXR-α) Polyclonal antibody	Proteintech	Cat#14351-1-AP; RRID‌: AB_10640525
Phospho-Akt (Ser473) (D9E) Rabbit Monoclonal Antibody	Cell Signaling Technology	Cat#4060 S; RRID‌: AB_2315049
Akt (pan) (C67E7) Rabbit Monoclonal Antibody	Cell Signaling Technolog	Cat#4691 S; RRID‌: AB_915783
Anti-TSG101 Rabbit pAb	Wanleibio	Cat#WL05130; RRID: AB_3699299
Anti-CD63 Rabbit pAb	Wanleibio	Cat#WL02549; RRID: AB_2910631
Anti-GAPDH HRP conjugated Antibody	Boster	Cat#BM3896; RRID: AB_3166181
HRP-conjugated Affinipure Goat Anti-Rabbit lgG(H + L)	Proteintech	Cat#SA00001-2; RRID‌: ‌AB_2722564

**Biological samples**		

Adipose-derived Mesenchymal Stem Cells (ADSCs)	Isolated from healthy mouse adipose tissue	N/A

**Chemicals, peptides, and recombinant proteins**		

DMEM/F12	HyClone	Cat#SH30272.01
Fetal bovine serum (FBS)	Gibco	Cat#A5669701
3T3-L1 specific medium	Yuchi Biology	Cat#CXS1058
IBMX	Sigma-Aldrich	Cat#I5879
Dexamethasone	Sigma-Aldrich	Cat#D2915
Insulin	Sigma-Aldrich	Cat#91077C
Lipofectamine™ RNAi MAX	Thermo Fisher Scientific	Cat#15338500
TRIzol reagent	Thermo Fisher Scientific	Cat#15596026CN
SYBR Green PCR Master Mix	CWBio	Cat#CW3397S
RIPA buffer	Solarbio	Cat#R0010
BCA assay kit	Beyotine	Cat#P0006
Lipofectamine 2000	Thermo Fisher Scientific	Cat#11668500

**Critical commercial assays**		

Blood glucose monitor	Roche Diagnostics	ACCU-CHEK Performa test strips
Serum glucose assay kit	Sangon Biotech	D799405
Serum insulin assay kit	XY-Bioscience	XY-E20353
Total cholesterol (TC) assay kit	Nanjing Jiancheng Bioengineering Institute	Cat#A111-1-1
Triglyceride (TG) assay kit	Nanjing Jiancheng Bioengineering Institute	Cat#A110-1-1
Dual-luciferase reporter assay kit	Vazyme	Cat#DL101-01

**Experimental models: Cell lines**		

3T3-L1 Cells	CAS Cell Bank	RRID: SCSP-5038
293 T Cells	Keycell	RRID: QS-H164

**Experimental models: Organisms/strains**		

C57BL/6 male	Huazhong University of Science and Technology	N/A

**Oligonucleotides**		

Mouse-derived mmu-miR-21a-5p mimics sequence: UAGCUUAUCAGACUGAUGUUGA AACAUCAGUCUGAUAAGCUAUU	This paper	N/A
Mouse-derived mmu-miR-21a-5p inhibitor sequence: UCAACAUCAGUCUGAUAAGCUA	This paper	N/A
siRNA targeting sequence: Pdcd4-Mus-531: GGAAGUCAAGCGGUUAGAATT UUCUAACCGCUUCACUUCCTT	This paper	N/A
PDCD4 overexpression vector	GenePharma	N/A
Primers for UCP-1: F: AAACAGAAGGATTGCCGAAACT R: CTCTGTAGGCTGCCCAATGAA	This paper	N/A
Primers for PGC1α: F: CTGGGTGGATTGAAGTGGTGTA R: AGTGGTCACGGCTCCATCTGT	This paper	N/A
Primers for PPAR-γ: F: GACCACTCGCATTCCTTTGACA R: ATCGCACTTTGGTATTCTTGGA	This paper	N/A
Primers for CEBPα: F: TCGGTGGACAAGAACAGCAACG R: CGGTCATTGTCACTGGTCAACTCC	This paper	N/A
Primers for U6: F: AACAGTGCTCGCTTCGGCAG RT:GTCGTATCCAGTGCAGGGTCCGA GGTATTCGCACTGGATACGACTGTGCT	This paper	N/A
Primers for miR-21a-5p: F: CGGCTAGCTTATCAGACTGA RT:GTCGTATCGACTGCAGGGTCCGA GGTATTCGCAGTCGATACGACTCAACA	This paper	N/A
Primers for β-actin: F: GTGACGTTGACATCCGTAAAGA R: GCCGGACTCATCGTACTCC	This paper	N/A

**Recombinant DNA**		

PDCD4 3′UTR wild-type (WT) plasmid	This paper	N/A
PDCD4 3′UTR mutant (MUT) plasmid	This paper	N/A
pmirGLO dual-luciferase reporter vector	Vazyme	N/A

**Software and algorithms**		

GraphPad Prism 9.0	Dotmatics	https://www.graphpad.com
ImageJ	Schneider et al.^1^	https://imagej.nih.gov/ij/
TargetScan	TargetScan	https://www.targetscan.org
miRDB	miRDB	http://www.mirdb.org

**Other**		

Sorvall™ WX + ultracentrifuge	Thermo Fisher Scientific	N/A
Transmission electron microscope (80 kV)	Thermo Fisher Scientific (FEI)	Model: Talos F200S
Nanoparticle tracking analyzer	Malvern	Model: NANOZSE
7500 Sequence Detection System	Applied Biosystems	N/A
Oil Red O solution	Solarbio	Cat#G1260
Hematoxylin	Solarbio	Cat#G1080
PVDF membrane	Merck Millipore Ltd	Cat# ISEQ00010
Enhanced chemiluminescence system	Tanon Science Technology Co., Ltd	Model: Tanon-4600

### Experimental model and study participant details

#### Ethical committee approving statement

The animal experiments were approved by the Institutional Animal Care and Use Committee of Henan University of Science and Technology (Licence No. HAUST-025-M0221052), all the procedures were conducted in compliance with the NIH Guide for the Care and Use of Laboratory Animals.

#### Experimental animals

Male C57BL/6 mice (4–5 weeks old, initial weight 15 ± 2 g) were housed in controlled environment (22 ± 2°C, 50 ± 5% humidity) with a 12-h light/dark cycle.

### Method details

#### Establishment of animal models fed with HFD

Following a seven-day acclimatization, the mice were randomly divided into three groups (*n* = 6 each): normal-fat diet (NFD), high-fat diet (HFD), and high-fat diet plus ADSC-EXOs. The HFD, composed of 60% kcal fat, 20% carbohydrate, and 20% protein, was provided continuously for 20 weeks to all groups except the NFD group, which received a standard chow diet containing 10% fat. In the last four weeks of the study, mice in the HFD + ADSC-EXOs group were administered ADSC-EXOs via tail vein injections (50 μg per injection) twice weekly, similarly, the mice in the NFD group and the HFD group were injected with an equal volume of PBS via tail vein.

#### Isolation and characterization of ADSC-EXOs

The isolation and culture techniques for ADSCs followed the protocol outlined in a prior study.^60^ ADSCs were maintained in DMEM/F12 (HyClone, USA) with 10% FBS (Gibco, USA) at 37°C and 5% CO_2_. The cell culture supernatant was collected for exosome isolation using ultracentrifugation. The supernatant underwent initial centrifugation at 2,000 × g for 10 min at 4°C to eliminate non-adherent cells. The supernatant was pelleted by centrifugation (10,000 × g, 30 min, 4°C) to eliminate cellular debris, then subjected to ultracentrifugation (10,000 × g, 90 min, 4°C) using a Sorvall WX + ultracentrifuge (Thermo Fisher Scientific). The ADSC-EXOs pellet was reconstituted in PBS, sterile-filtered through a 0.22 μm membrane, and aliquoted for storage at −80°C for future analysis after the supernatant was removed. Transmission electron microscopy at 80 kV was used to examine the morphology of ADSC-EXOs. Particle size distribution was assessed using nanoparticle tracking analysis (NTA). Western blotting was used to detect specific exosomal protein markers.

#### Oral glucose tolerance test (OGTT) measurements

In the 19th week of the experiment, an oral glucose tolerance test (OGTT; 0.2 g/kg) was conducted. Blood samples were collected via the tail artery at intervals of 0, 30, 60, and 120 min following the oral administration of glucose. Plasma glucose concentrations were subsequently measured using a blood glucose monitor (Roche Diagnostics). The area under the curve (AUC) was calculated using the approximate trapezoidal area formula, which more accurately reflects the trends and cumulative effects over time of blood glucose levels. Following the OGTT, the animals in each group continued to reside in their respective conditions, as previously described, until the 20th week.

#### Culture and differentiation of 3T3-L1 cells

3T3-L1 cells (CAS Cell Bank, SCSP-5038, China) were grown in a humified incubator at 37°C with 5% CO_2_ and cultured in 3T3-L1 specific medium (Yuchi Biology, CXS1058, China). Upon reaching around 90% confluence, cells underwent differentiation via the MDI protocol, involving a 2-day incubation in complete medium with 0.5 mM IBMX, 1 μM dexamethasone, and 10 μg/mL insulin (Day 2). The medium was replaced with a complete medium containing 10 μg/mL insulin for 4 days (Day 6). Finally, cells were maintained in complete medium without induction agents for an additional 4 days (Day 10). Under these conditions, >90% of the cells exhibited a mature adipocyte phenotype. 3T3-L1 cell lines used in this study were fully authenticated and mycoplasma-negative. Cell authentication and mycoplasma contamination testing were performed by GENETIC TESTING BIOTECHNOLOGY. Short tandem repeat (STR) profiling was adopted for cell line identity verification, and mycoplasma detection was conducted via PCR-based assay.

#### Cell transfection

3T3-L1 cells were seeded in six-well plates and differentiated following the classical MDI protocol described above. Transfection was conducted on days 0 and 5 of differentiation using Lipofectamine RNAi MAX (Invitrogen, Thermo Fisher Scientific) as per the manufacturer’s guidelines, with 30 nM of miR-21a-5p mimic or inhibitor. PDCD4 overexpression vector or PDCD4 siRNA were transfected as group needed. After completing the differentiation process on day 10, cells were harvested for subsequent morphological and molecular analyses.

#### Oil red O staining of differentiated 3T3-L1 cells

Mature 3T3-L1 cells were fixed with 4% formaldehyde for 30 min at room temperature, following the specified differentiation protocol. Cells were stained with 0.3% Oil Red O solution for 30 min to visualize lipid droplets. Sections were then counterstained with hematoxylin for 2 min to visualize nuclei. Images were captured under a microscope to assess lipid deposition.

#### Quantification of serum metabolic parameters

Fasting serum glucose and insulin levels were measured using commercial assay kits (Sangon Biotech and XY-Bioscience, China), and the homeostasis model assessment of insulin resistance (HOMA-IR) was calculated for each group. Serum total cholesterol (TC) and triglycerides (TG) levels were determined using commercial kits in accordance with the manufacturer’s guidelines (Nanjing Jiancheng Bioengineering Institute, China). Differentiated 3T3-L1 cells were homogenized and centrifuged, and the supernatant were used to measure cellular TC and TG levels following the same protocol as for serum samples.

#### Histological analysis of inguinal adipose tissue

Inguinal adipose tissue samples were fixed in 4% formaldehyde, paraffin-embedded, and sectioned at 4 μm for morphological analysis. Tissue sections underwent deparaffinization and rehydration; subsequently, standard hematoxylin and eosin (H&E) staining was applied, and imaged using a light microscope.

For immunohistochemical staining, following antigen retrieval in citrate buffer (pH 6.0) at 97°C for 20 min, deparaffinized sections were incubated overnight at 4°C with primary antibodies targeting UCP-1 (1:1000, CST #72298 S) or PDCD4 (1:1000, CST #9535 S). Following primary antibody incubation, sections were exposed to an HRP-conjugated secondary antibody for 30 min. Signal detection was performed using DAB substrate, followed by counterstaining with hematoxylin.

#### Quantitative real-time PCR (RT-qPCR)

Gene expression of miR-21a-5p and adipokine markers was assessed via RT-qPCR using a 7500 Sequence Detection System (Applied Biosystems, USA) and an SYBR Green PCR Master Mix (CWBio, China). Total RNA was isolated using the TRIzol reagent and subsequently reverse-transcribed into cDNA. The reverse transcription reaction was carried out under the following thermal conditions: 25°C for 10 min; 50°C for 15 min; and 85°C for 5 min miR-21a-5p expression was normalized using U6 as the internal control.

The expression levels of white adipogenic markers, such as peroxisome proliferator-activated receptor-γ (PPAR-γ) and CCAAT enhancer binding protein α (CEBPα), along with brown/beige adipogenic markers like mitochondrial uncoupling protein 1 (UCP-(1) and peroxisome proliferator-activated receptor γ coactivator 1-α (PGC1-α), were assessed. Relative quantification was performed using the 2^−ΔΔCt^ method, with β-actin serving as the endogenous reference.

#### Western blot analysis

Total protein was extracted from exosomes, adipose tissue, or 3T3-L1 cells using ice-cold RIPA buffer containing protease inhibitor, and then the concentration was quantified using the BCA assay. Samples were resolved on 10% SDS-PAGE gels at 80 V for 1.5 h at room temperature and subsequently transferred to PVDF membranes (300 mA, 2 h, 4°C). Membranes were blocked using 5% BSA in TBST (0.5% Tween 20) for 2 h at room temperature, then incubated overnight at 4°C with primary antibodies diluted in the same blocking solution. Antibodies used as following: UCP-1 (1:1000; CST #72298 S), PDCD4 (1:1000; CST #9535 S), LXR-α (1:2000; Proteintech 14351-1-AP), Akt (1:1000; CST #4691 S), phospho-Akt (1:3000; CST #4060 S), CD63 (1:1000; Wanleibio WL02549), TSG101 (1:1000; Wanleibio WL05130), and GAPDH (1:5000; Boster BM3896).

Following three washes with TBST, membranes were probed with HRP-conjugated second

ary antibodies (1:5000, 1 h, room temperature), then immunoreactive bands were detected using an enhanced chemiluminescence system (Amersham). The images were analyzed and quantified using ImageJ software.

#### Dual-luciferase reporter assay

TargetScan and miRDB software identified PDCD4 as a predicted target of miR-21a-5p. The wild-type (WT-PDCD4) and mutant (MUT-PDCD4) 3′ UTR fragments of PDCD4 were cloned into the pmirGLO dual-luciferase reporter vector. HEK-293 T cells were transfected with Lipofectamine 2000 using either miR-21a-5p mimic or negative control (NC) alongside WT-PDCD4 or MUT-PDCD4 reporter plasmids. After 48 h, cells were lysed and luciferase activity was measured with a dual-luciferase assay kit.

### Quantification and statistical analysis

GraphPad Prism 9.0 was used to produce graphical representations of data. Data are expressed as mean ± SD. Multiple group comparisons were conducted using one-way ANOVA with Tukey’s post hoc test in GraphPad Prism 9.0 (Dotmatics). While Student’s *t*-Tests of two-tailed and unpaired with equal variance was employed for comparisons between two groups. A *p*-value of less than 0.05 was deemed statistically significant.

## Supplemental information

Data S1. Raw experimental data generated in this study
