# Supplementary material for: A cell-free browning strategy: Exosomal miR-21a-5p from ADSCs targets PDCD4 to reshape adipose metabolism
Source: iScience. 2026 Jul 14;29(8):116765. doi: 10.1016/j.isci.2026.116765 (PMC13382807; doi:10.1016/j.isci.2026.116765)
Supplement: Data S1. Raw experimental data generated in this study [file mmc1.zip › All original data/qPCR related data/qPCR primer sequence.docx]

**Primer sequences used in qPCR analysis of mouse**

| **Gene** | **Sequence** |
| --- | --- |
| UCP-1 | Forward: 5’- AAACAGAAGGATTGCCGAAACT-3’ |
|  | Reverse: 5’-CTCTGTAGGCTGCCCAATGAA-3’ |
| PGC1α | Forward: 5’- CTGGGTGGATTGAAGTGGTGTA-3’ |
|  | Reverse: 5’- AGTGGTCACGGCTCCATCTGT-3’ |
| PPAR-γ | Forward: 5’-GACCACTCGCATTCCTTTGACA-3’ |
|  | Reverse: 5’- ATCGCACTTTGGTATTCTTGGA-3’ |
| CEBPα | Forward: 5′-TCGGTGGACAAGAACAGCAACG-3′ |
|  | Reverse:5′-CGGTCATTGTCACTGGTCAACTCC-3′ |
| U6 | F: AACAGTGCTCGCTTCGGCAG |
|  | RT:GTCGTATCCAGTGCAGGGTCCGAGGTATTCGCACTGGATACGACTGTGCT |
| miR-21a-5p | F: CGGCTAGCTTATCAGACTGA |
|  | RT:GTCGTATCGACTGCAGGGTCCGAGGTATTCGCAGTCGATACGACTCAACA |
| β-Actin | Forward: 5′- GTGACGTTGACATCCGTAAAGA-3′ |
|  | Reverse: 5’- GCCGGACTCATCGTACTCC-3’ |
