# Supplementary material for: A cell-free browning strategy: Exosomal miR-21a-5p from ADSCs targets PDCD4 to reshape adipose metabolism
Source: iScience. 2026 Jul 14;29(8):116765. doi: 10.1016/j.isci.2026.116765 (PMC13382807; doi:10.1016/j.isci.2026.116765)
Supplement: Data S1. Raw experimental data generated in this study [file mmc1.zip › All original data/WB related data/WB Original images.pdf]

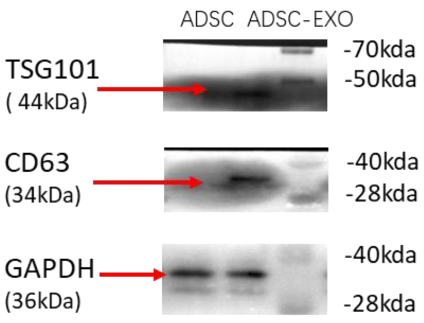

Original images for Western blotting for TSG101 and CD63 in Figure 1.

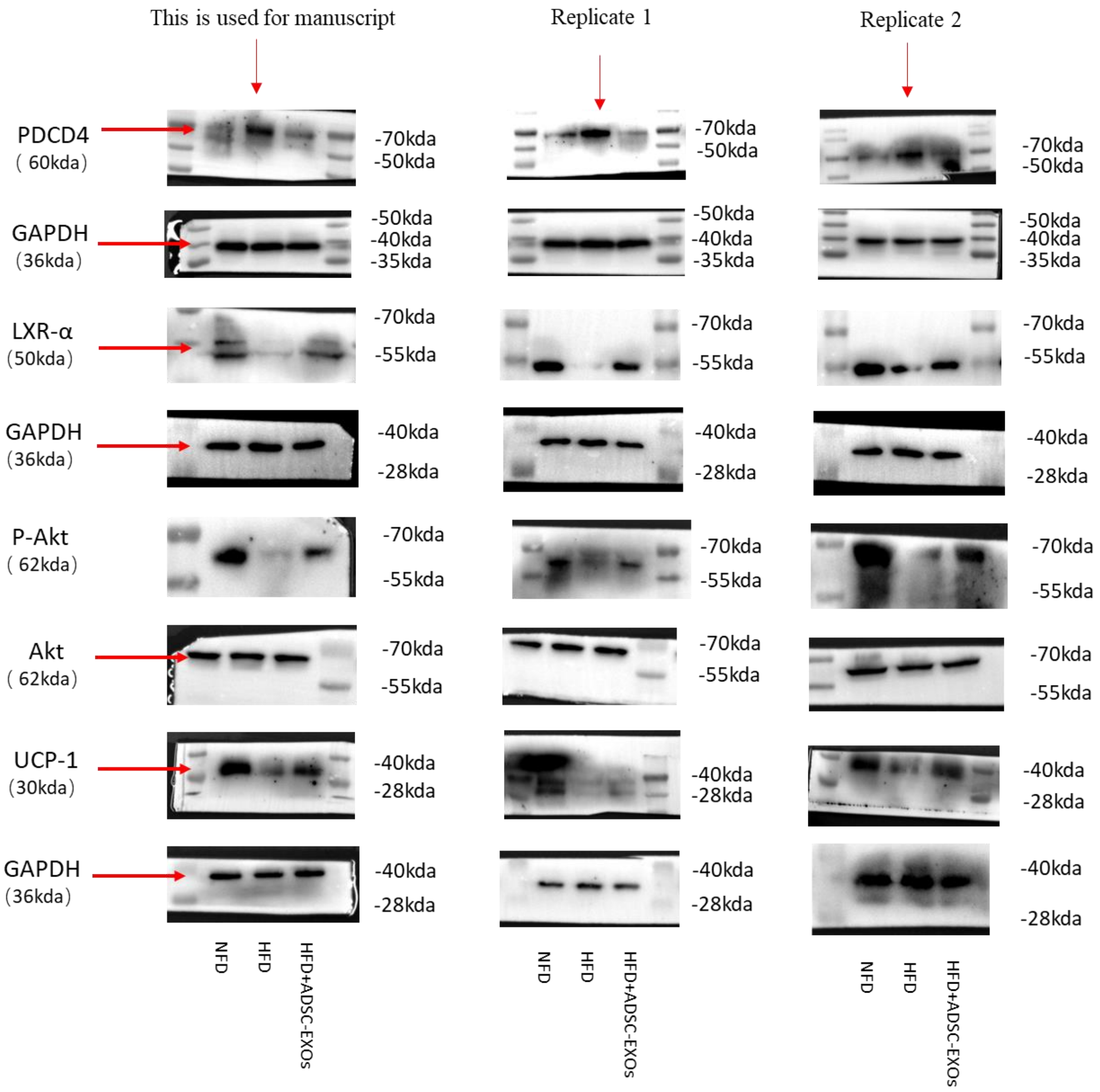

Original images for Western blotting for PDCD4、LXR-α、p-Akt/Akt and UCP-1 in Figure 3.

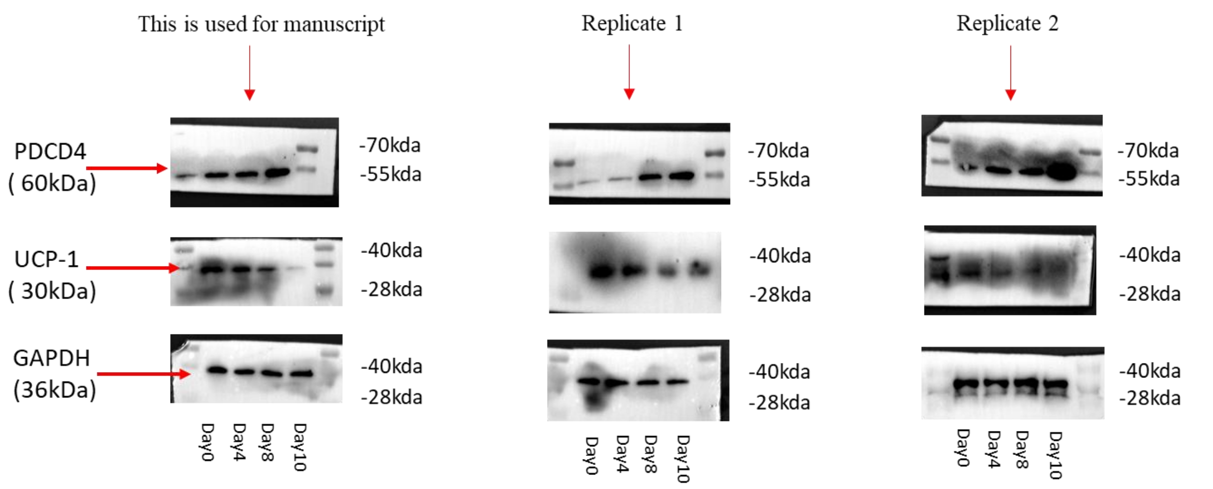

Original images for Western blotting for PDCD4 and UCP-1 in Figure 4.

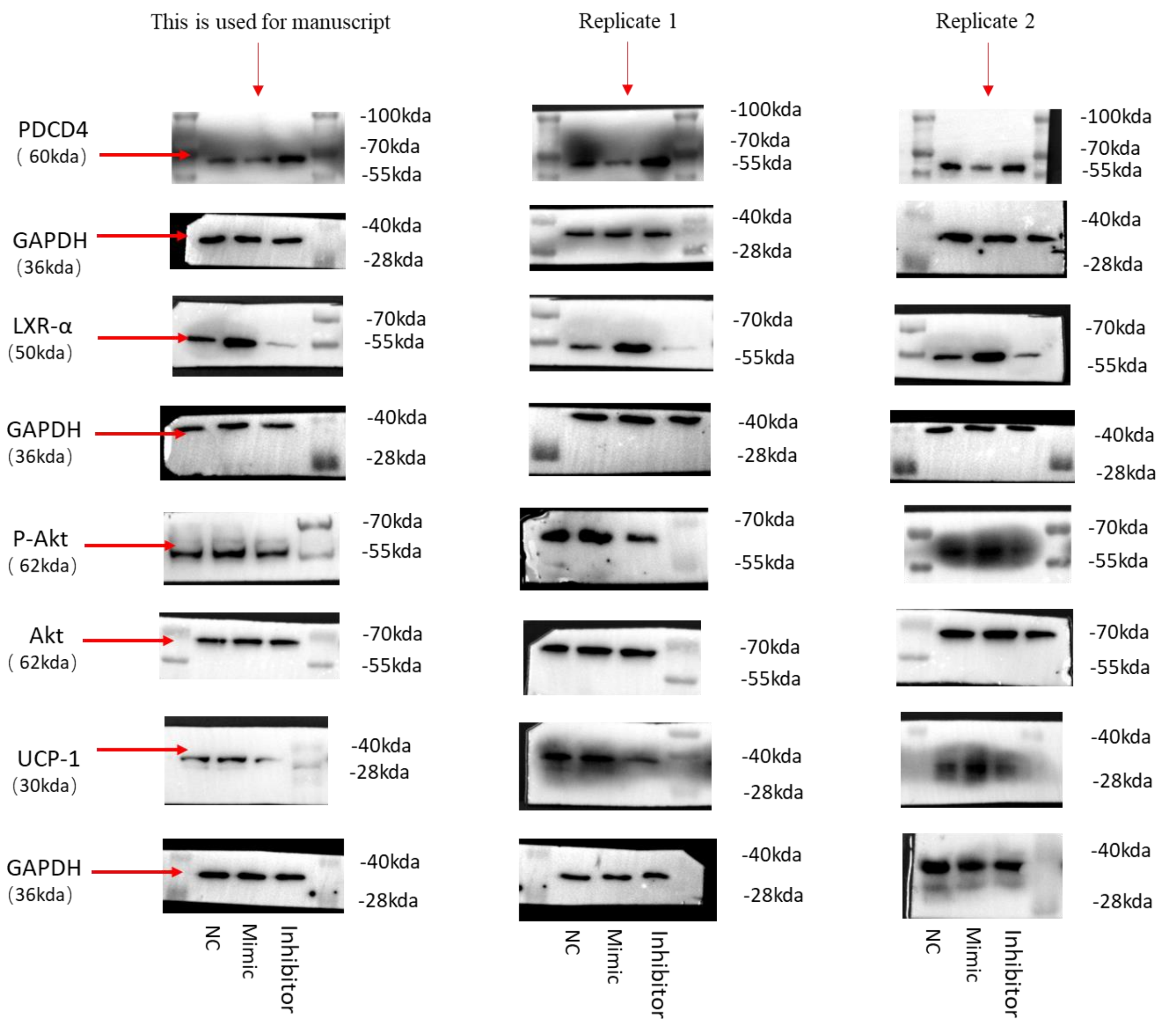

Original images for Western blotting for PDCD4、LXR-α、p-Akt/Akt and UCP-1 in Figure 5.
