# Supplementary figures and images for: A cell-free browning strategy: Exosomal miR-21a-5p from ADSCs targets PDCD4 to reshape adipose metabolism (part 2 of 2)
Source: iScience. 2026 Jul 14;29(8):116765. doi: 10.1016/j.isci.2026.116765 (PMC13382807; doi:10.1016/j.isci.2026.116765)

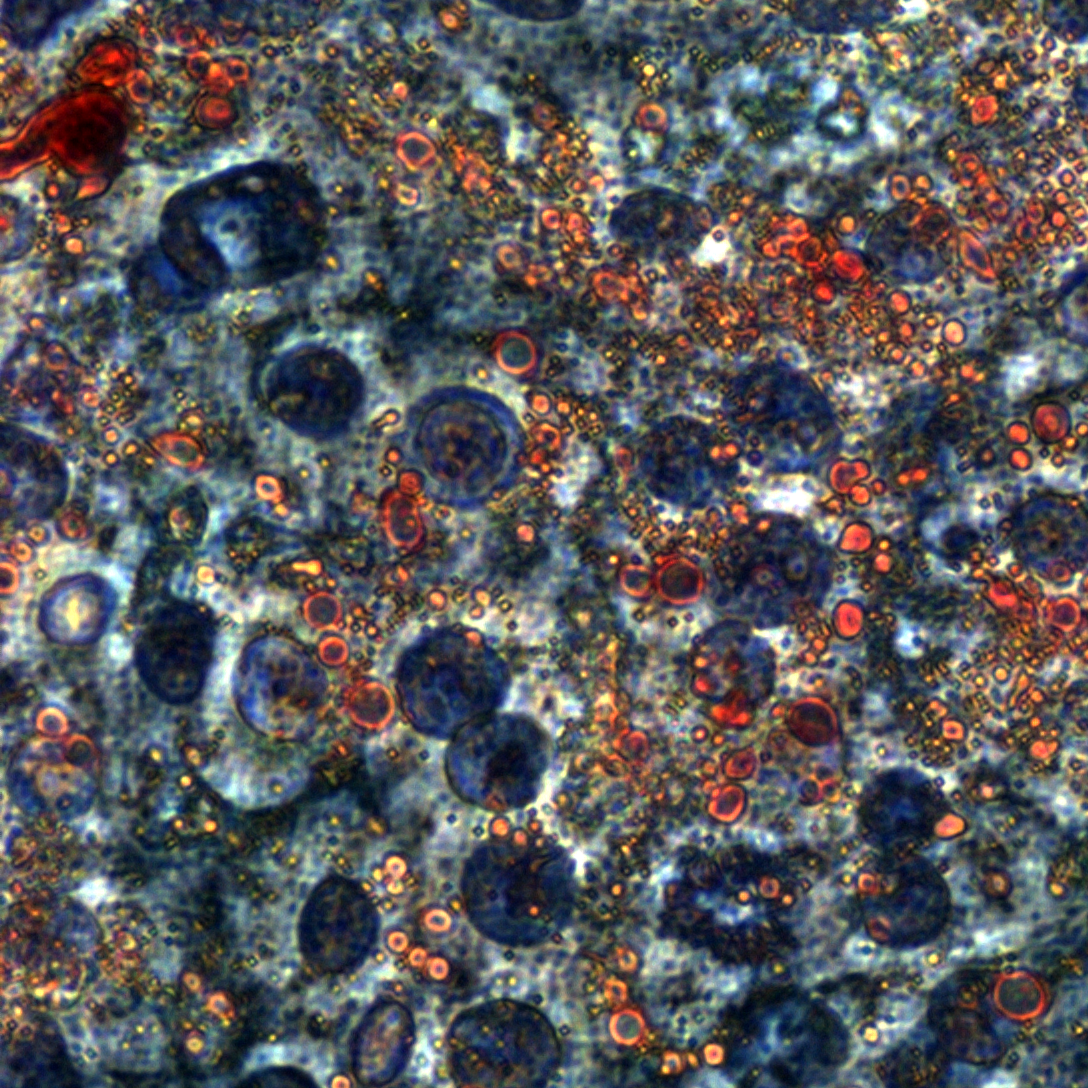

Supplement: Data S1. Raw experimental data generated in this study [file mmc1.zip › All original data/Morphological detection/Oil-red staining/Oil-red staining for Figure 5/NC/NC-3.tif]

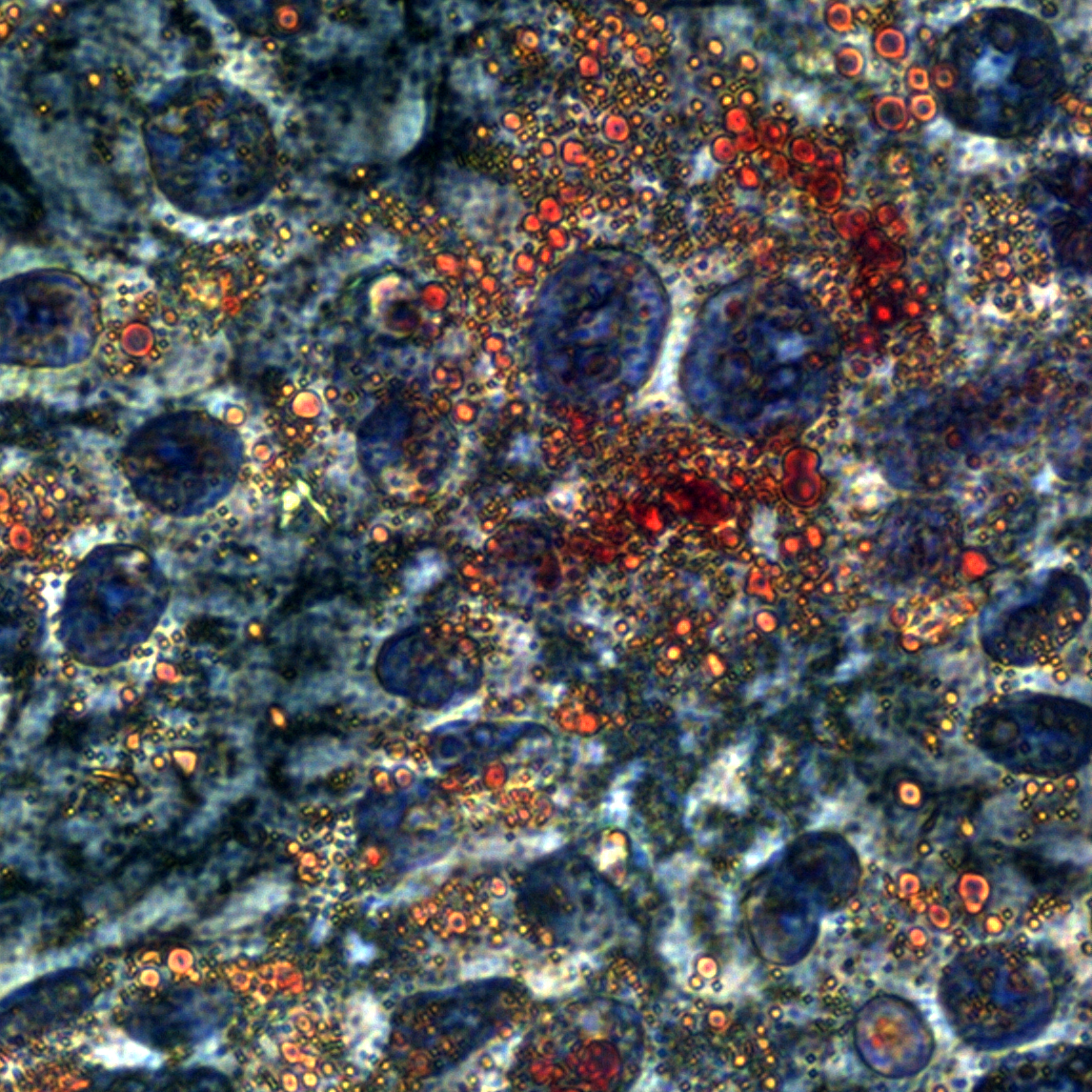

Supplement: Data S1. Raw experimental data generated in this study [file mmc1.zip › All original data/Morphological detection/Oil-red staining/Oil-red staining for Figure 5/NC/NC-4.tif]

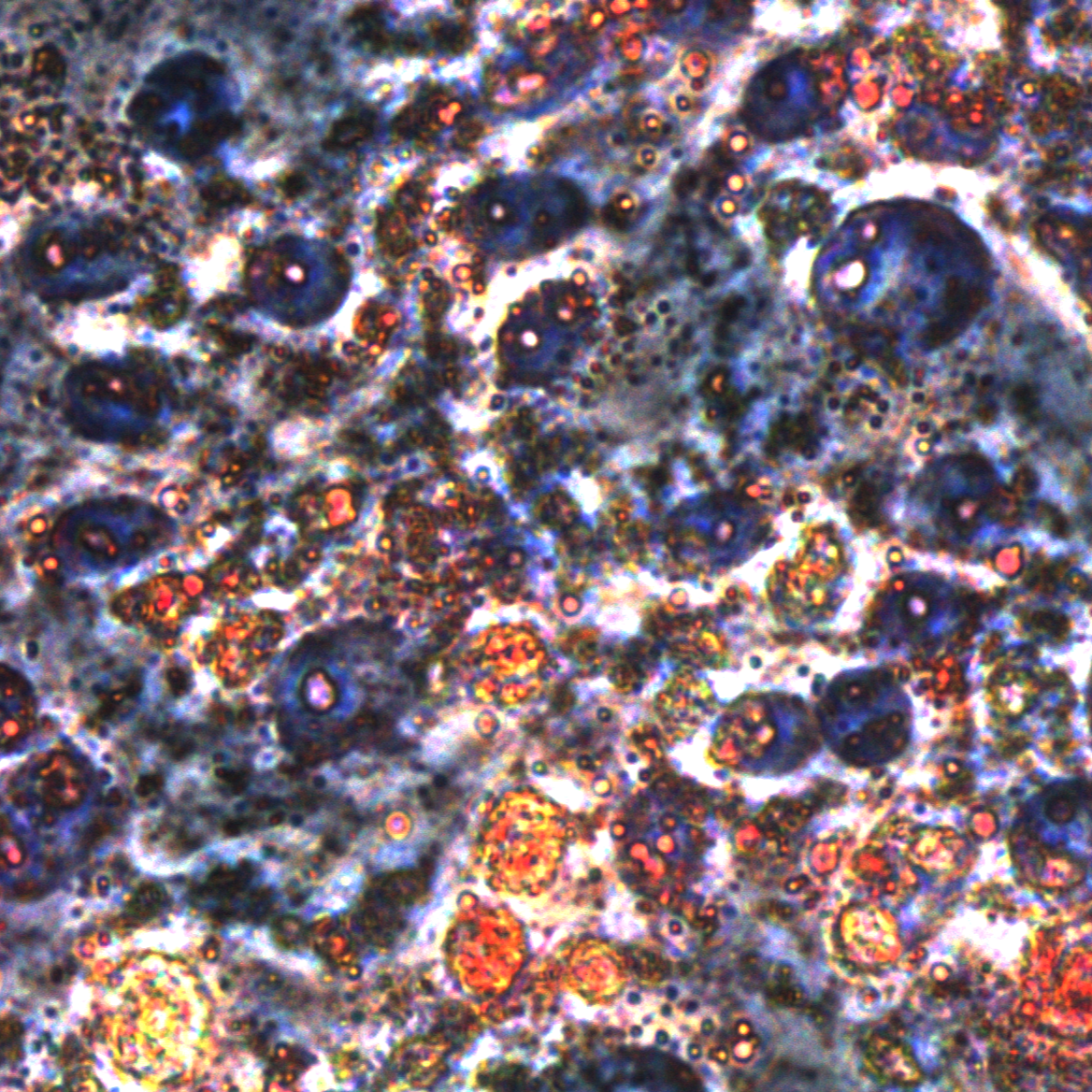

Supplement: Data S1. Raw experimental data generated in this study [file mmc1.zip › All original data/Morphological detection/Oil-red staining/Oil-red staining for Figure 5/NC/NC-5.tif]

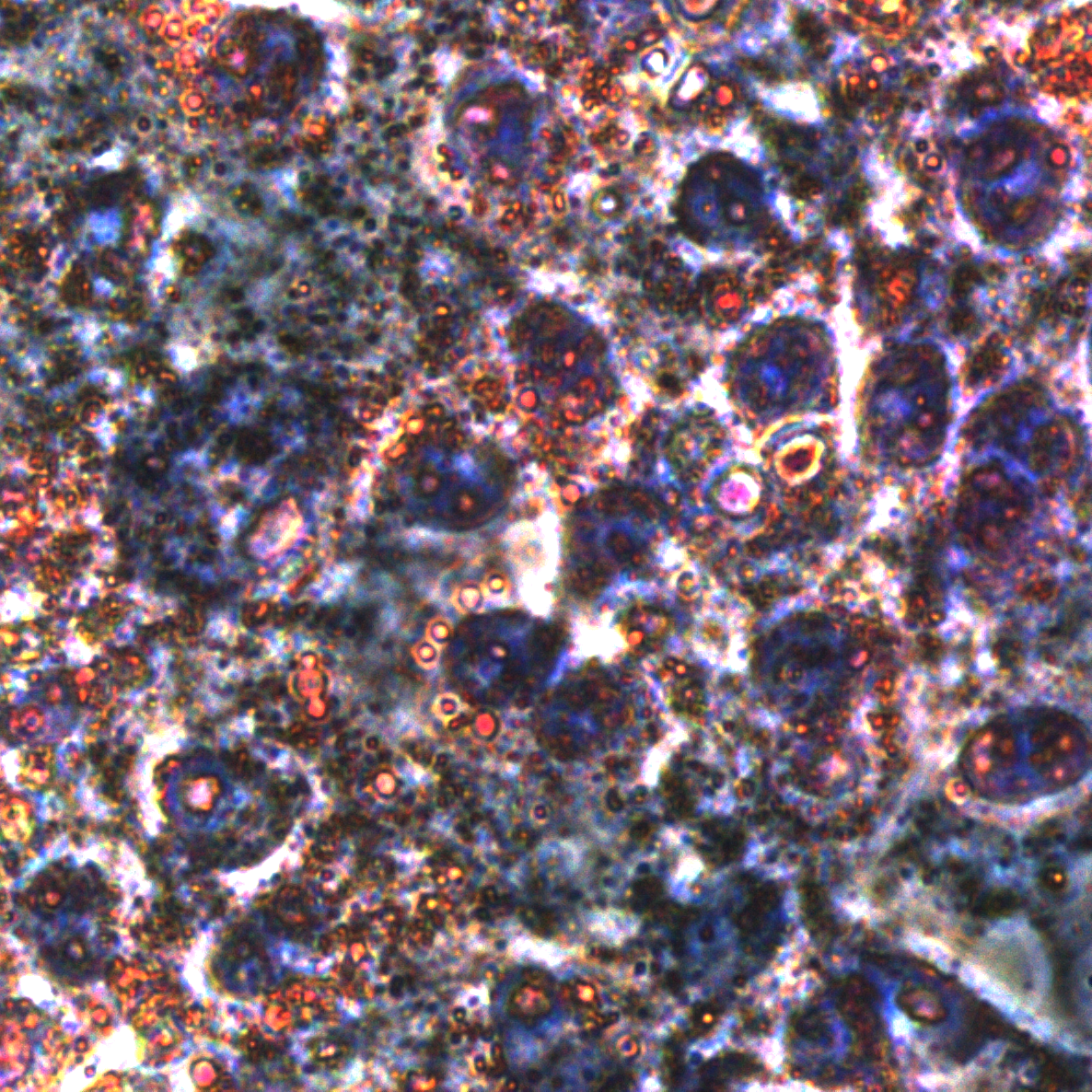

Supplement: Data S1. Raw experimental data generated in this study [file mmc1.zip › All original data/Morphological detection/Oil-red staining/Oil-red staining for Figure 5/NC/NC-6.tif]

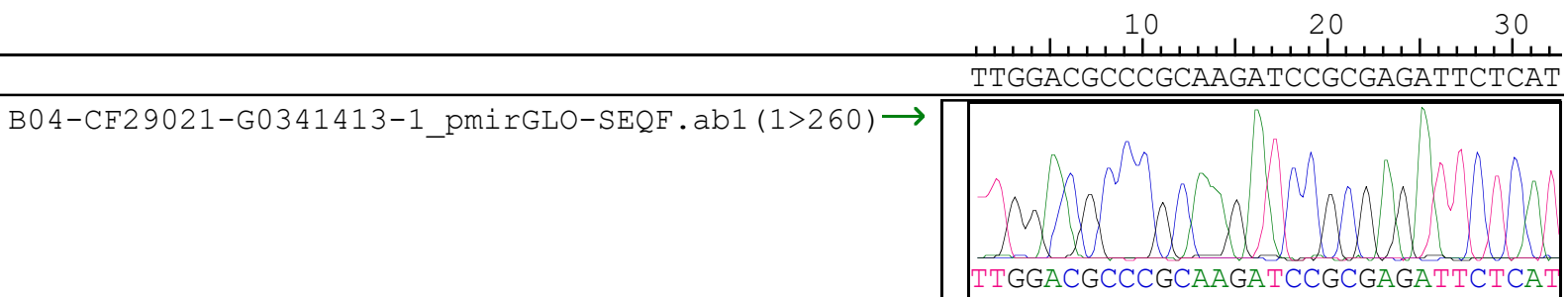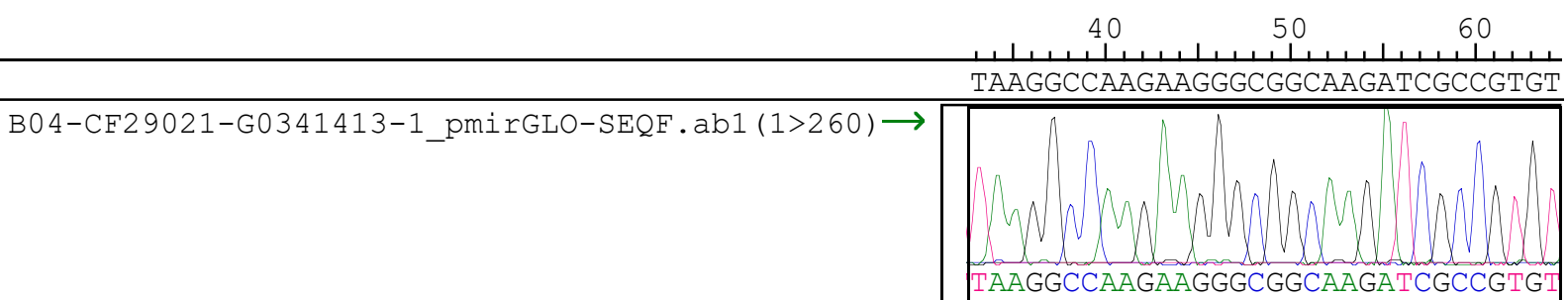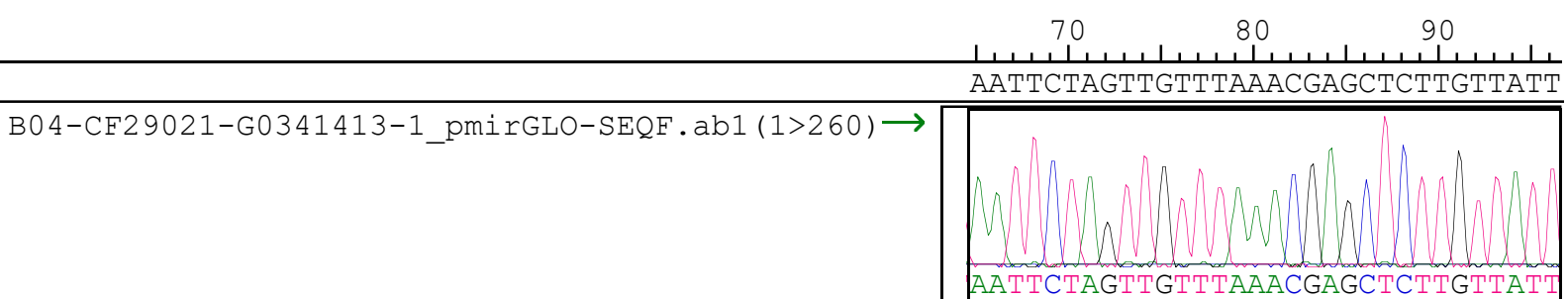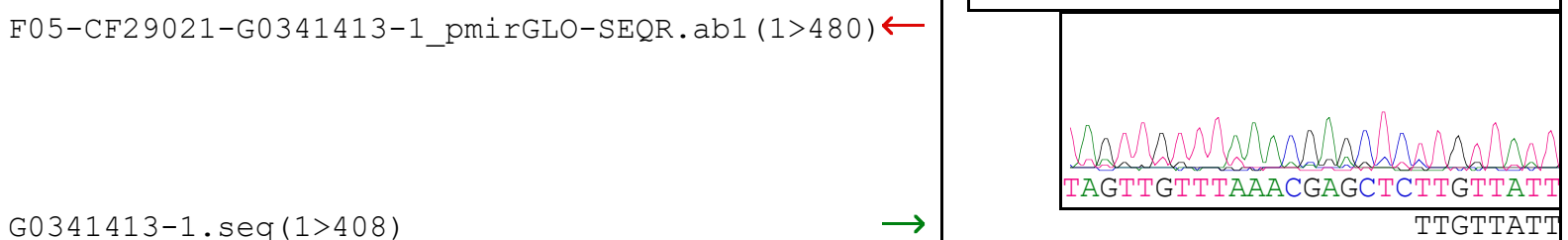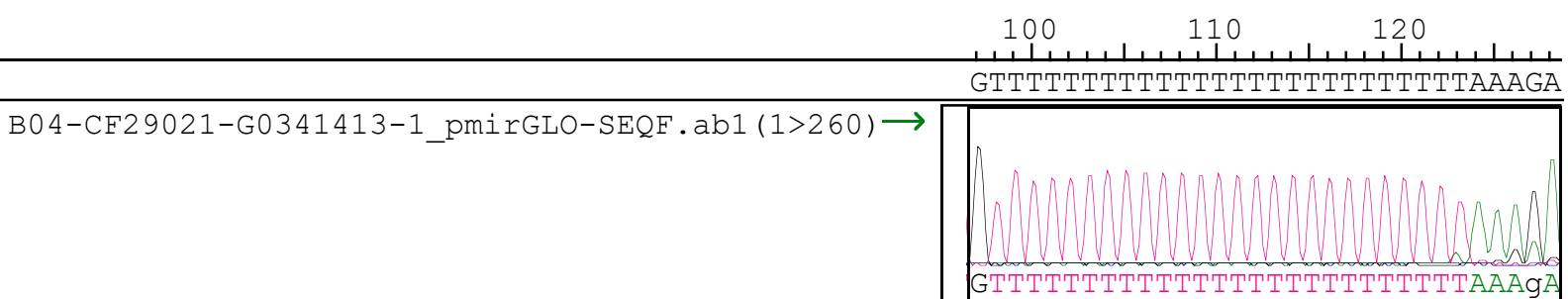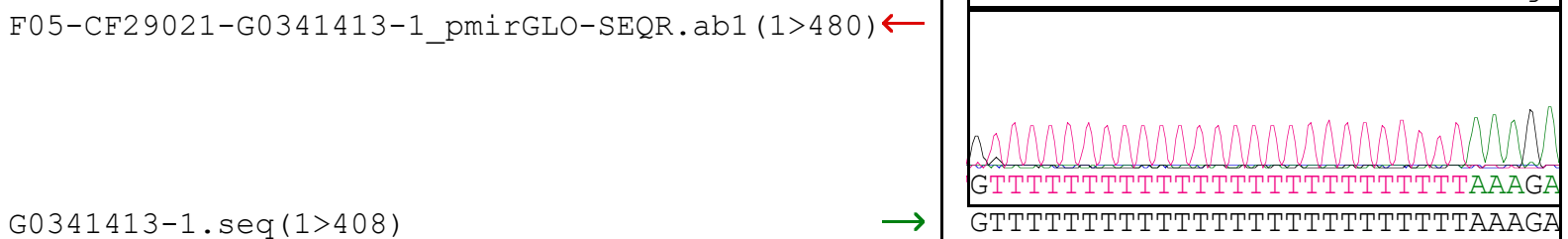

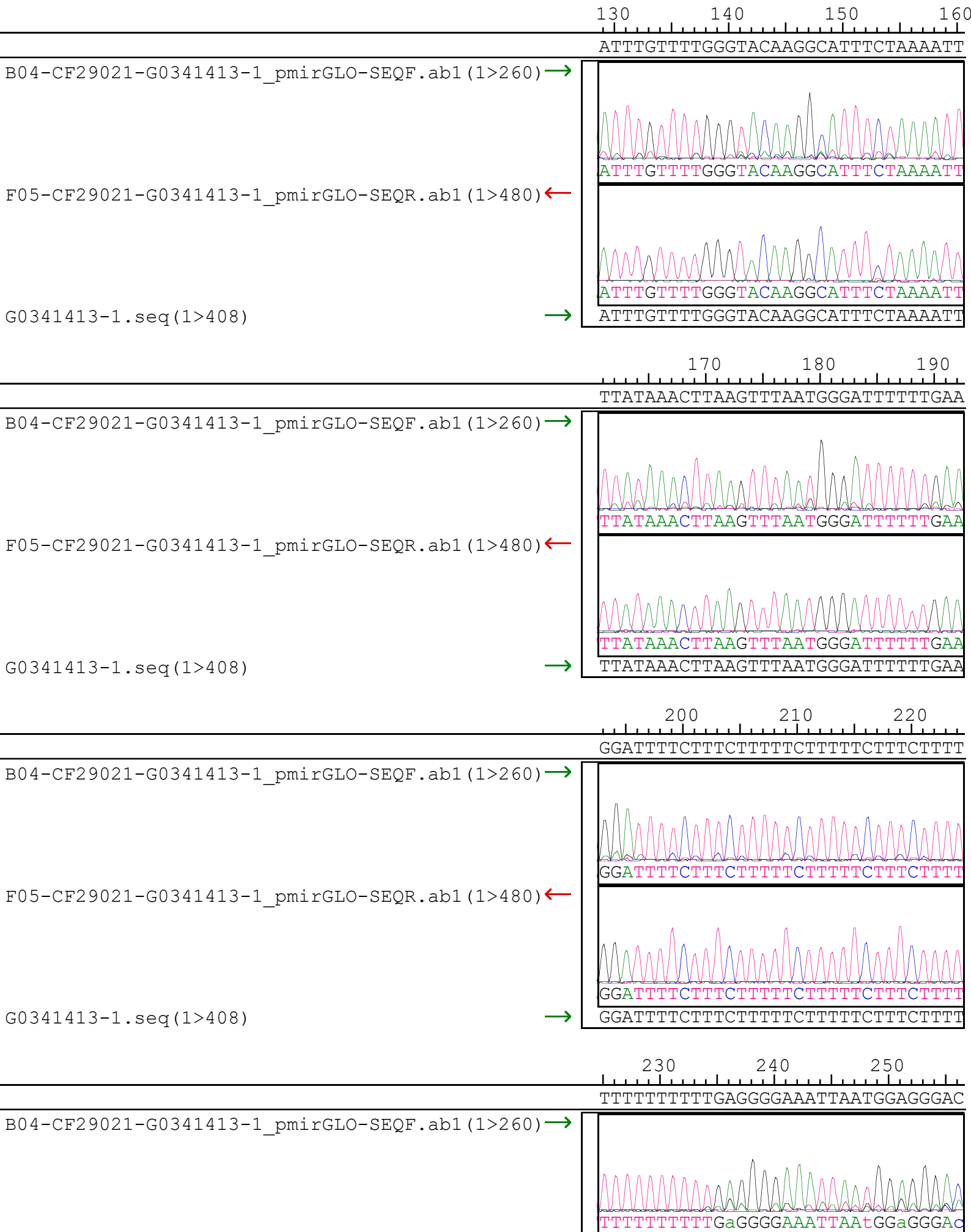

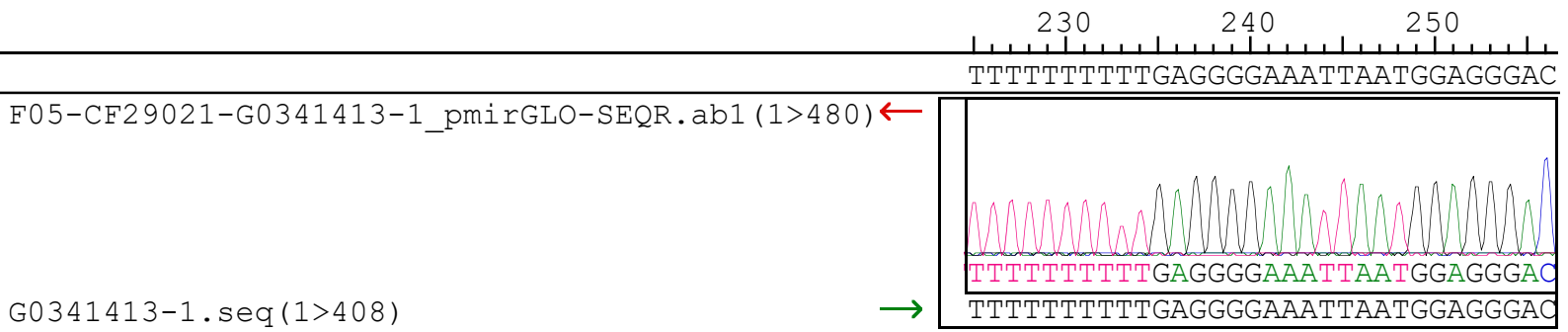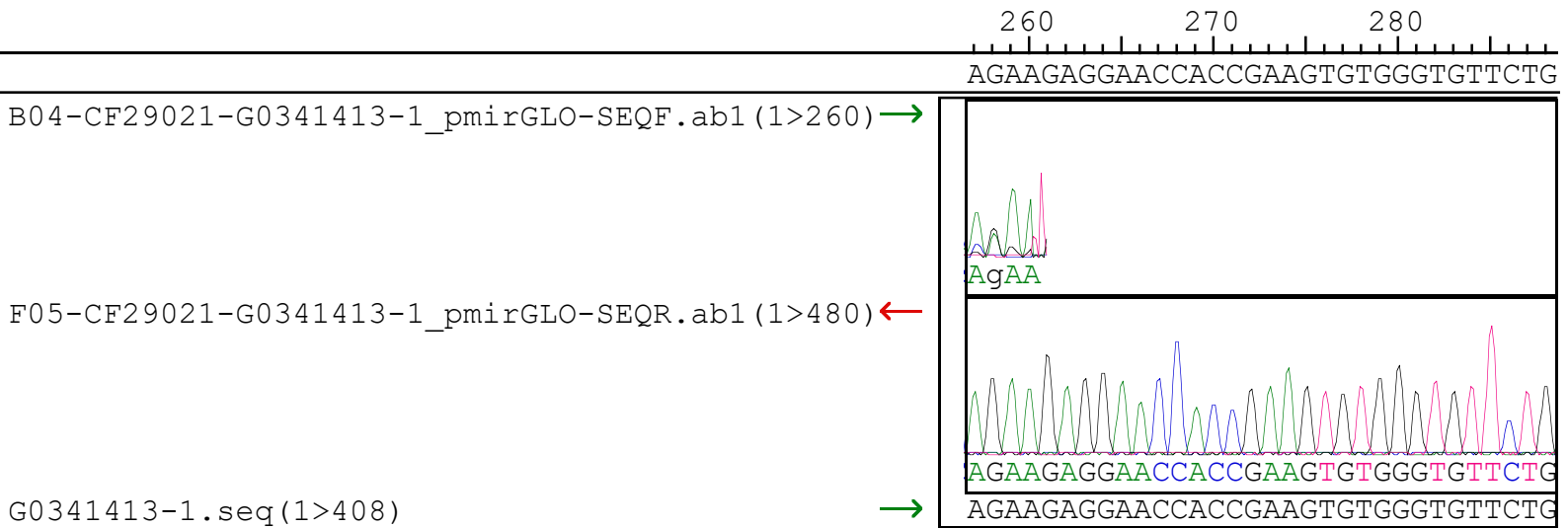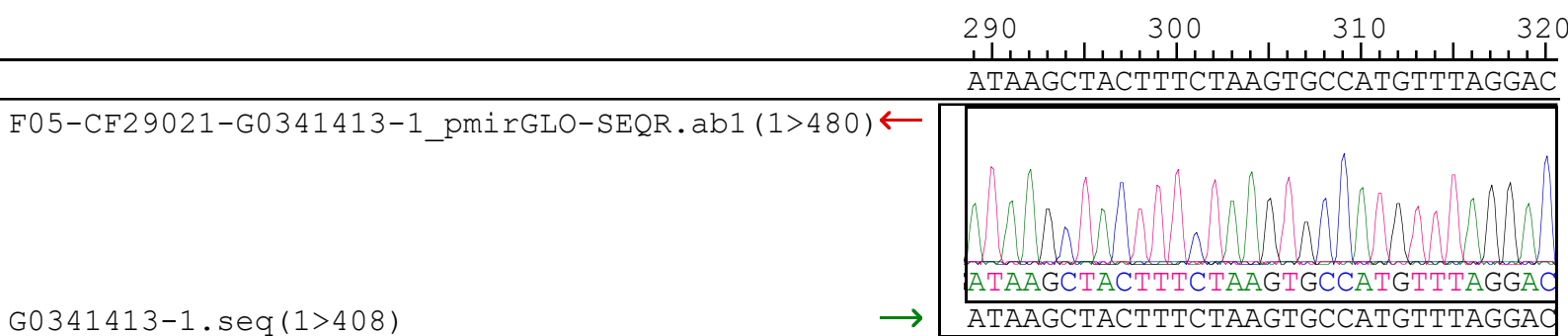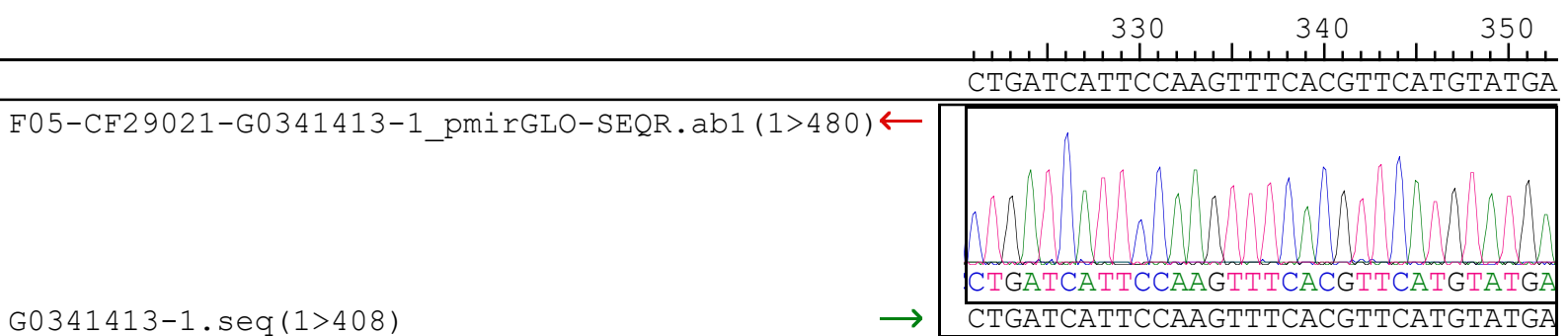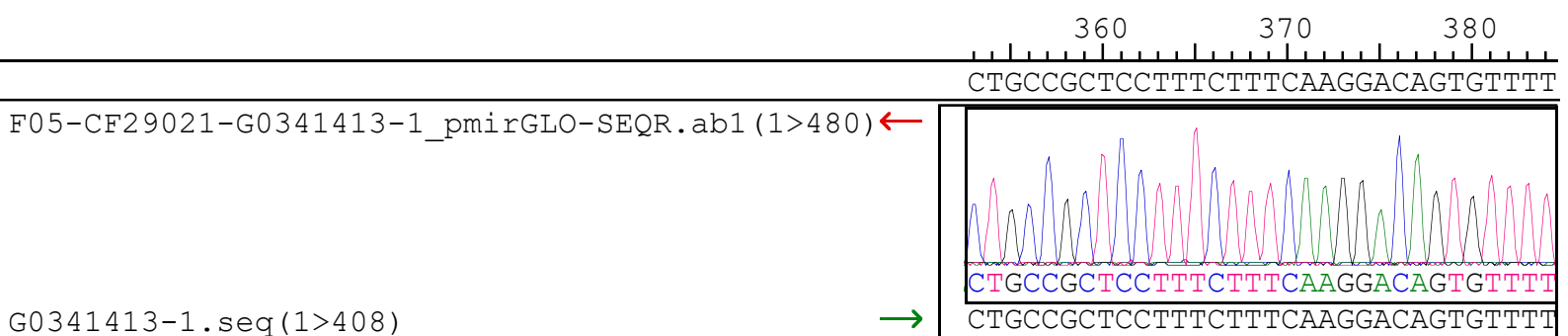

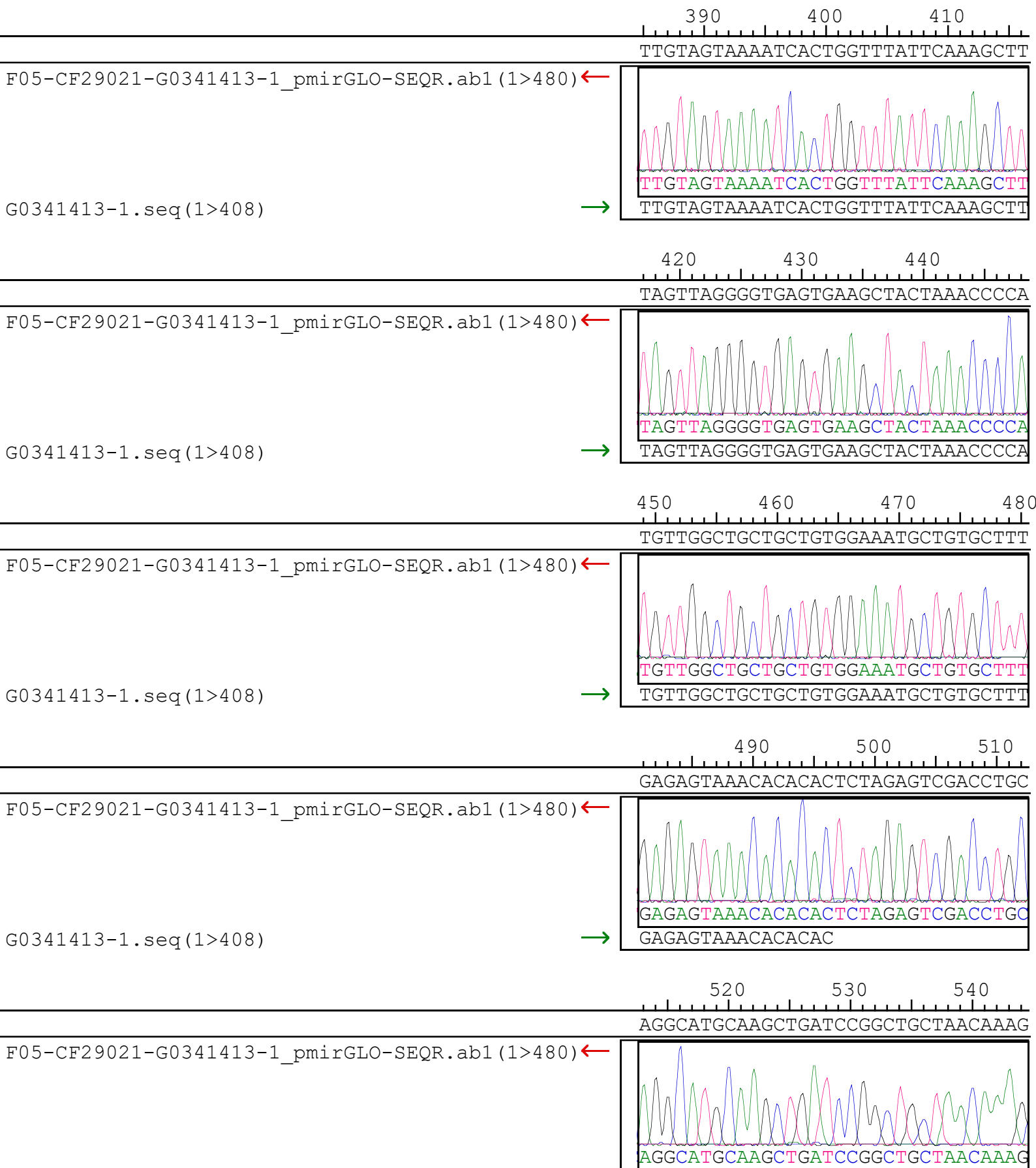

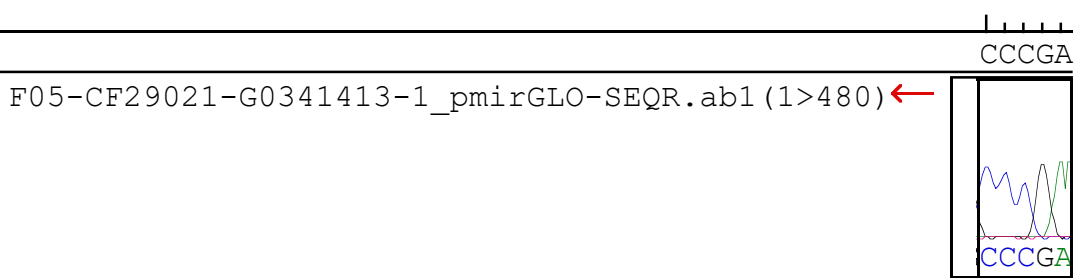

Supplement: Data S1. Raw experimental data generated in this study [file mmc1.zip › All original data/PDCD4 MUT Sequence for New Figure 8/G0341413-1 PDCD4-WT-sense.pdf]

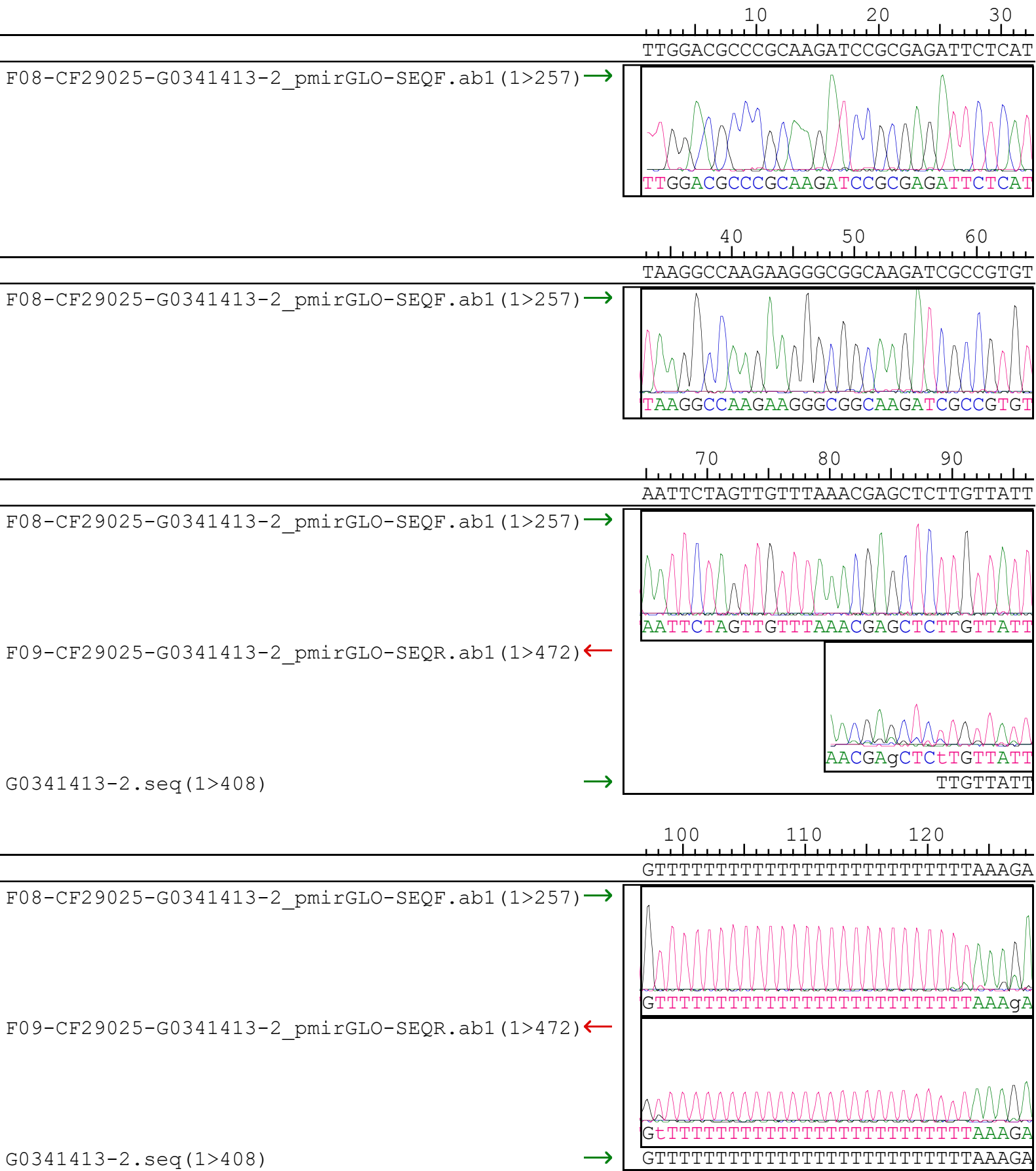

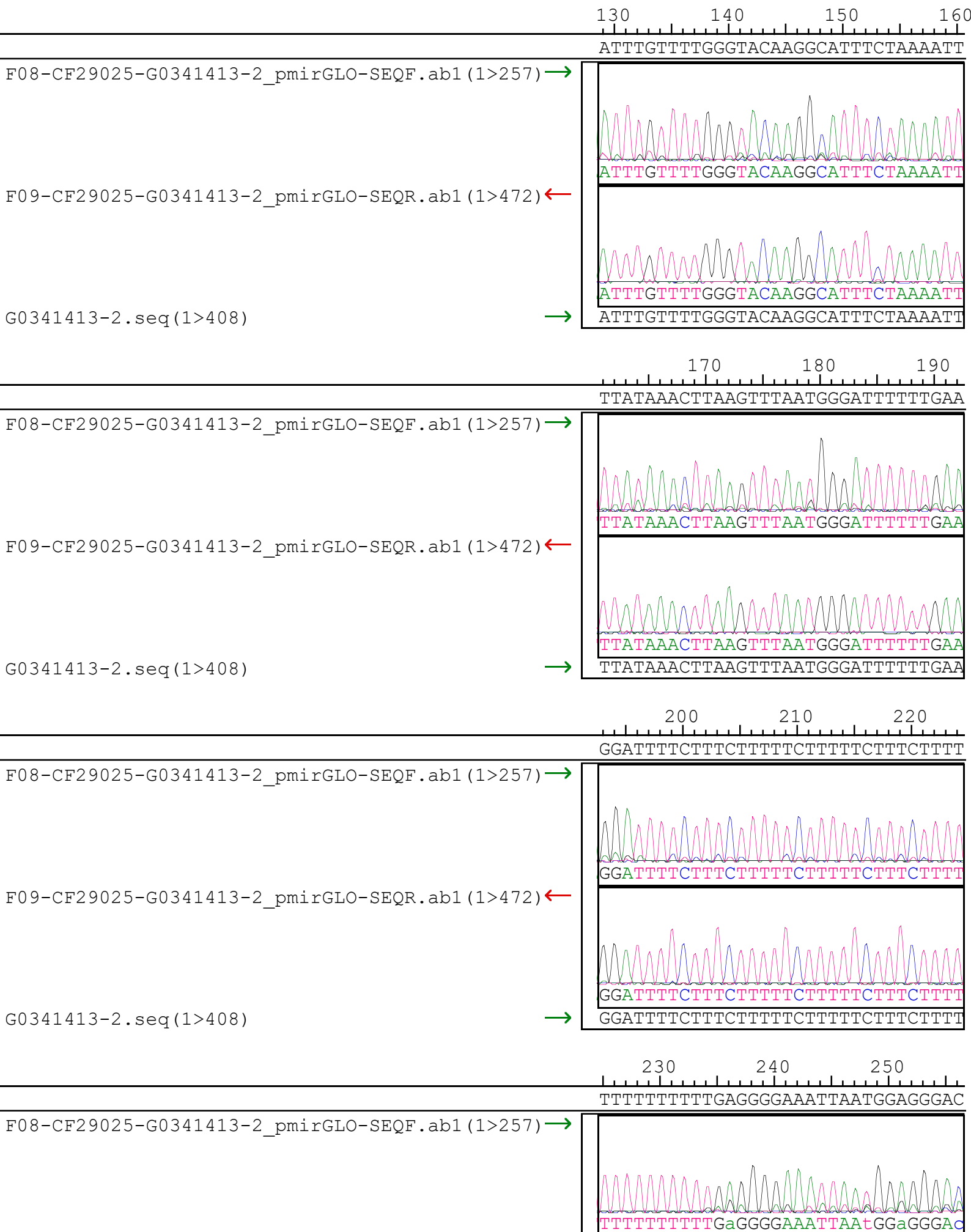

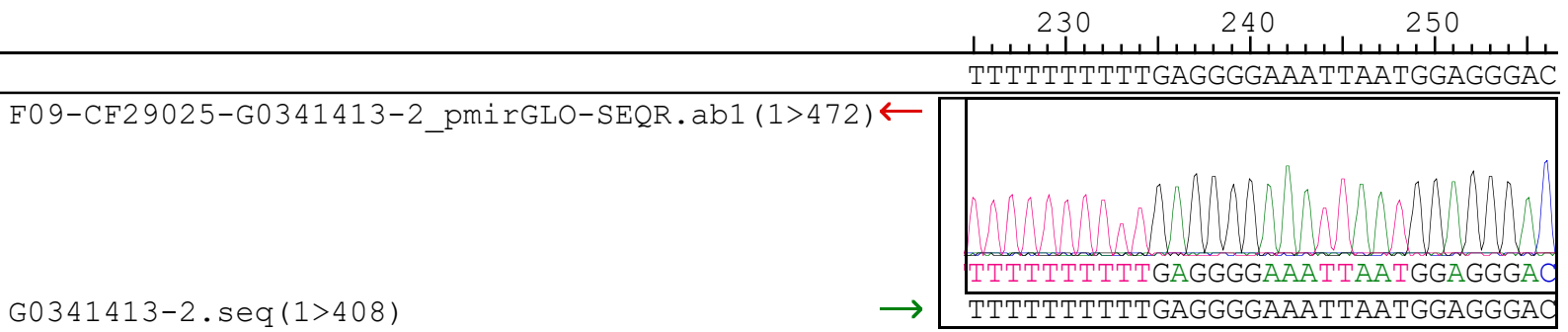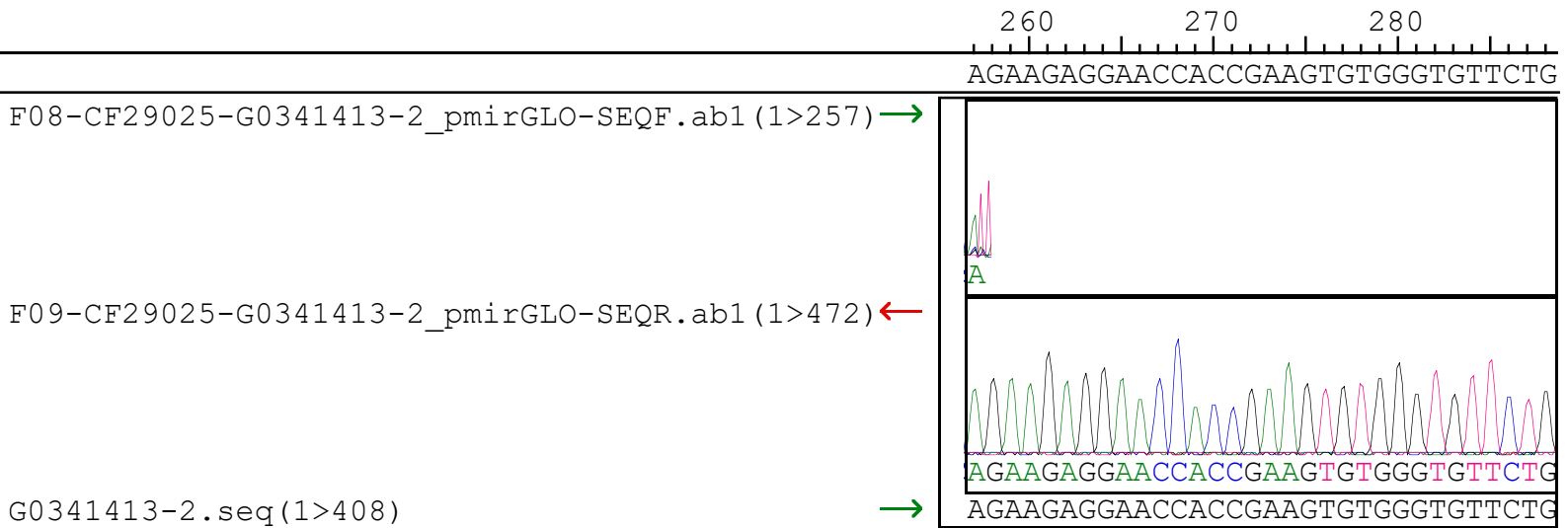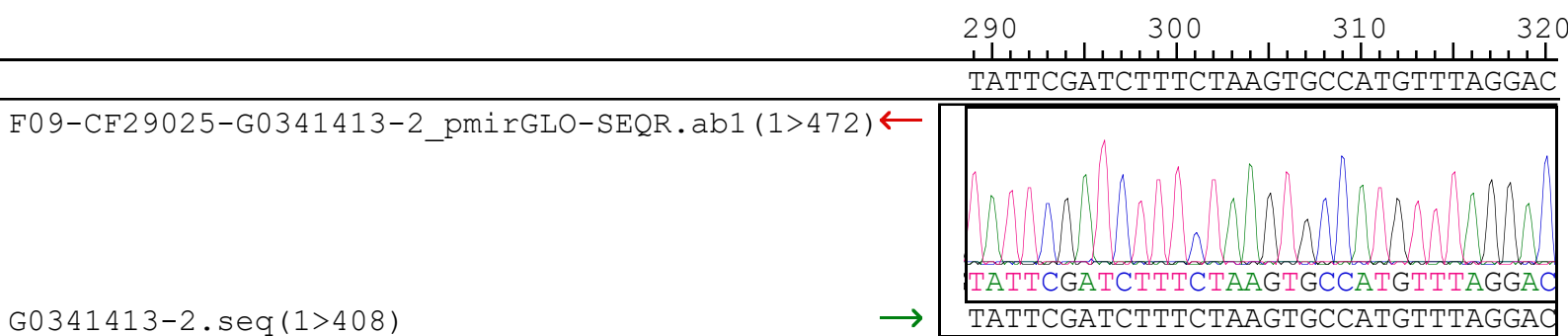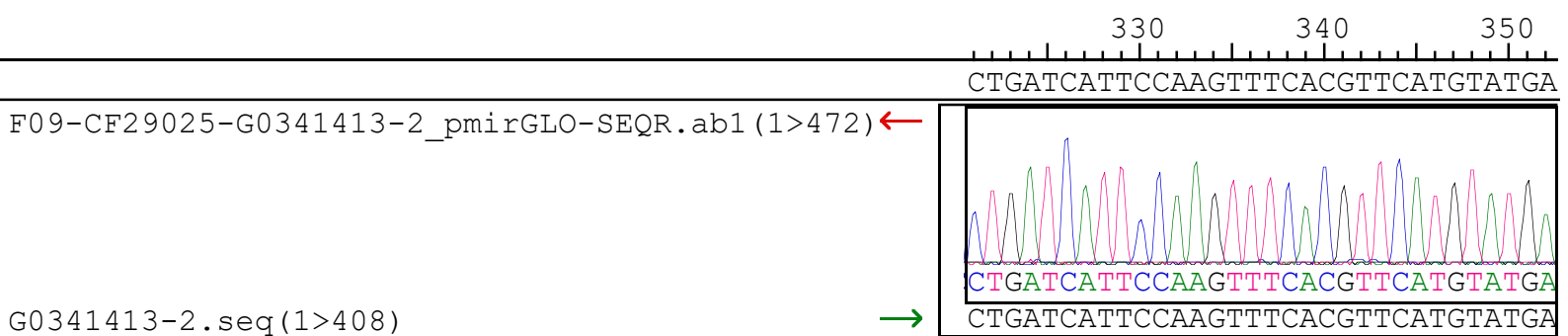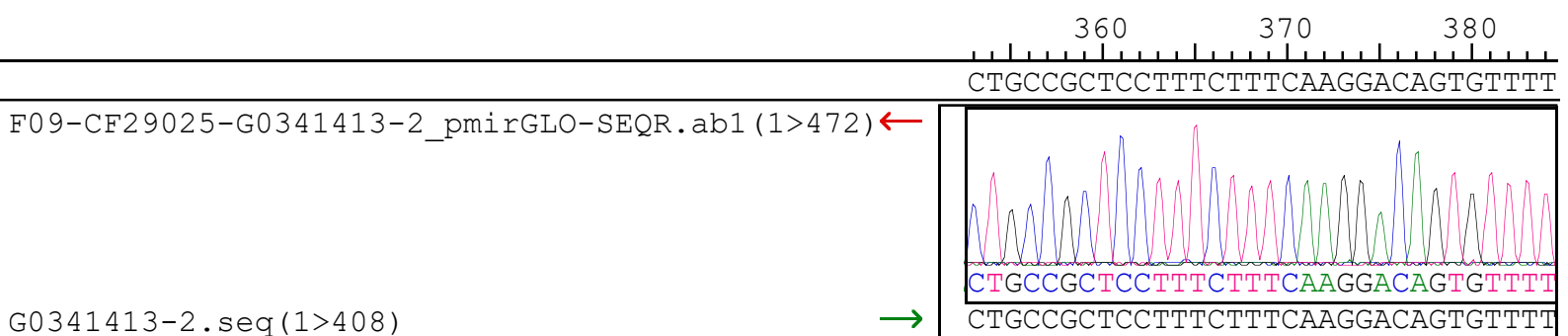

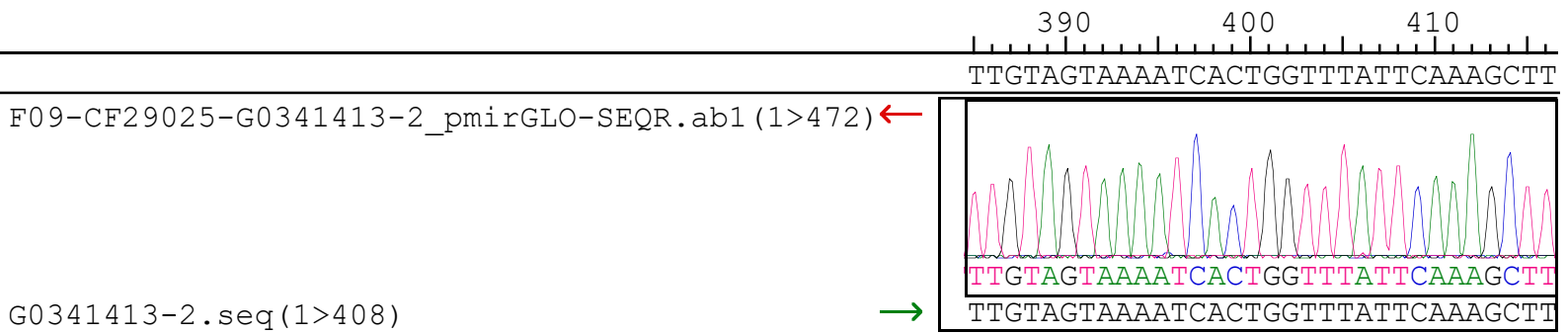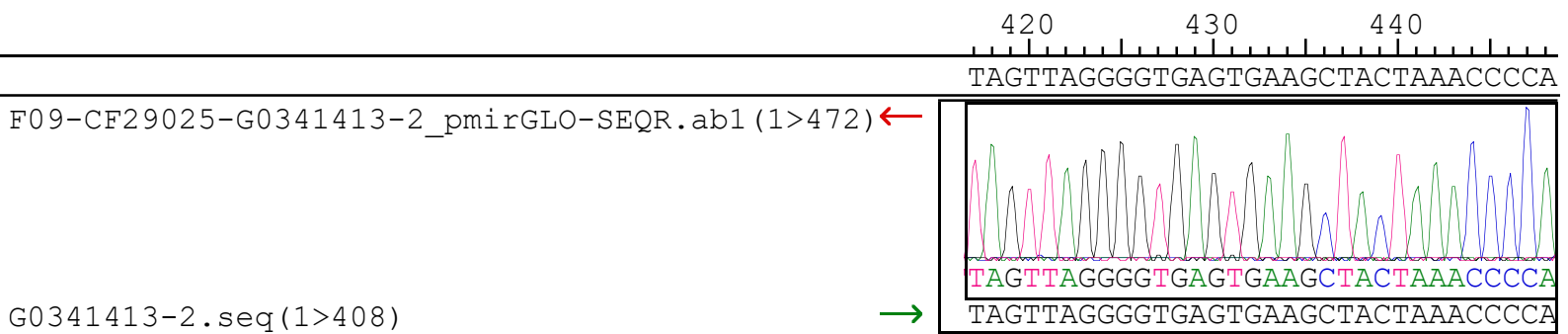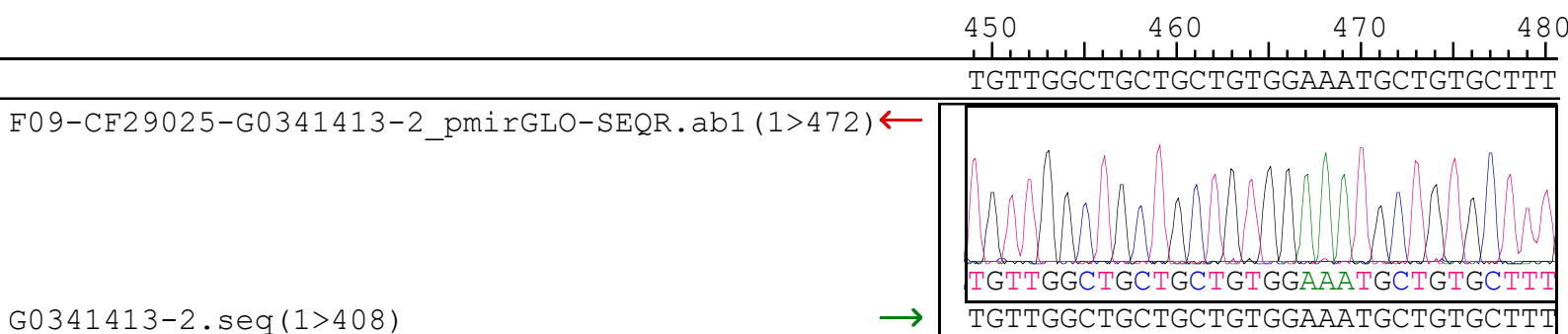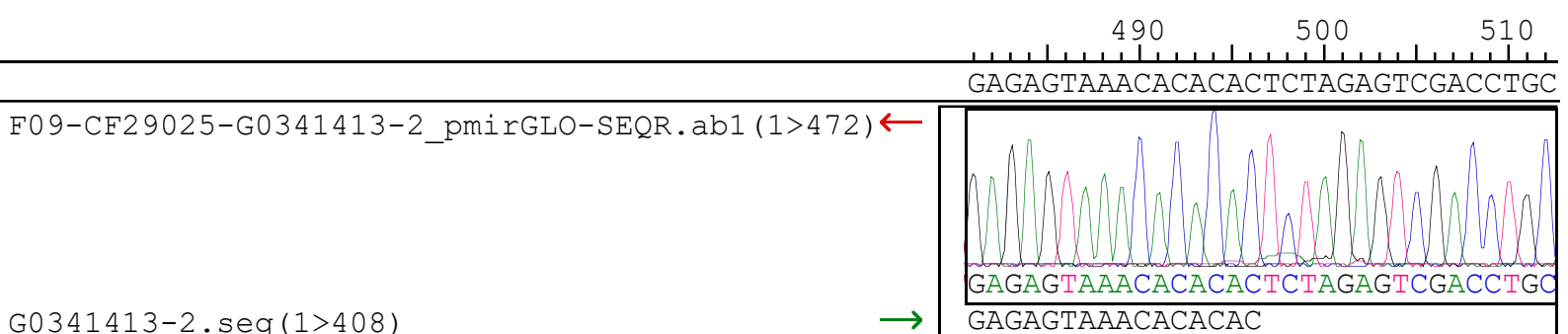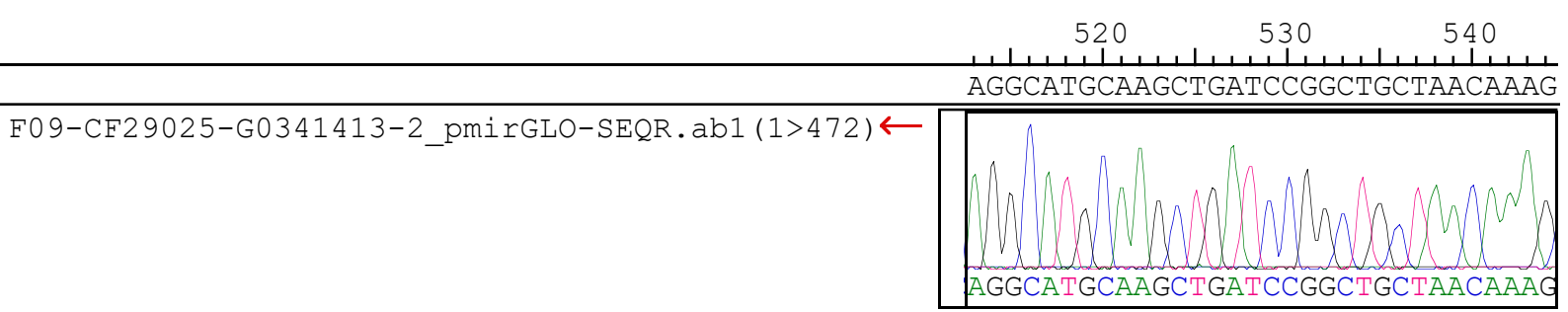

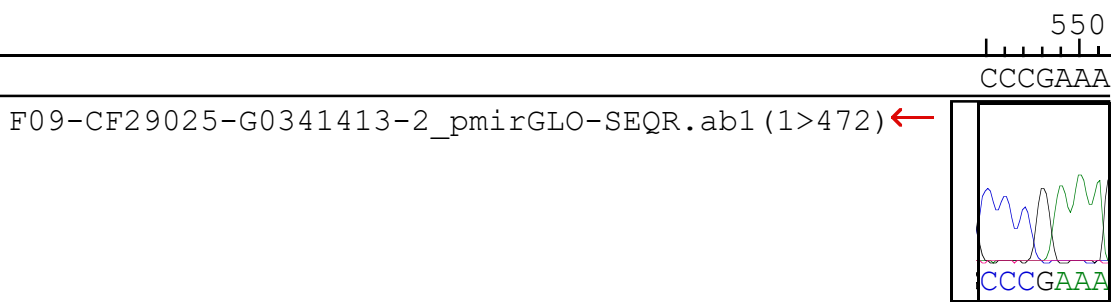

Supplement: Data S1. Raw experimental data generated in this study [file mmc1.zip › All original data/PDCD4 MUT Sequence for New Figure 8/G0341413-2 PDCD4-MUT-sense.pdf]
