# Supplementary figures and images for: A cell-free browning strategy: Exosomal miR-21a-5p from ADSCs targets PDCD4 to reshape adipose metabolism (part 1 of 2)
Source: iScience. 2026 Jul 14;29(8):116765. doi: 10.1016/j.isci.2026.116765 (PMC13382807; doi:10.1016/j.isci.2026.116765)

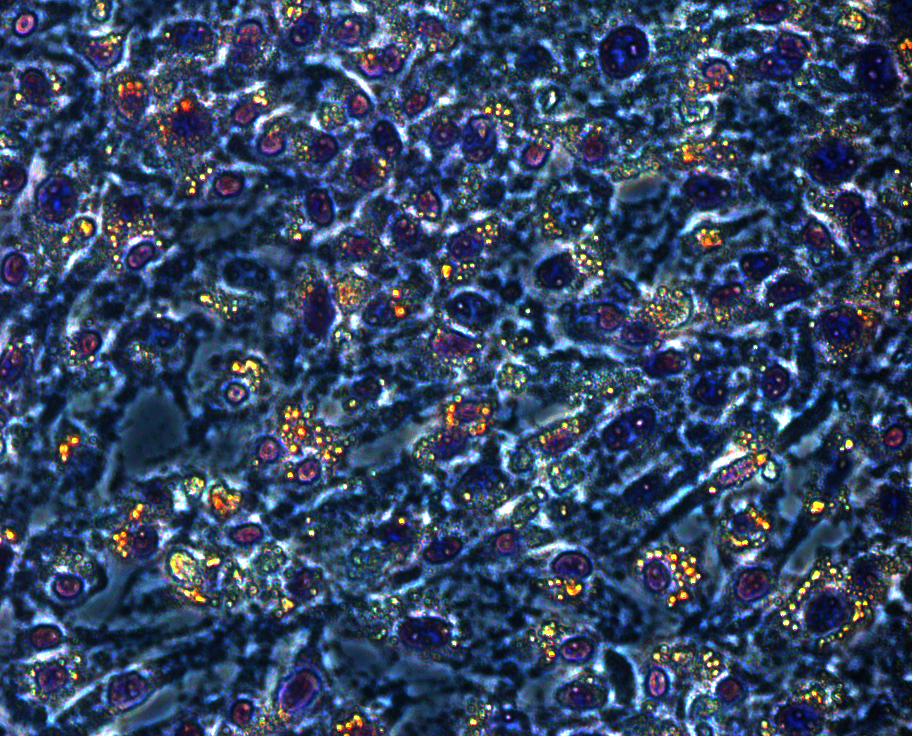

Supplement: Data S1. Raw experimental data generated in this study [file mmc1.zip › All original data/All data for New Figure 6 and New Figure 7/Oil red O staining for New Figure 6 and New Figure 7/oePDCD4/miR-21a-5p mimic.tif]

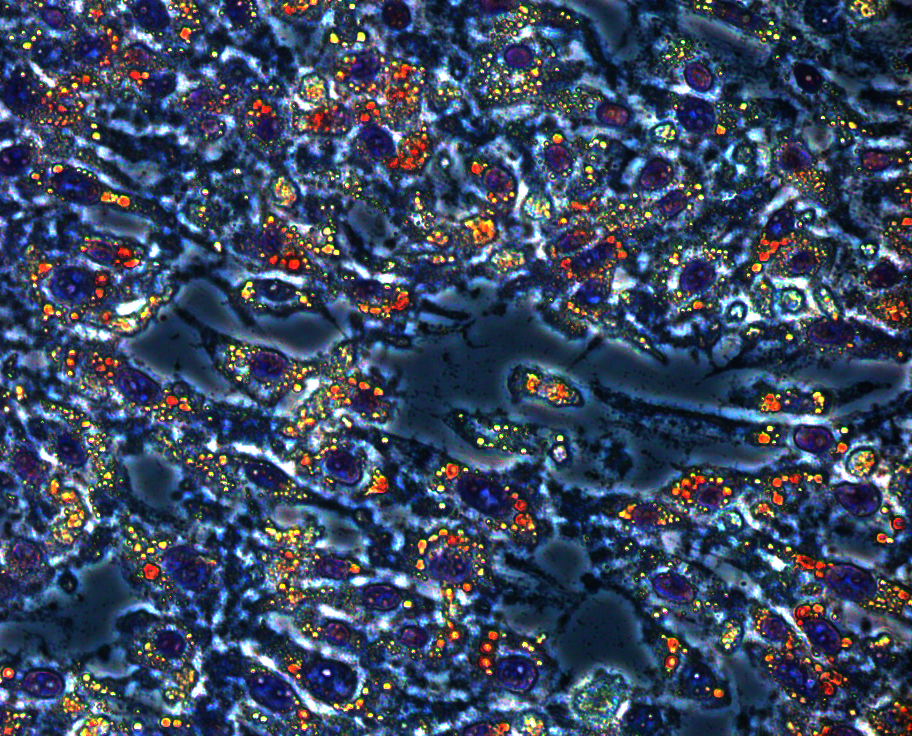

Supplement: Data S1. Raw experimental data generated in this study [file mmc1.zip › All original data/All data for New Figure 6 and New Figure 7/Oil red O staining for New Figure 6 and New Figure 7/oePDCD4/NC.tif]

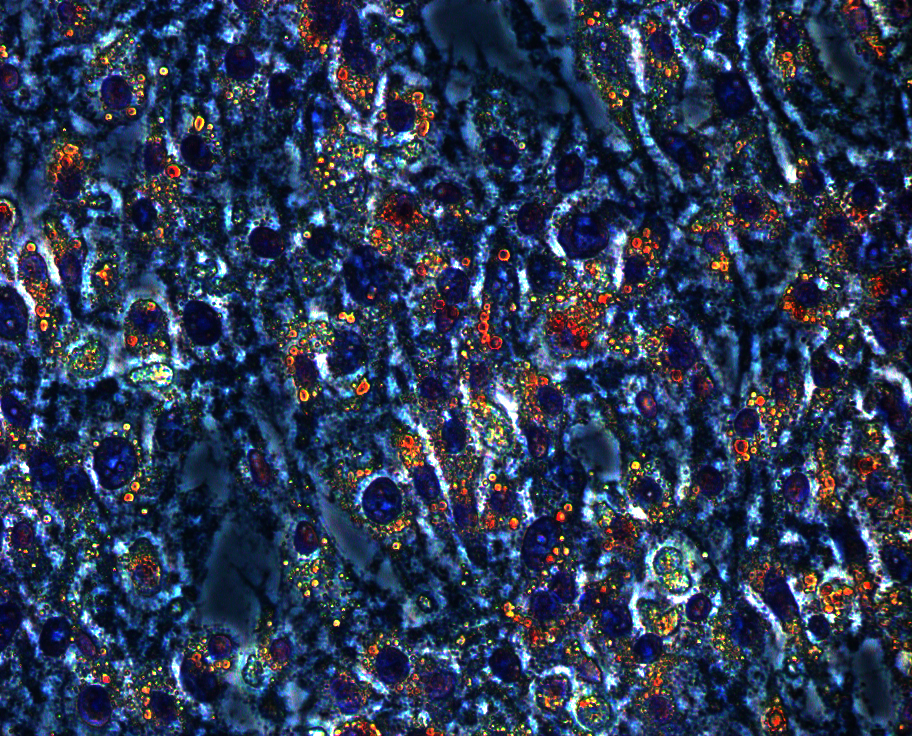

Supplement: Data S1. Raw experimental data generated in this study [file mmc1.zip › All original data/All data for New Figure 6 and New Figure 7/Oil red O staining for New Figure 6 and New Figure 7/oePDCD4/oePDCD4.tif]

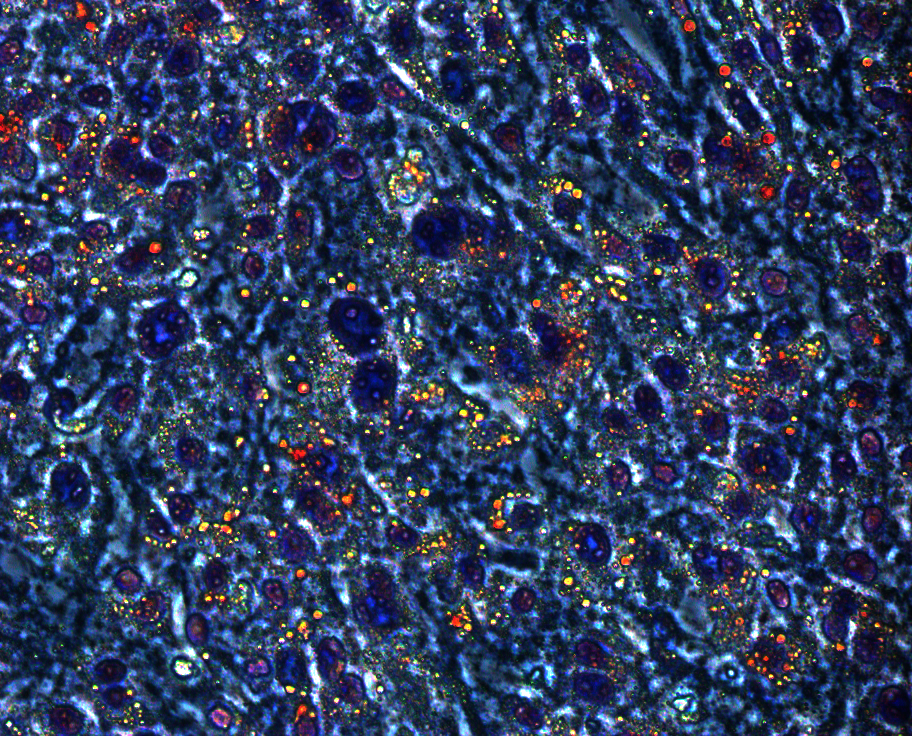

Supplement: Data S1. Raw experimental data generated in this study [file mmc1.zip › All original data/All data for New Figure 6 and New Figure 7/Oil red O staining for New Figure 6 and New Figure 7/oePDCD4/oePDCD4+miR-21a-5p mimic.tif]

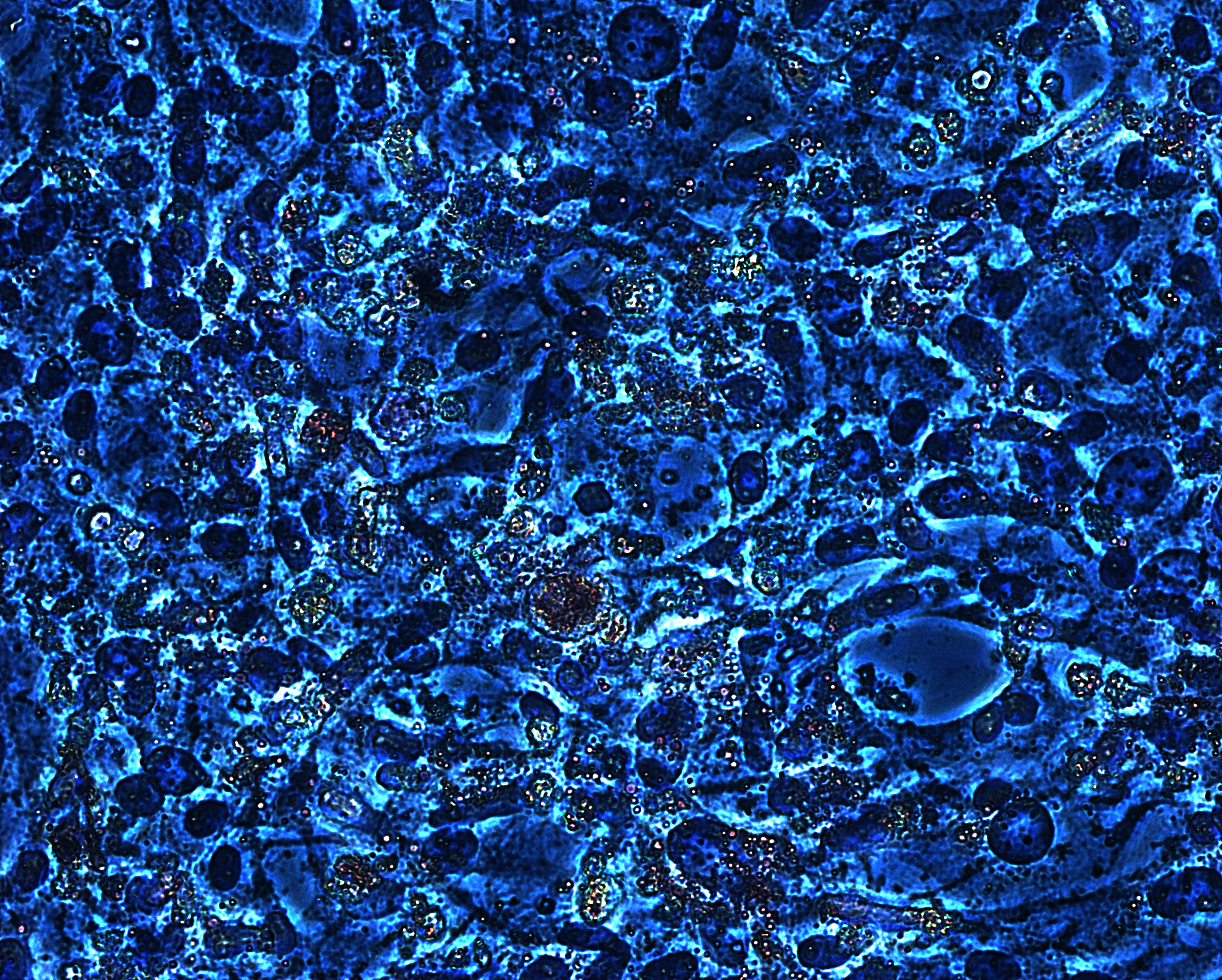

Supplement: Data S1. Raw experimental data generated in this study [file mmc1.zip › All original data/All data for New Figure 6 and New Figure 7/Oil red O staining for New Figure 6 and New Figure 7/siPDCD4/miR-21a-5p mimic.tif]

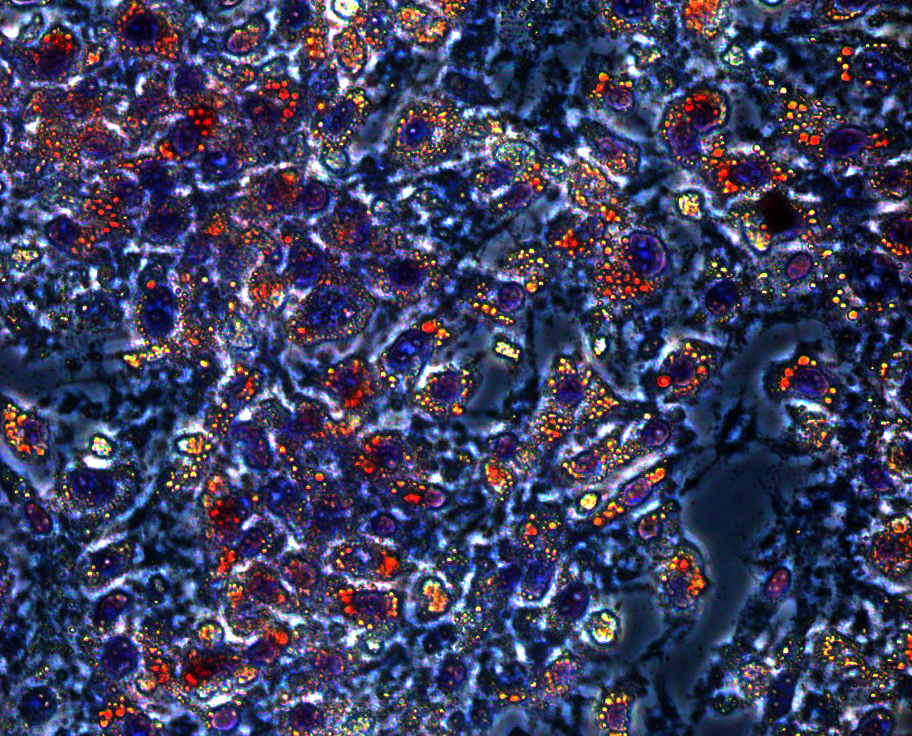

Supplement: Data S1. Raw experimental data generated in this study [file mmc1.zip › All original data/All data for New Figure 6 and New Figure 7/Oil red O staining for New Figure 6 and New Figure 7/siPDCD4/NC.tif]

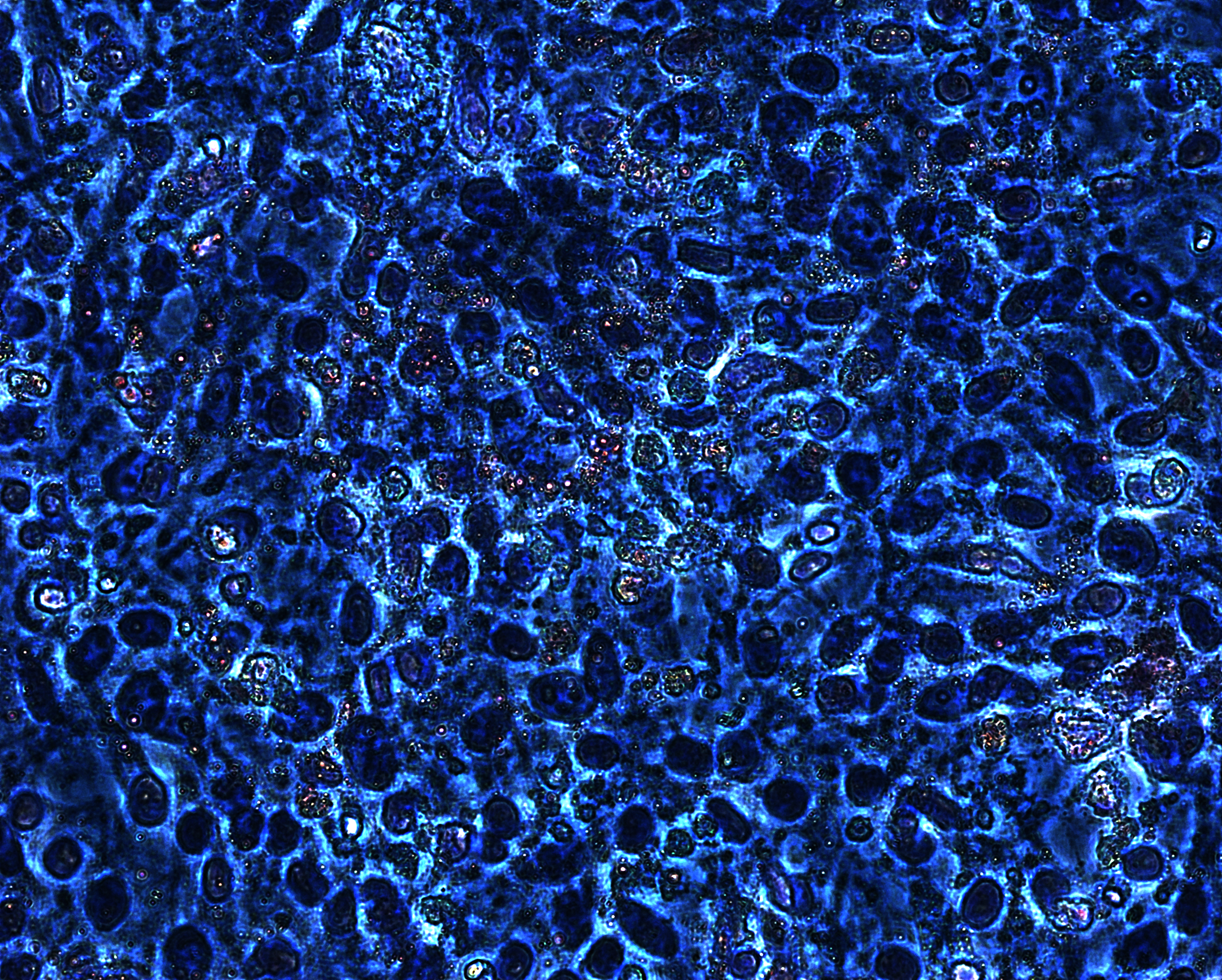

Supplement: Data S1. Raw experimental data generated in this study [file mmc1.zip › All original data/All data for New Figure 6 and New Figure 7/Oil red O staining for New Figure 6 and New Figure 7/siPDCD4/siPDCD4.tif]

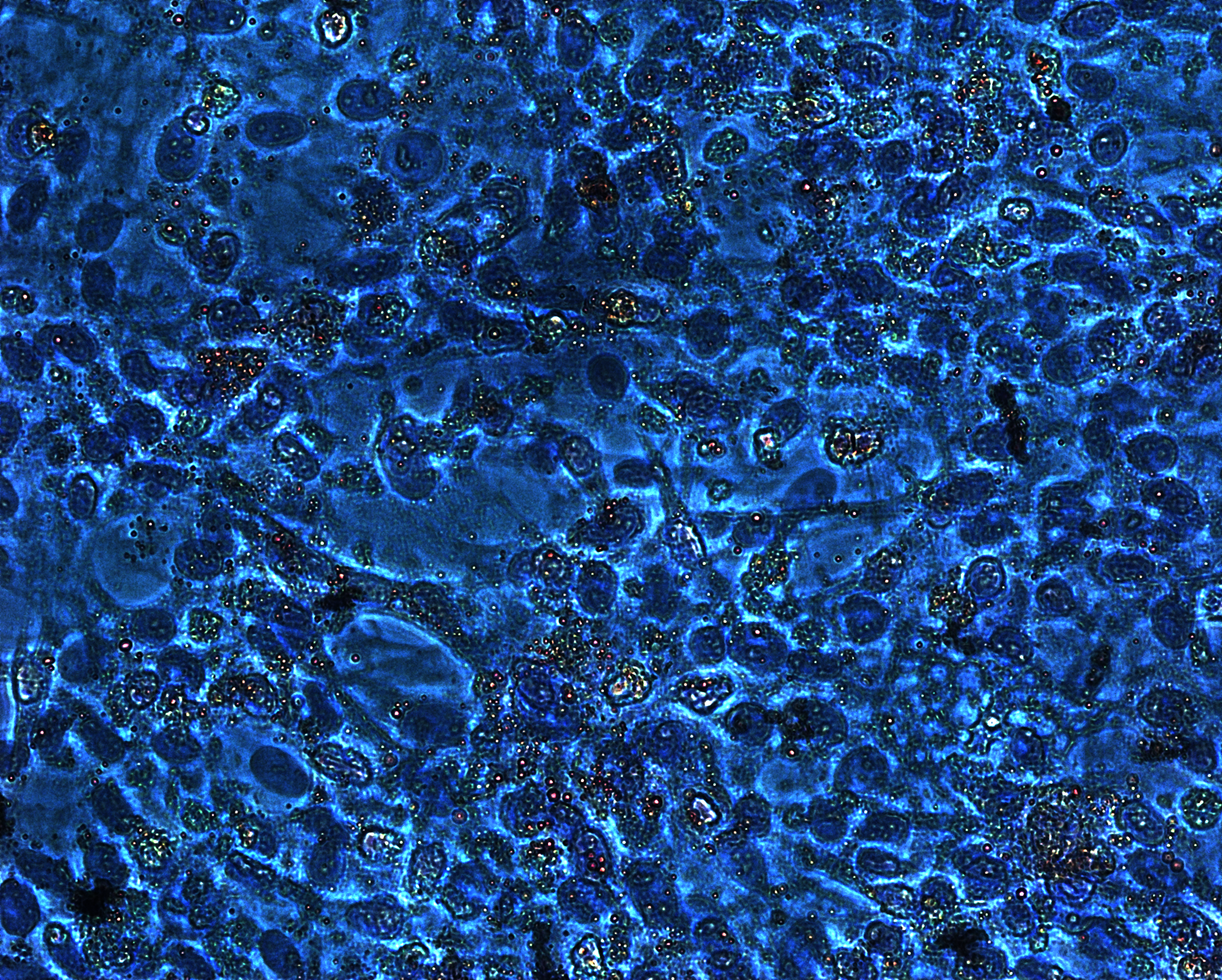

Supplement: Data S1. Raw experimental data generated in this study [file mmc1.zip › All original data/All data for New Figure 6 and New Figure 7/Oil red O staining for New Figure 6 and New Figure 7/siPDCD4/siPDCD4+miR-21a-5p mimic.tif]

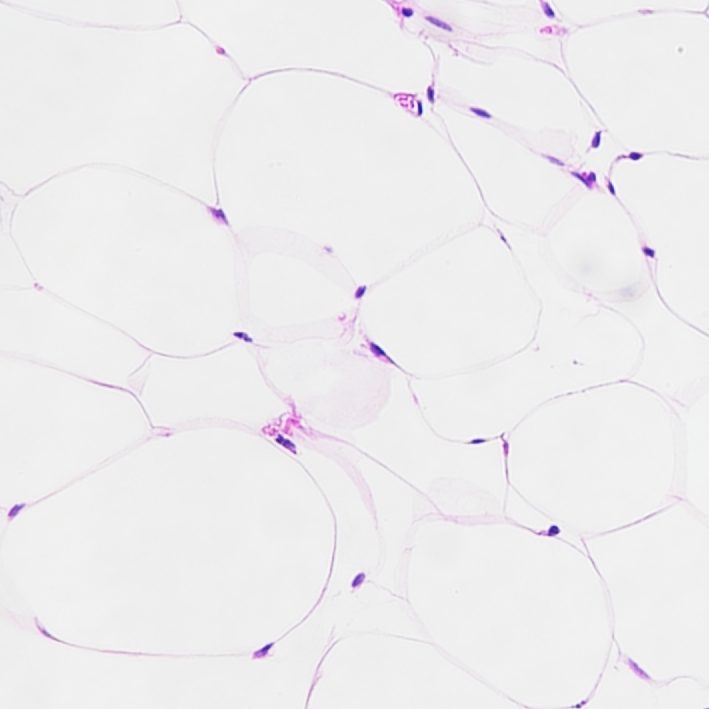

Supplement: Data S1. Raw experimental data generated in this study [file mmc1.zip › All original data/Morphological detection/HE for Figure 2/HFD/HFD-1 used for manuscript.tif]

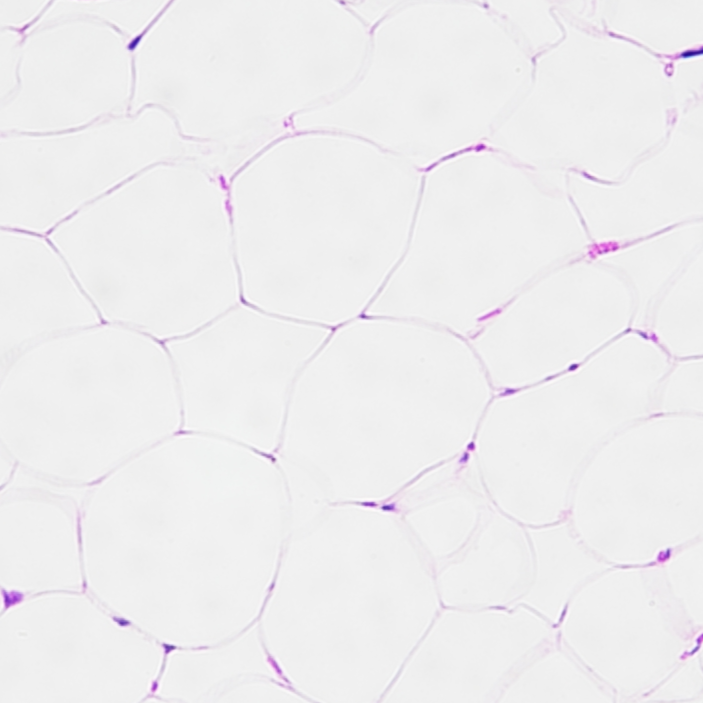

Supplement: Data S1. Raw experimental data generated in this study [file mmc1.zip › All original data/Morphological detection/HE for Figure 2/HFD/HFD-2.tif]

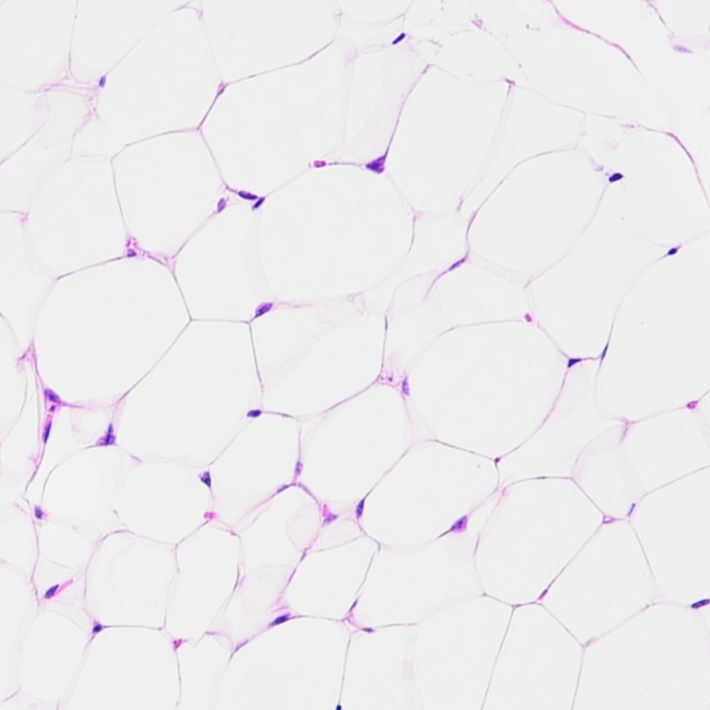

Supplement: Data S1. Raw experimental data generated in this study [file mmc1.zip › All original data/Morphological detection/HE for Figure 2/HFD/HFD-3.tif]

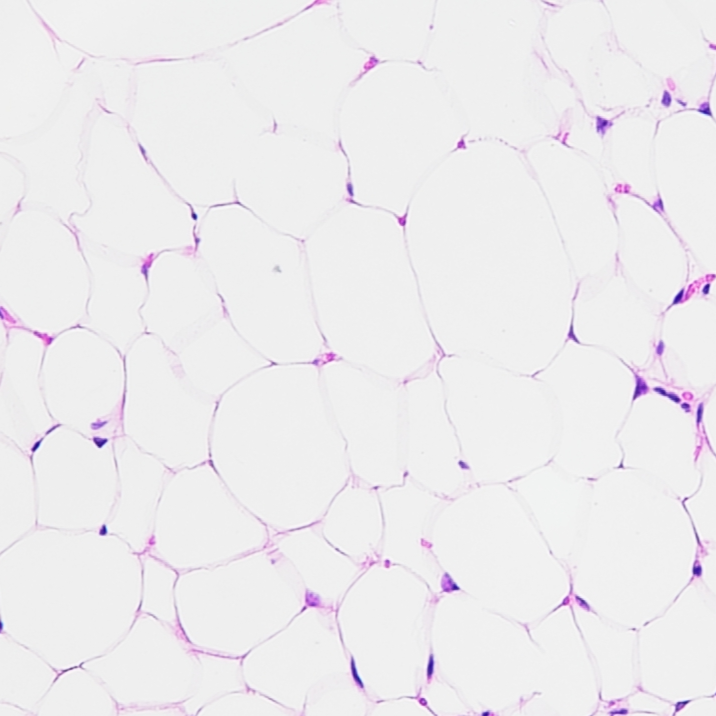

Supplement: Data S1. Raw experimental data generated in this study [file mmc1.zip › All original data/Morphological detection/HE for Figure 2/HFD/HFD-4.tif]

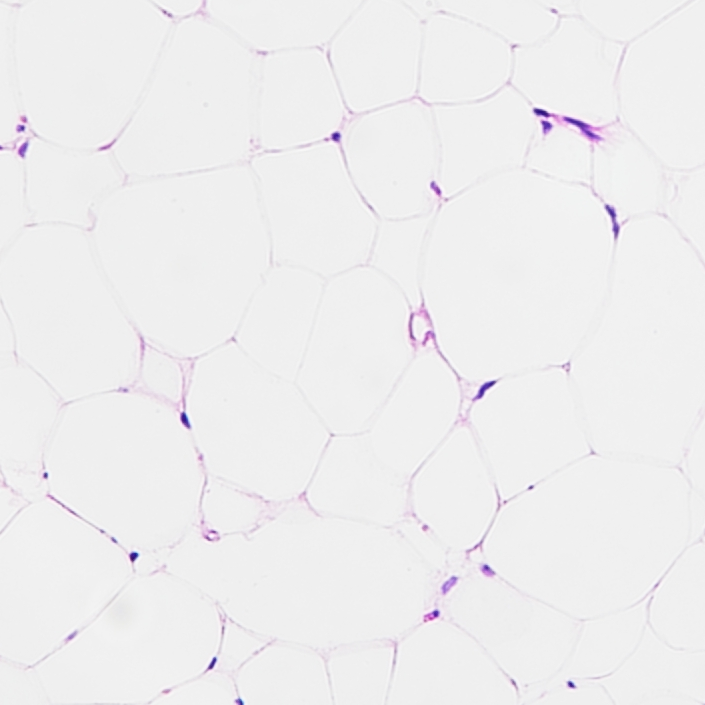

Supplement: Data S1. Raw experimental data generated in this study [file mmc1.zip › All original data/Morphological detection/HE for Figure 2/HFD/HFD-5.tif]

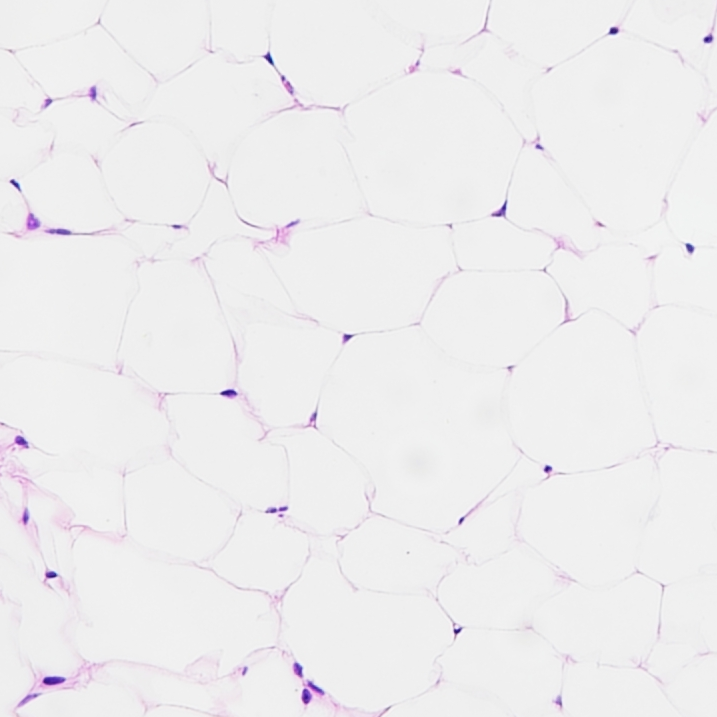

Supplement: Data S1. Raw experimental data generated in this study [file mmc1.zip › All original data/Morphological detection/HE for Figure 2/HFD/HFD-6.tif]

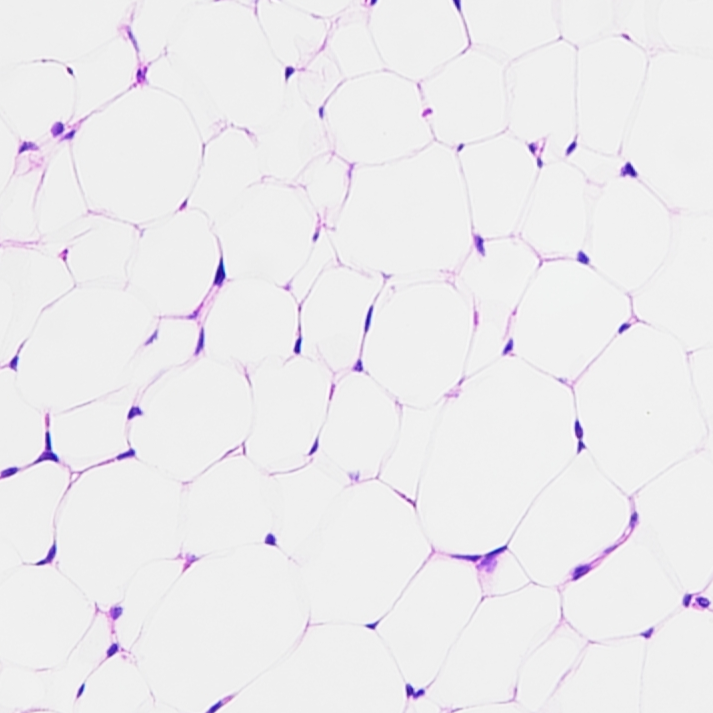

Supplement: Data S1. Raw experimental data generated in this study [file mmc1.zip › All original data/Morphological detection/HE for Figure 2/HFD+ADSC-EXOs/HFD+ADSC-EXOs-1 used for manuscript.tif]

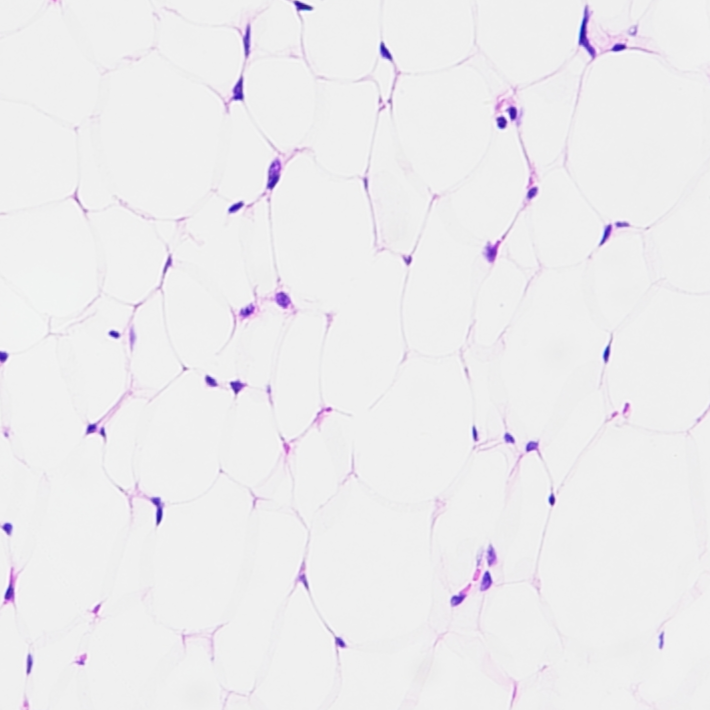

Supplement: Data S1. Raw experimental data generated in this study [file mmc1.zip › All original data/Morphological detection/HE for Figure 2/HFD+ADSC-EXOs/HFD+ADSC-EXOs-2.tif]

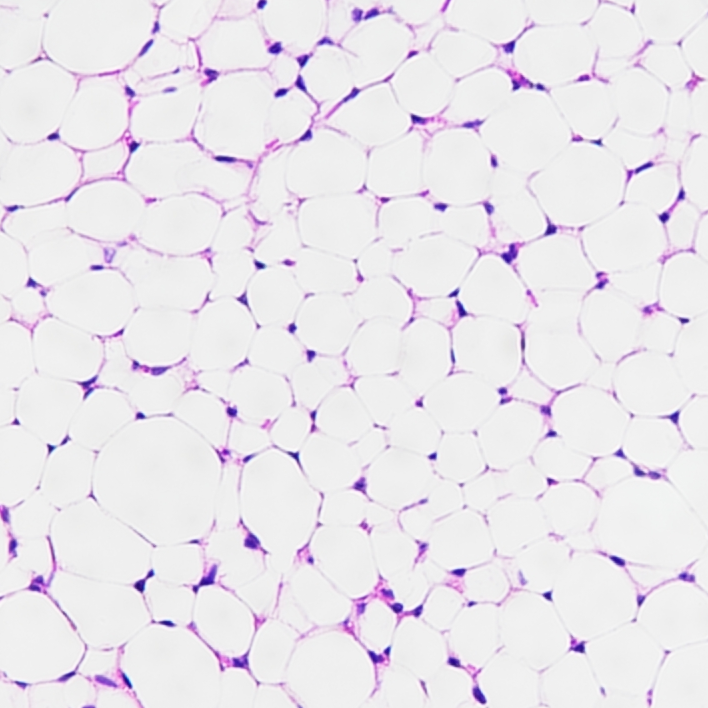

Supplement: Data S1. Raw experimental data generated in this study [file mmc1.zip › All original data/Morphological detection/HE for Figure 2/HFD+ADSC-EXOs/HFD+ADSC-EXOs-3.tif]

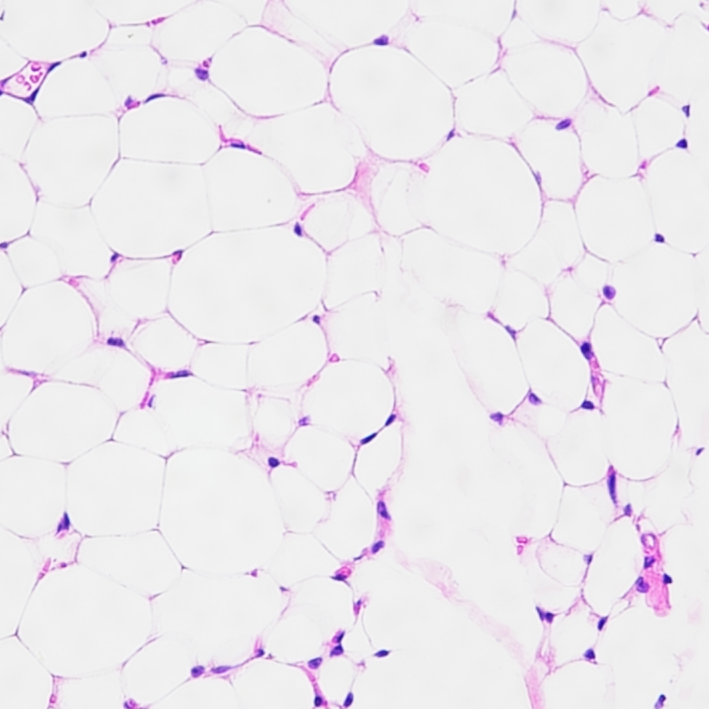

Supplement: Data S1. Raw experimental data generated in this study [file mmc1.zip › All original data/Morphological detection/HE for Figure 2/HFD+ADSC-EXOs/HFD+ADSC-EXOs-4.tif]

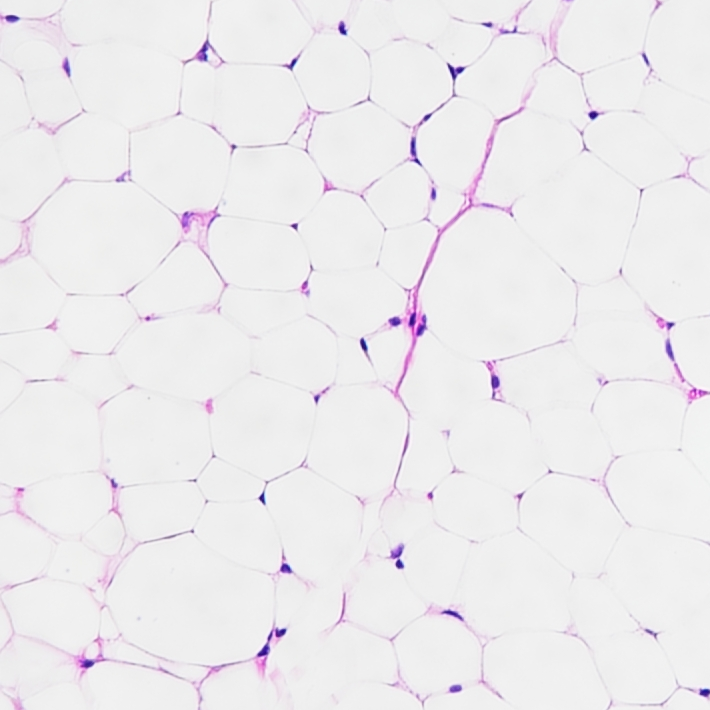

Supplement: Data S1. Raw experimental data generated in this study [file mmc1.zip › All original data/Morphological detection/HE for Figure 2/HFD+ADSC-EXOs/HFD+ADSC-EXOs-5.tif]

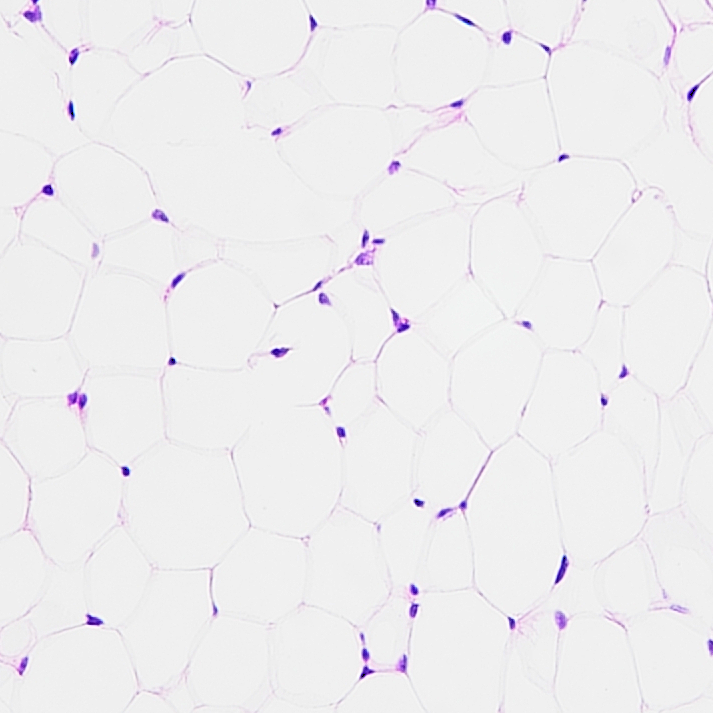

Supplement: Data S1. Raw experimental data generated in this study [file mmc1.zip › All original data/Morphological detection/HE for Figure 2/HFD+ADSC-EXOs/HFD+ADSC-EXOs-6.tif]

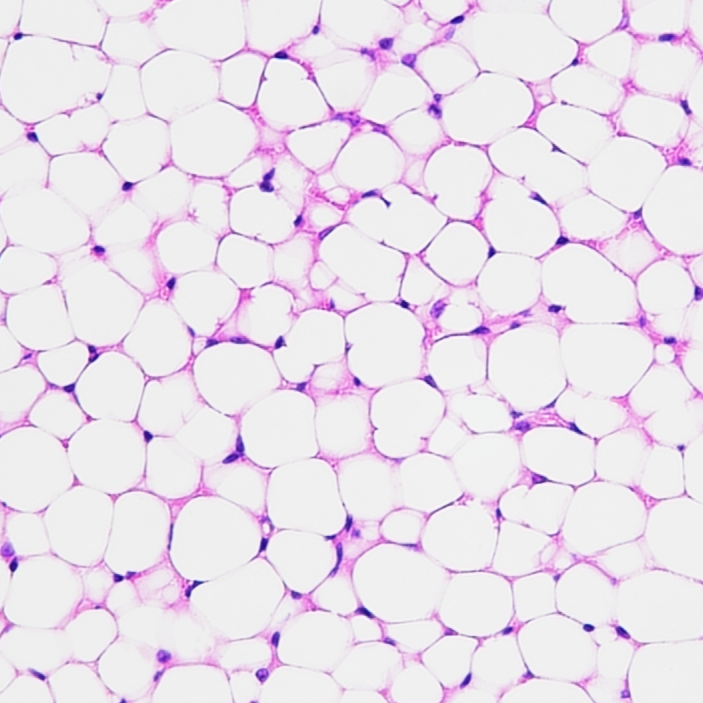

Supplement: Data S1. Raw experimental data generated in this study [file mmc1.zip › All original data/Morphological detection/HE for Figure 2/NFD/NFD-1 used for manuscript.tif]

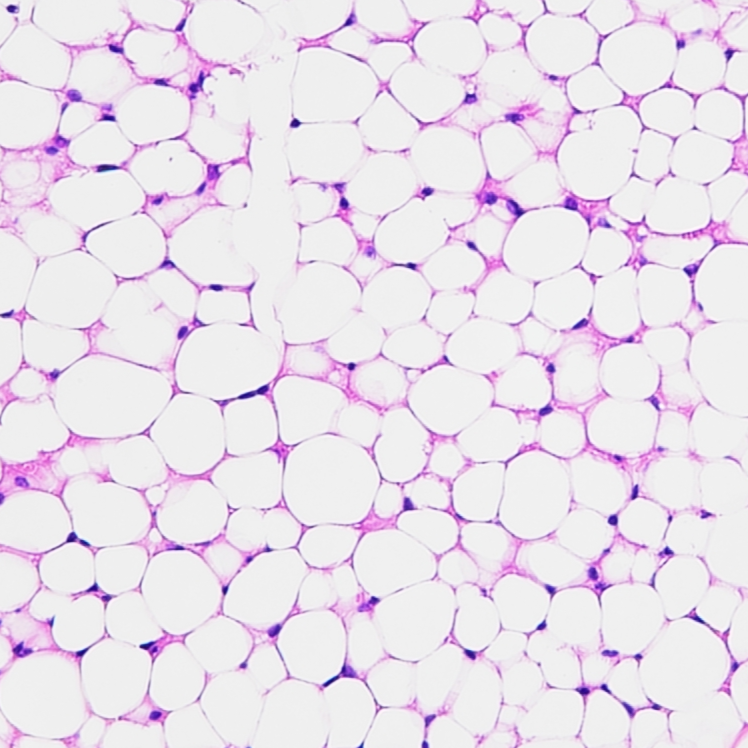

Supplement: Data S1. Raw experimental data generated in this study [file mmc1.zip › All original data/Morphological detection/HE for Figure 2/NFD/NFD-2.tif]

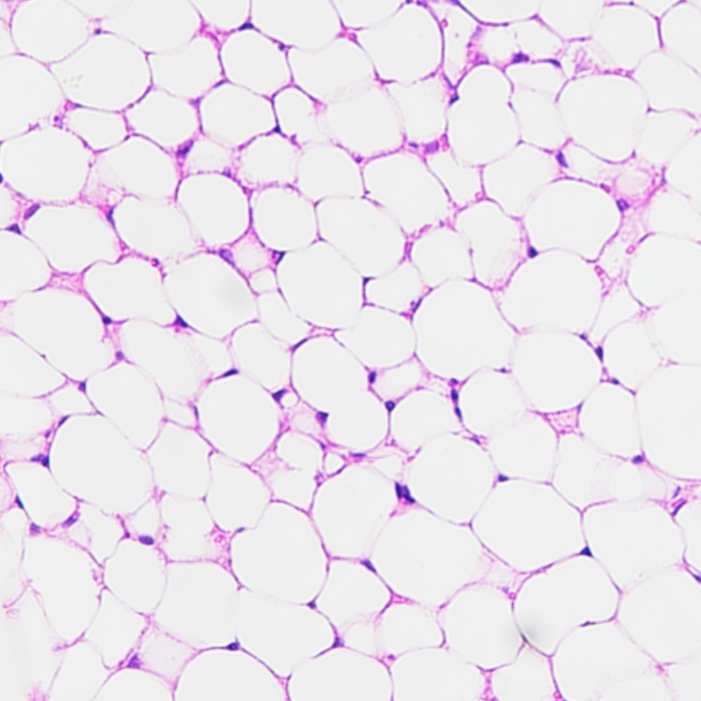

Supplement: Data S1. Raw experimental data generated in this study [file mmc1.zip › All original data/Morphological detection/HE for Figure 2/NFD/NFD-3.tif]

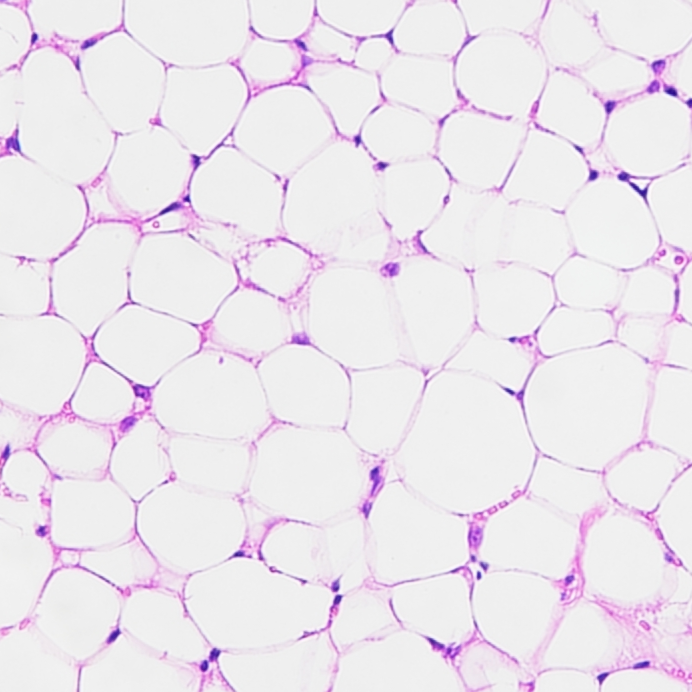

Supplement: Data S1. Raw experimental data generated in this study [file mmc1.zip › All original data/Morphological detection/HE for Figure 2/NFD/NFD-4.tif]

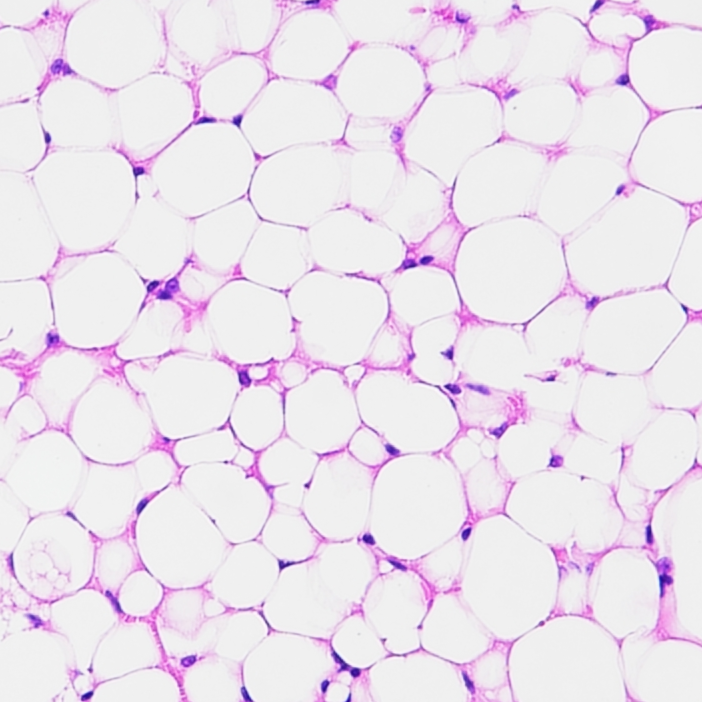

Supplement: Data S1. Raw experimental data generated in this study [file mmc1.zip › All original data/Morphological detection/HE for Figure 2/NFD/NFD-5.tif]

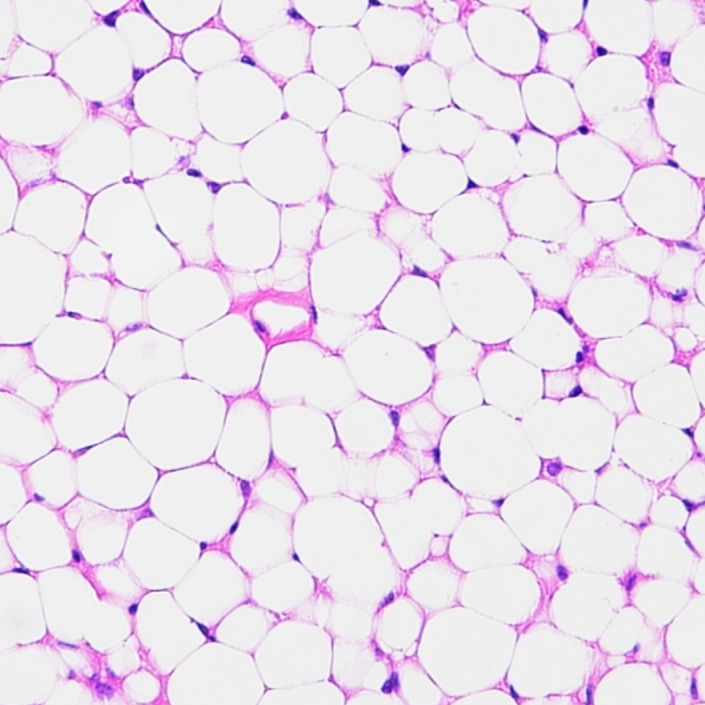

Supplement: Data S1. Raw experimental data generated in this study [file mmc1.zip › All original data/Morphological detection/HE for Figure 2/NFD/NFD-6.tif]

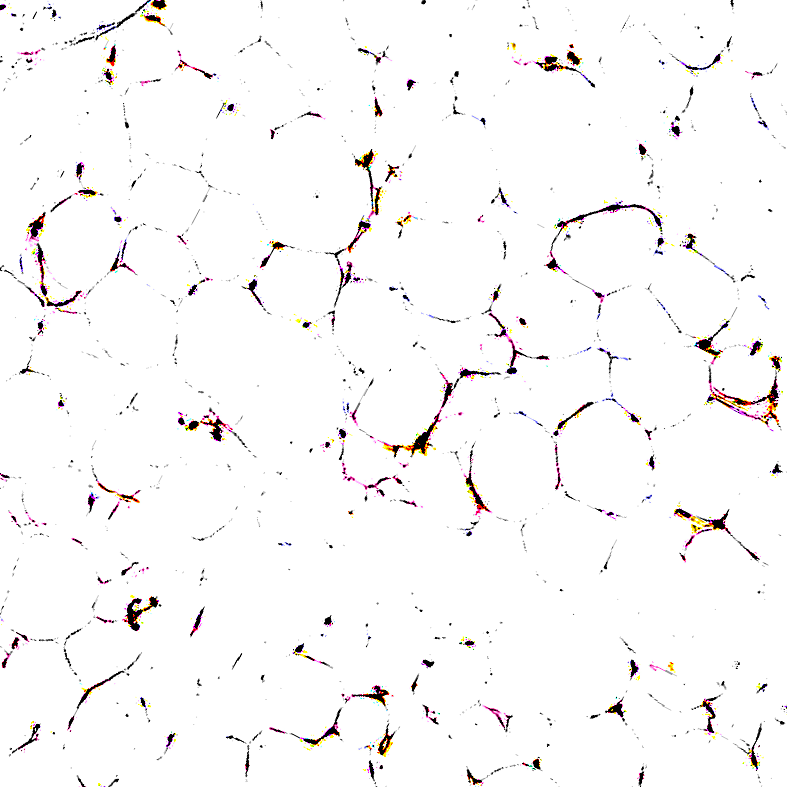

Supplement: Data S1. Raw experimental data generated in this study [file mmc1.zip › All original data/Morphological detection/IHC for Figure 3/IHC for PDCD4/HFD/HFD-1 used for manuscript.tif]

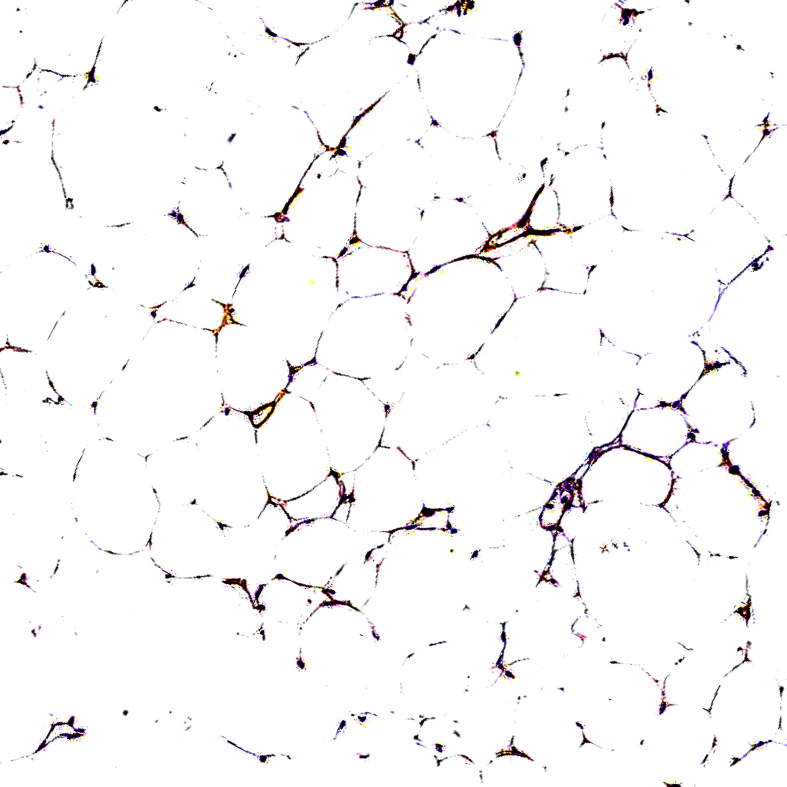

Supplement: Data S1. Raw experimental data generated in this study [file mmc1.zip › All original data/Morphological detection/IHC for Figure 3/IHC for PDCD4/HFD/HFD-2.tif]

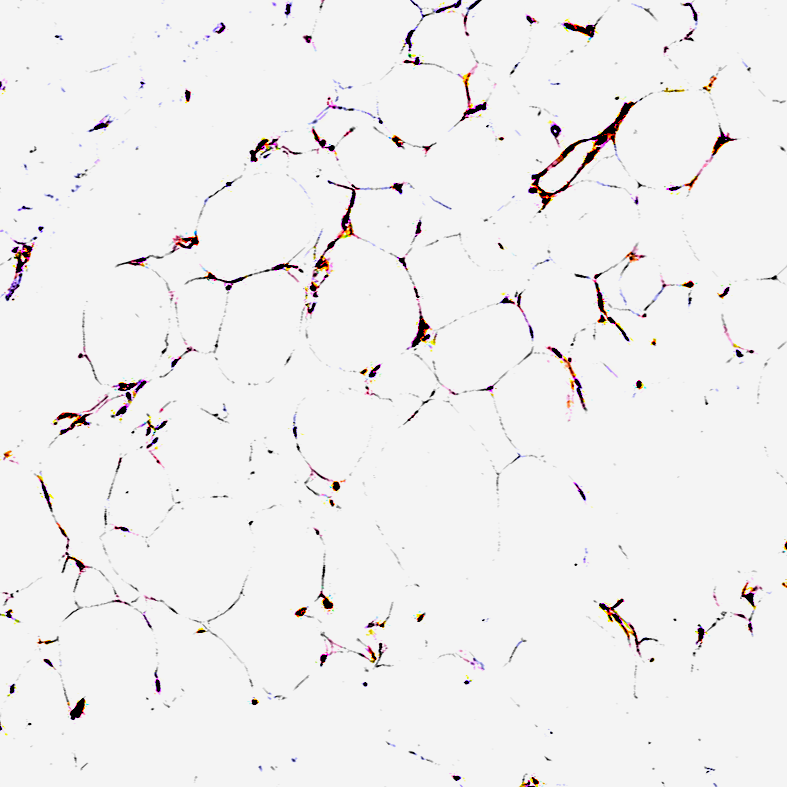

Supplement: Data S1. Raw experimental data generated in this study [file mmc1.zip › All original data/Morphological detection/IHC for Figure 3/IHC for PDCD4/HFD/HFD-3.tif]

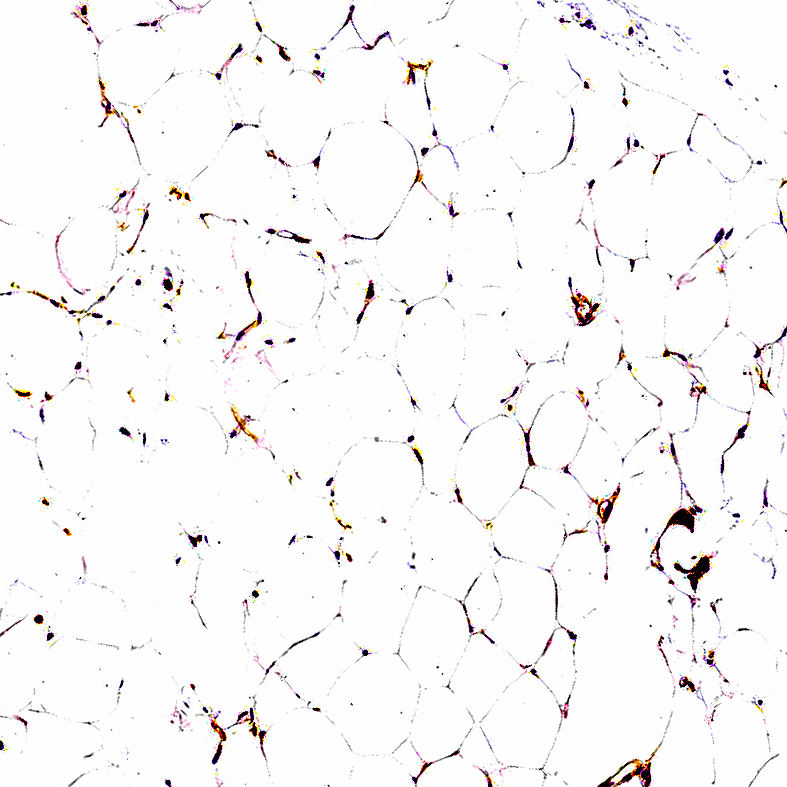

Supplement: Data S1. Raw experimental data generated in this study [file mmc1.zip › All original data/Morphological detection/IHC for Figure 3/IHC for PDCD4/HFD/HFD-4.tif]

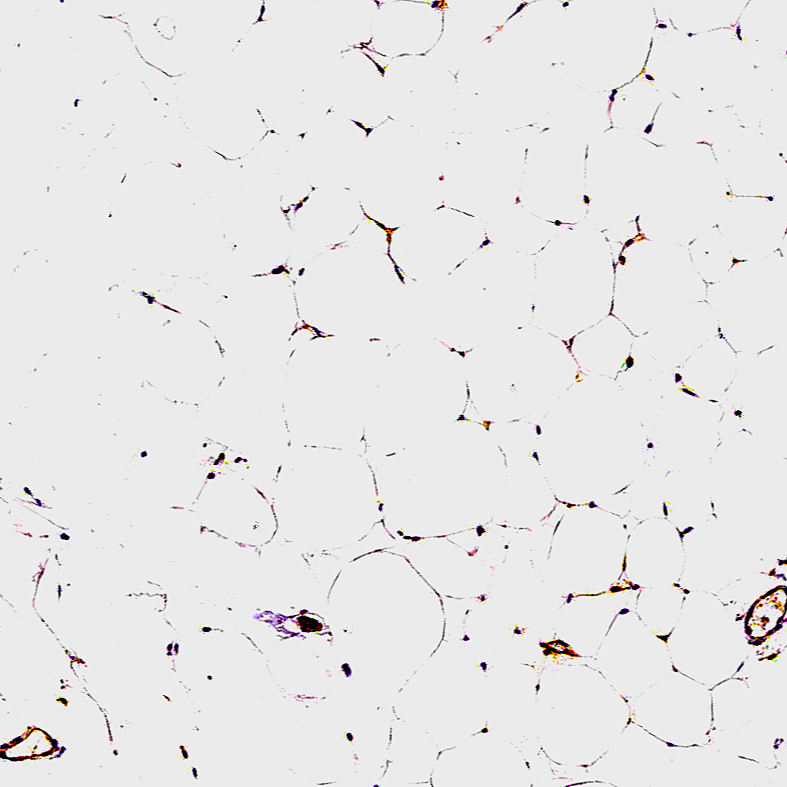

Supplement: Data S1. Raw experimental data generated in this study [file mmc1.zip › All original data/Morphological detection/IHC for Figure 3/IHC for PDCD4/HFD/HFD-5.tif]

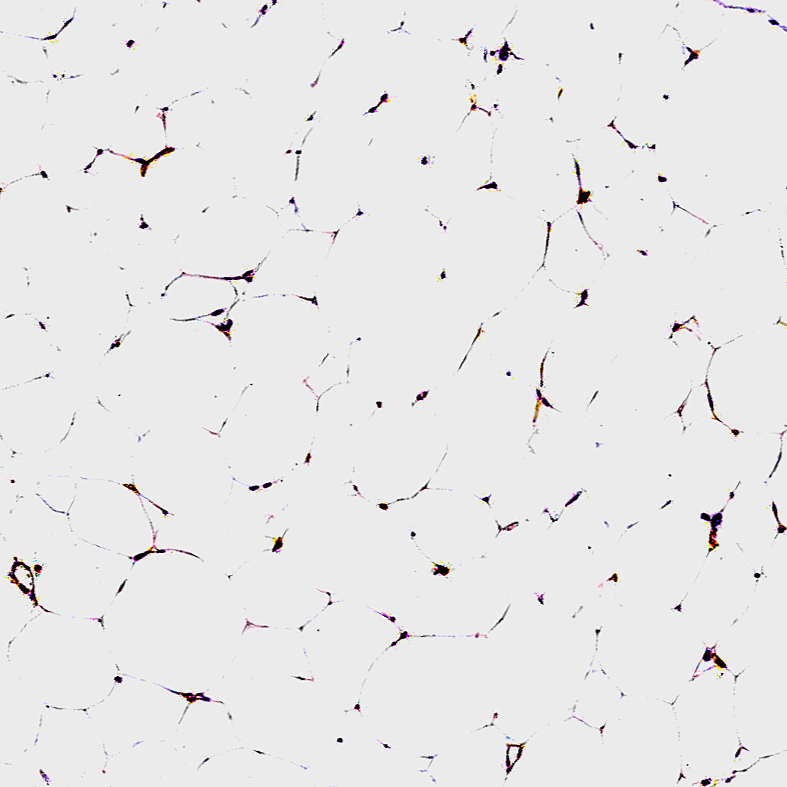

Supplement: Data S1. Raw experimental data generated in this study [file mmc1.zip › All original data/Morphological detection/IHC for Figure 3/IHC for PDCD4/HFD/HFD-6.tif]

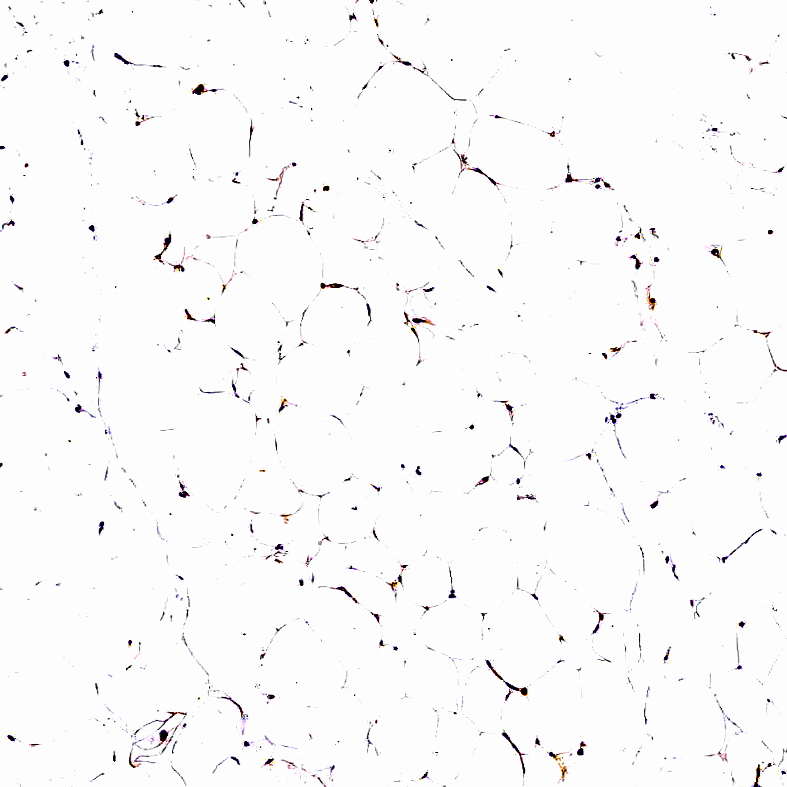

Supplement: Data S1. Raw experimental data generated in this study [file mmc1.zip › All original data/Morphological detection/IHC for Figure 3/IHC for PDCD4/HFD+ADSC-EXOs/HFD+ADSC-EX0s-1 used for manuscript.tif]

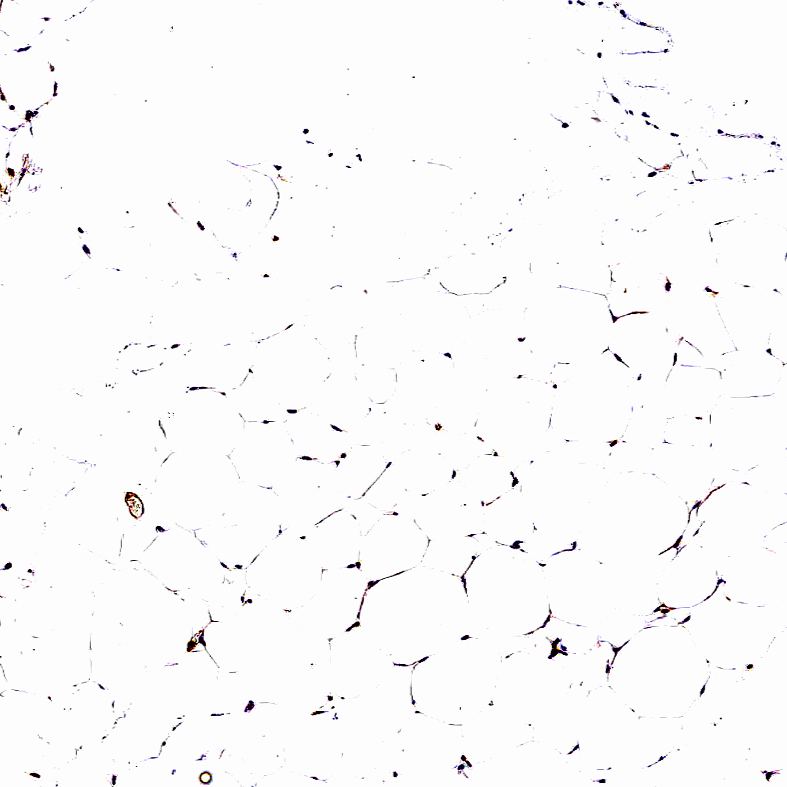

Supplement: Data S1. Raw experimental data generated in this study [file mmc1.zip › All original data/Morphological detection/IHC for Figure 3/IHC for PDCD4/HFD+ADSC-EXOs/HFD+ADSC-EX0s-2.tif]

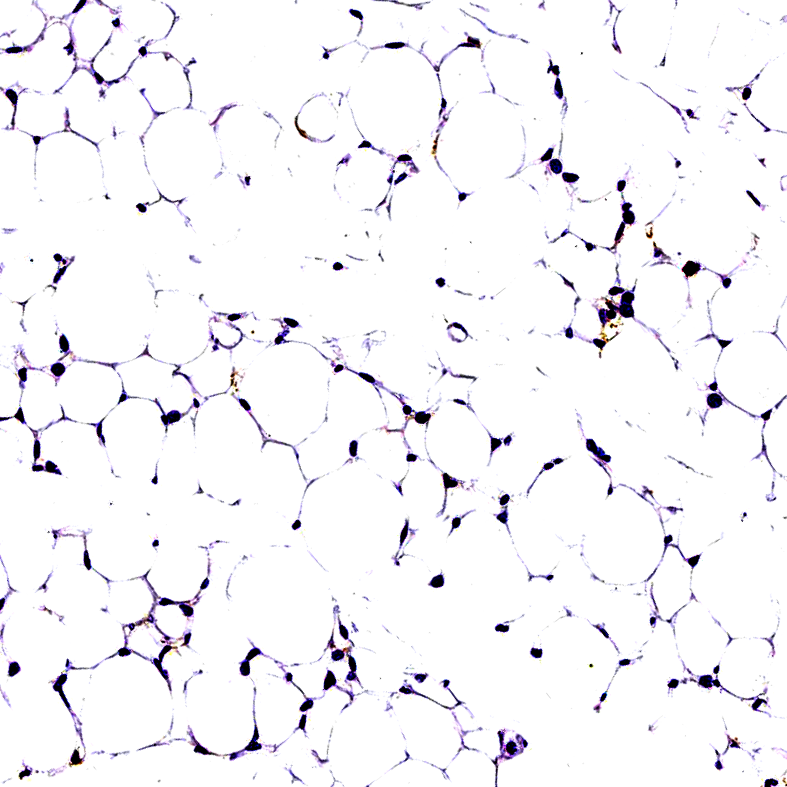

Supplement: Data S1. Raw experimental data generated in this study [file mmc1.zip › All original data/Morphological detection/IHC for Figure 3/IHC for PDCD4/HFD+ADSC-EXOs/HFD+ADSC-EX0s-3.tif]

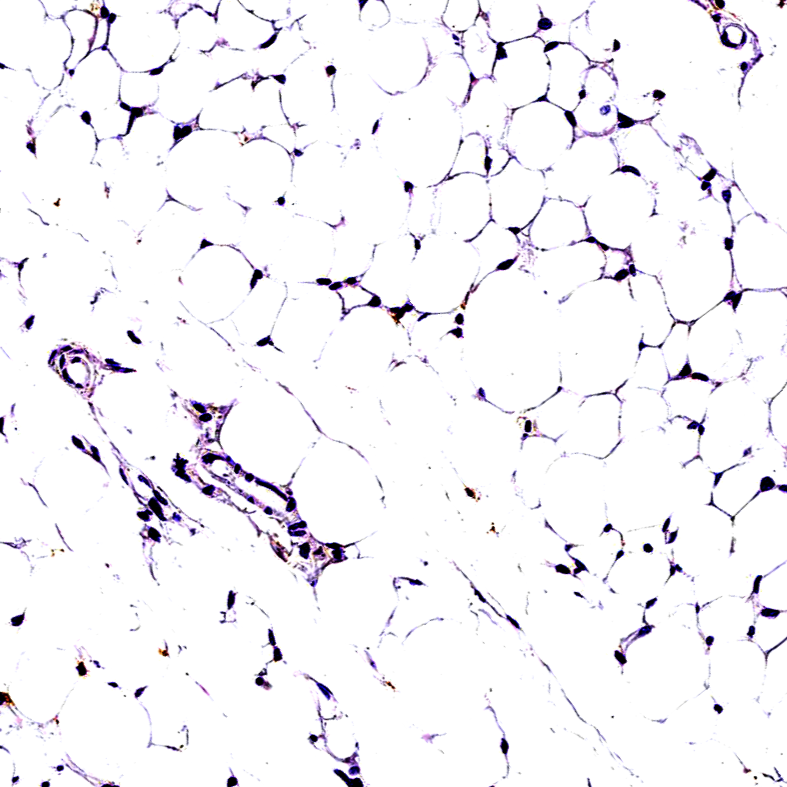

Supplement: Data S1. Raw experimental data generated in this study [file mmc1.zip › All original data/Morphological detection/IHC for Figure 3/IHC for PDCD4/HFD+ADSC-EXOs/HFD+ADSC-EX0s-4.tif]

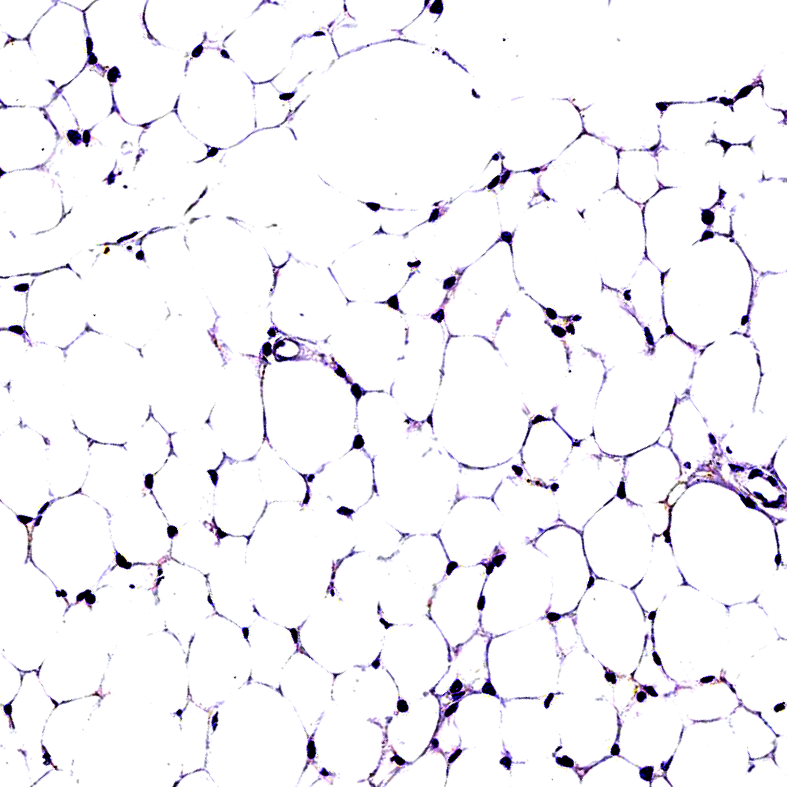

Supplement: Data S1. Raw experimental data generated in this study [file mmc1.zip › All original data/Morphological detection/IHC for Figure 3/IHC for PDCD4/HFD+ADSC-EXOs/HFD+ADSC-EX0s-5.tif]

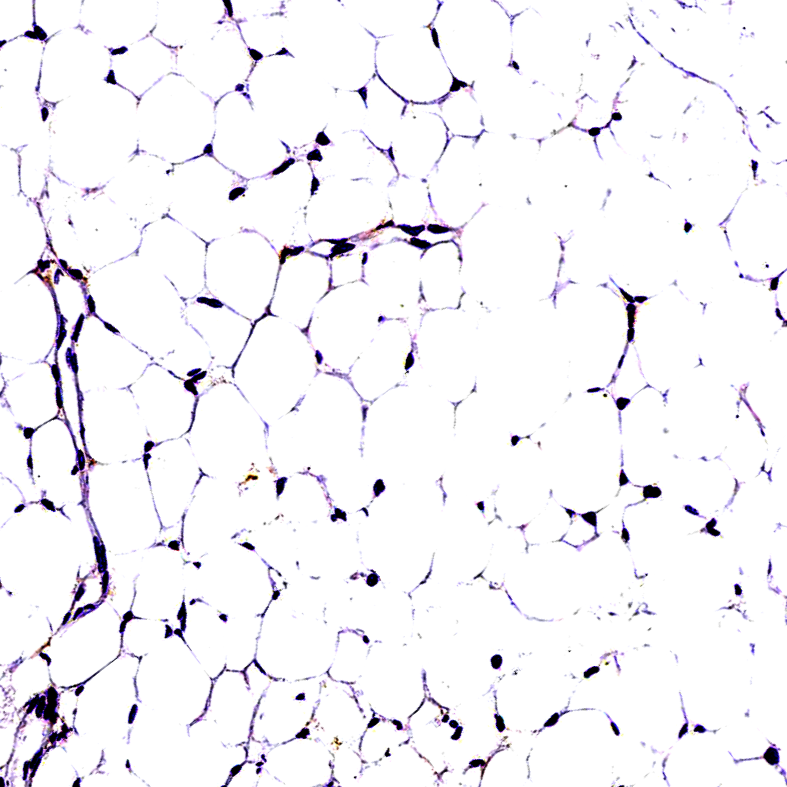

Supplement: Data S1. Raw experimental data generated in this study [file mmc1.zip › All original data/Morphological detection/IHC for Figure 3/IHC for PDCD4/HFD+ADSC-EXOs/HFD+ADSC-EX0s-6.tif]

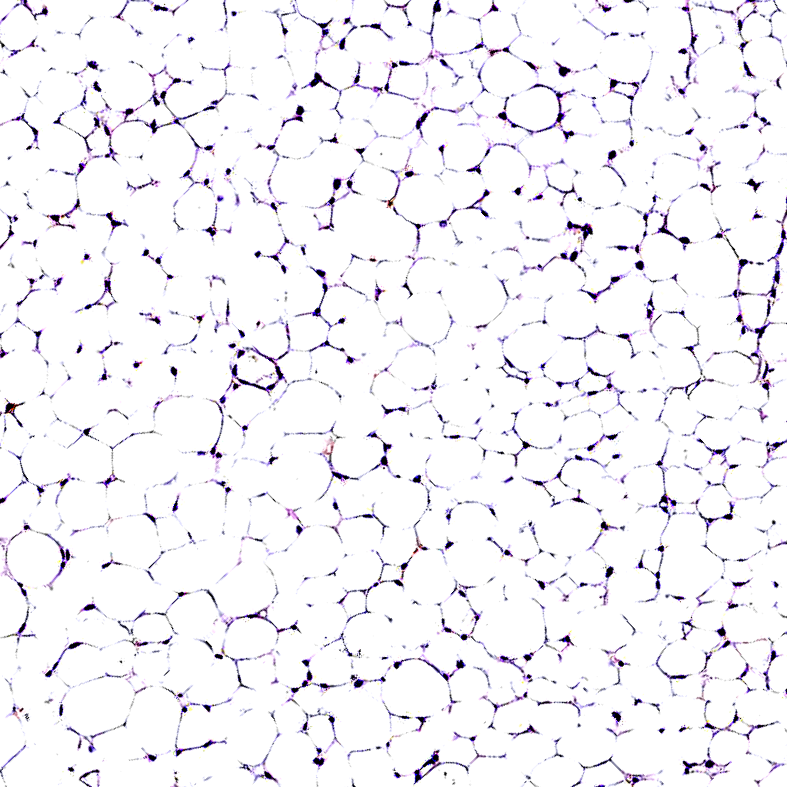

Supplement: Data S1. Raw experimental data generated in this study [file mmc1.zip › All original data/Morphological detection/IHC for Figure 3/IHC for PDCD4/NFD/NFD-1 used for manuscript.tif]

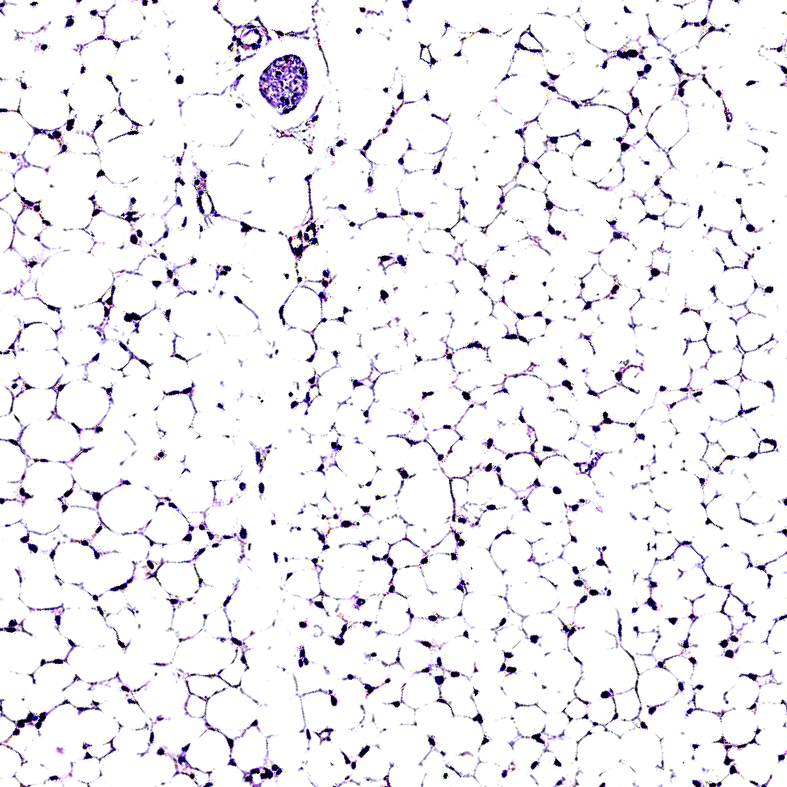

Supplement: Data S1. Raw experimental data generated in this study [file mmc1.zip › All original data/Morphological detection/IHC for Figure 3/IHC for PDCD4/NFD/NFD-2.tif]

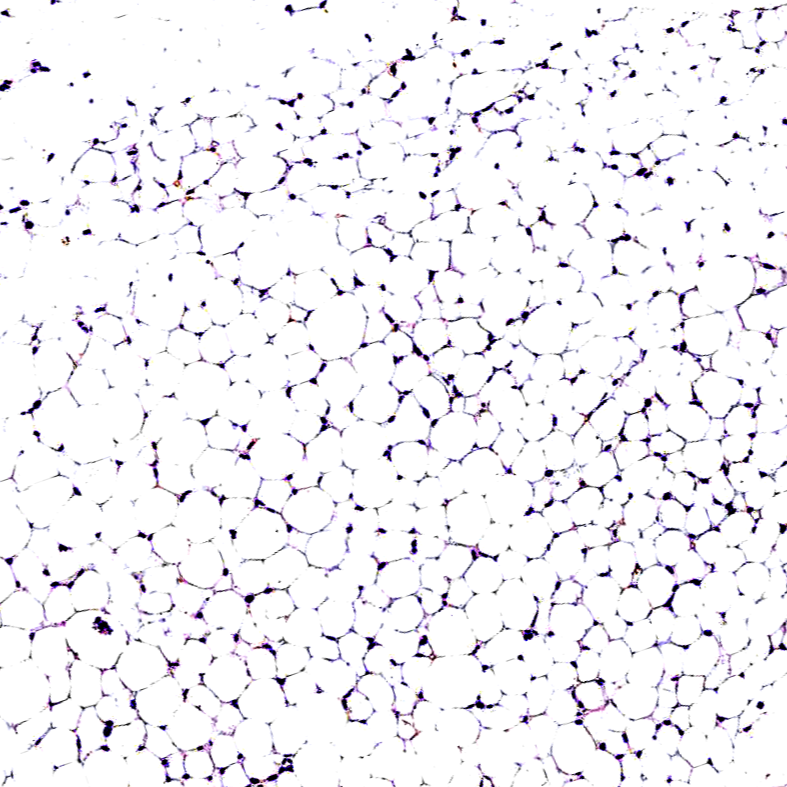

Supplement: Data S1. Raw experimental data generated in this study [file mmc1.zip › All original data/Morphological detection/IHC for Figure 3/IHC for PDCD4/NFD/NFD-3.tif]

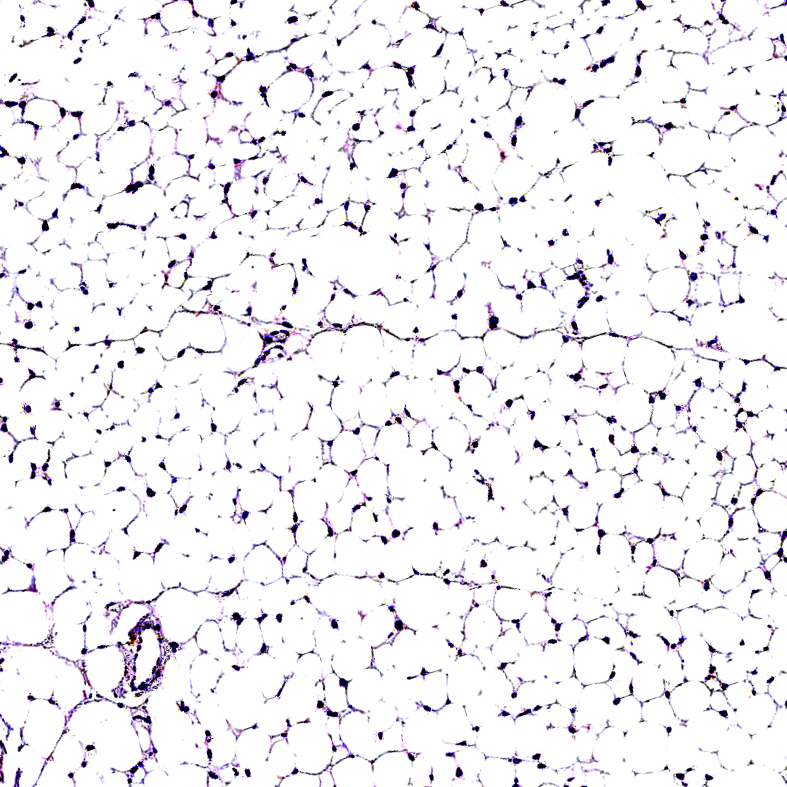

Supplement: Data S1. Raw experimental data generated in this study [file mmc1.zip › All original data/Morphological detection/IHC for Figure 3/IHC for PDCD4/NFD/NFD-4.tif]

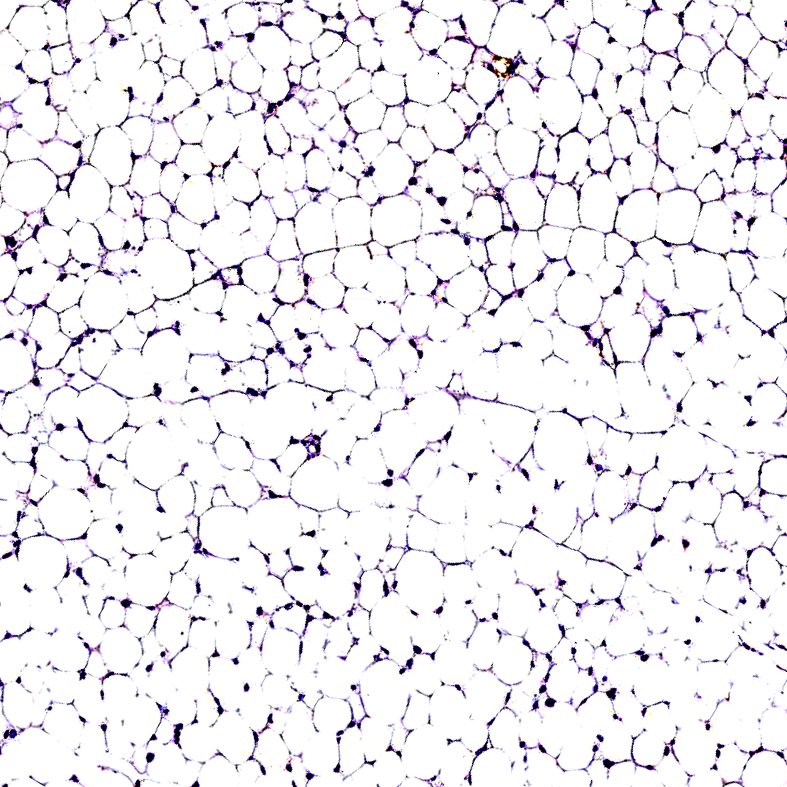

Supplement: Data S1. Raw experimental data generated in this study [file mmc1.zip › All original data/Morphological detection/IHC for Figure 3/IHC for PDCD4/NFD/NFD-5.tif]

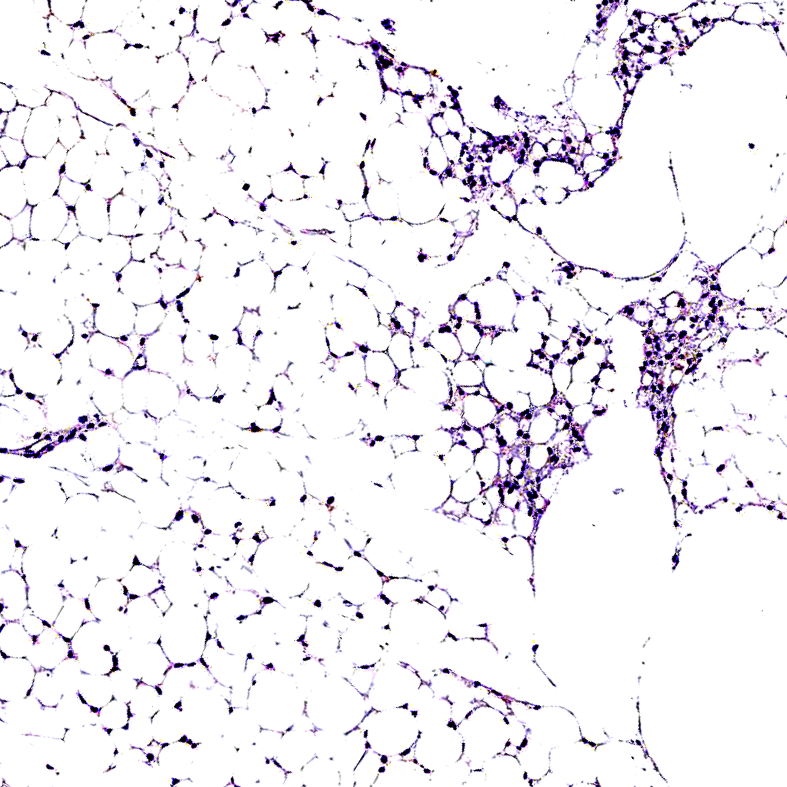

Supplement: Data S1. Raw experimental data generated in this study [file mmc1.zip › All original data/Morphological detection/IHC for Figure 3/IHC for PDCD4/NFD/NFD-6.tif]

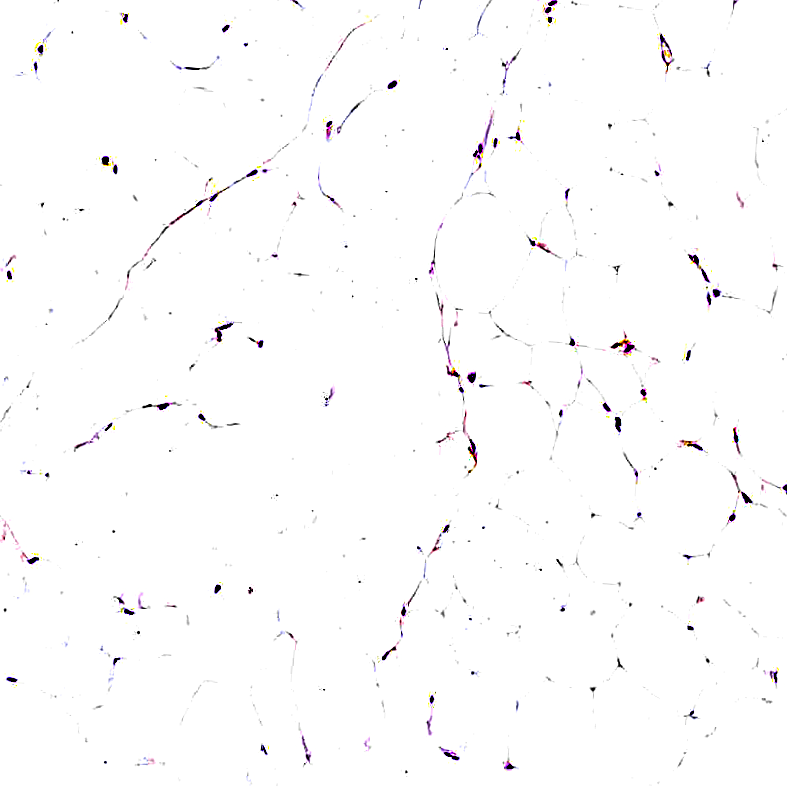

Supplement: Data S1. Raw experimental data generated in this study [file mmc1.zip › All original data/Morphological detection/IHC for Figure 3/IHC for UCP-1/HFD/HFD-1 used for manuscript.tif]

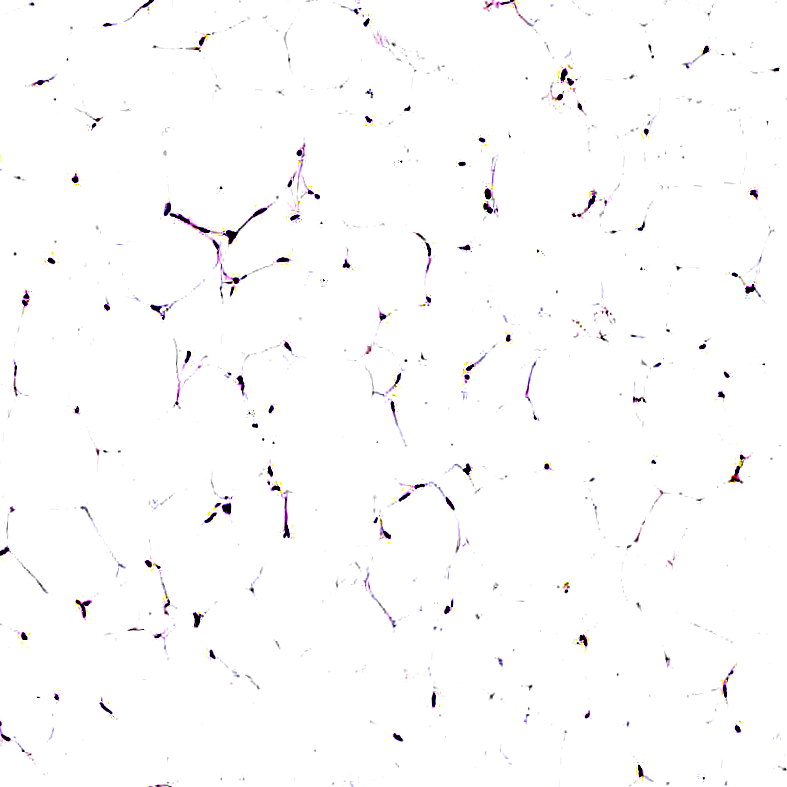

Supplement: Data S1. Raw experimental data generated in this study [file mmc1.zip › All original data/Morphological detection/IHC for Figure 3/IHC for UCP-1/HFD/HFD-2.tif]

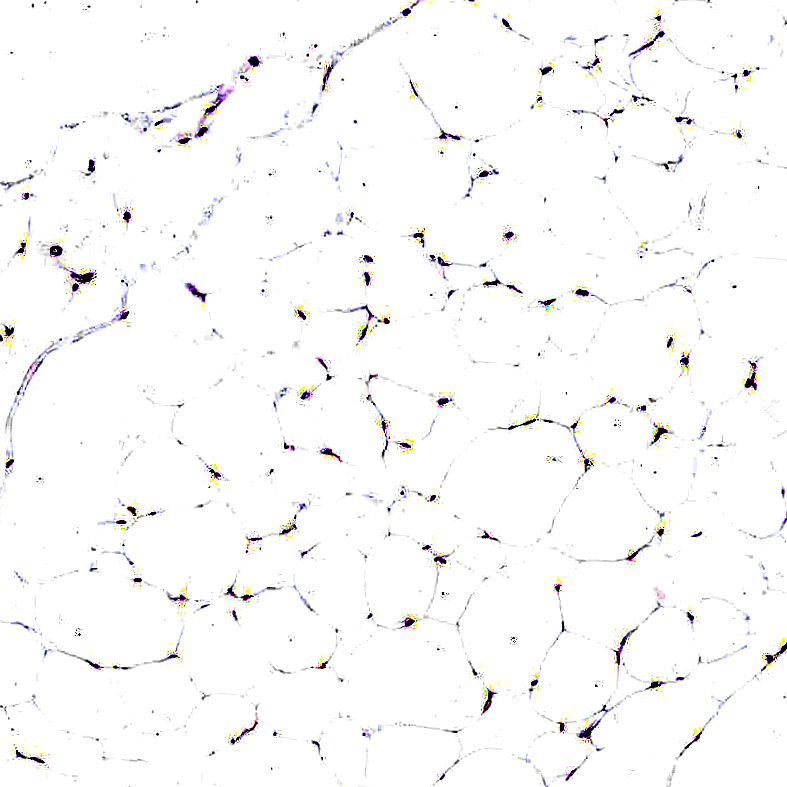

Supplement: Data S1. Raw experimental data generated in this study [file mmc1.zip › All original data/Morphological detection/IHC for Figure 3/IHC for UCP-1/HFD/HFD-3.tif]

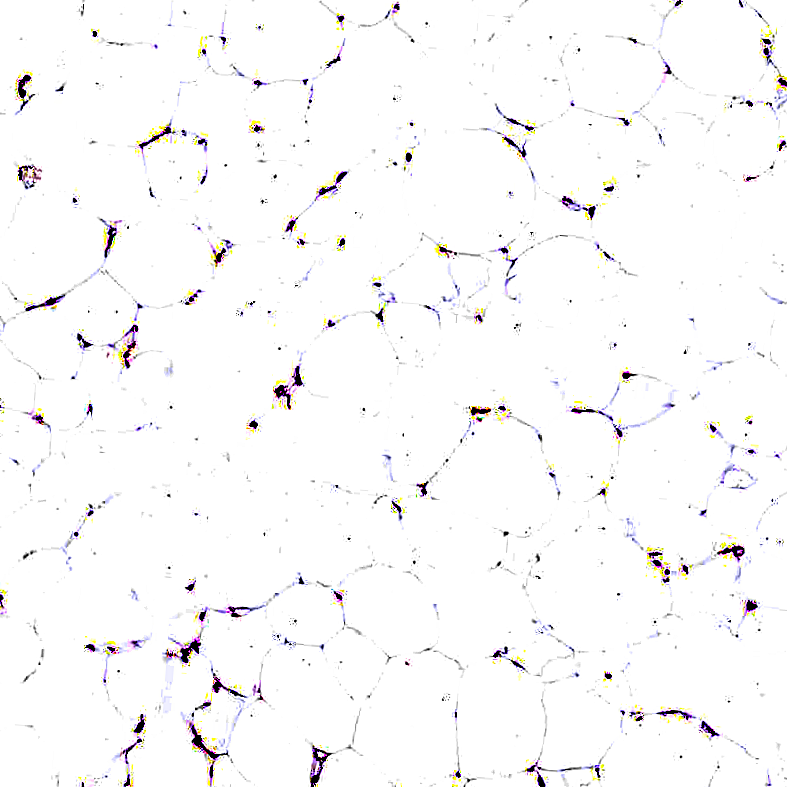

Supplement: Data S1. Raw experimental data generated in this study [file mmc1.zip › All original data/Morphological detection/IHC for Figure 3/IHC for UCP-1/HFD/HFD-4.tif]

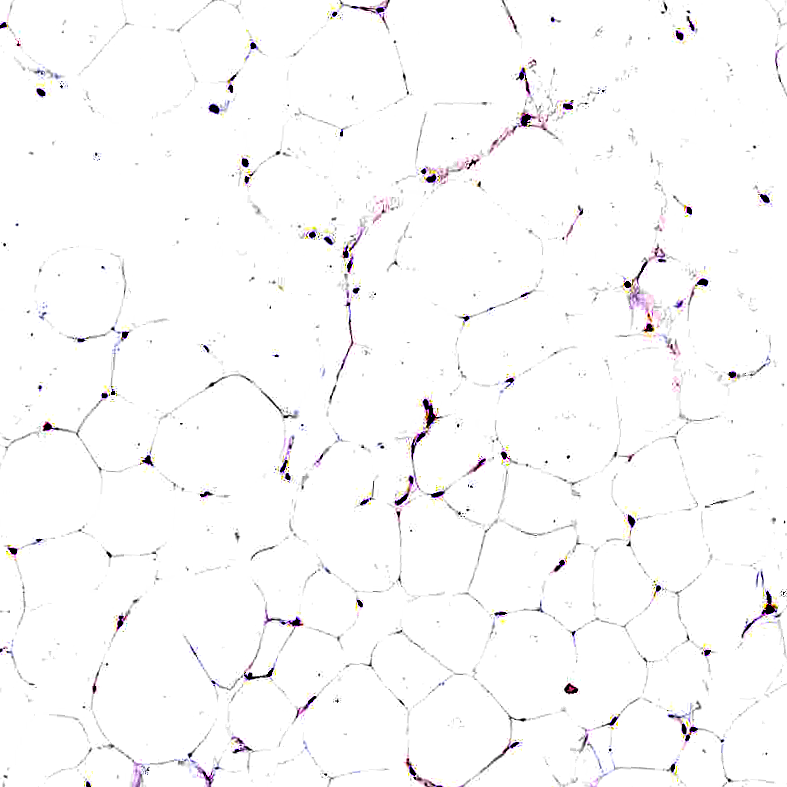

Supplement: Data S1. Raw experimental data generated in this study [file mmc1.zip › All original data/Morphological detection/IHC for Figure 3/IHC for UCP-1/HFD/HFD-5.tif]

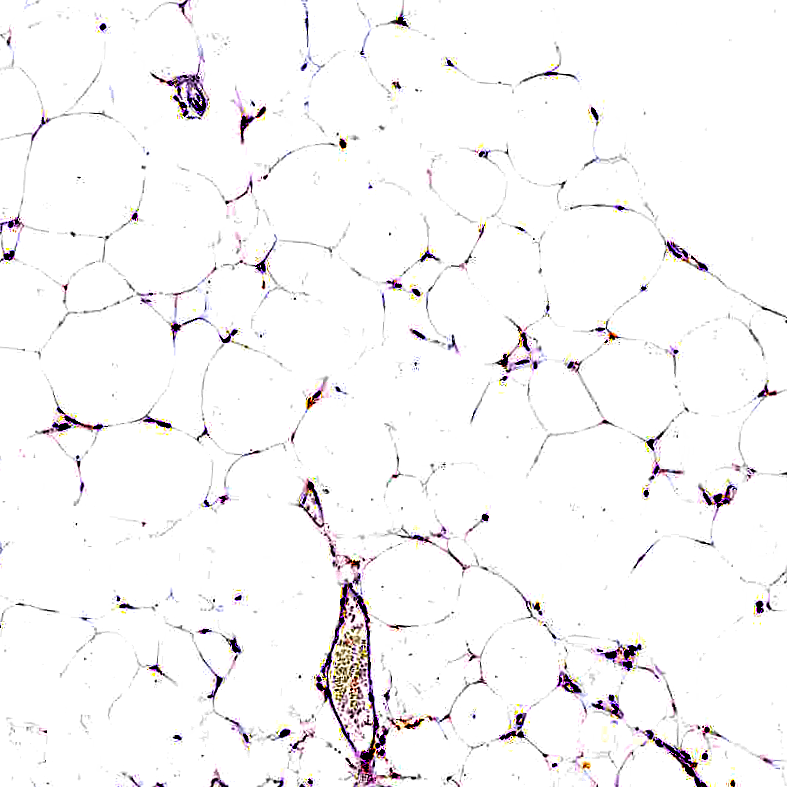

Supplement: Data S1. Raw experimental data generated in this study [file mmc1.zip › All original data/Morphological detection/IHC for Figure 3/IHC for UCP-1/HFD/HFD-6.tif]

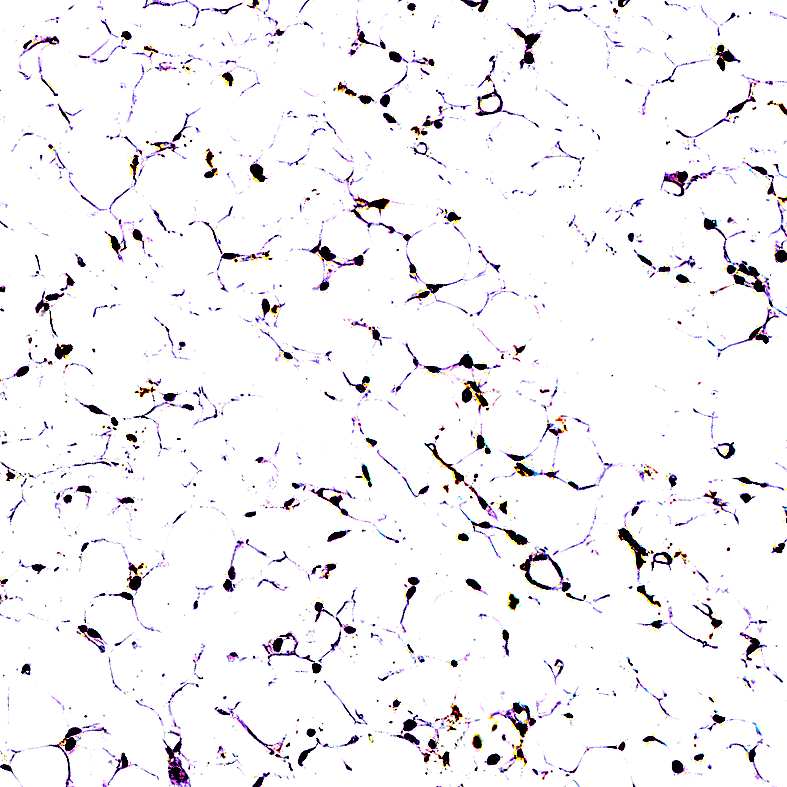

Supplement: Data S1. Raw experimental data generated in this study [file mmc1.zip › All original data/Morphological detection/IHC for Figure 3/IHC for UCP-1/HFD+ADSC-EXOs/HFD+ADSC-EXO-1 used for manuscript.tif]

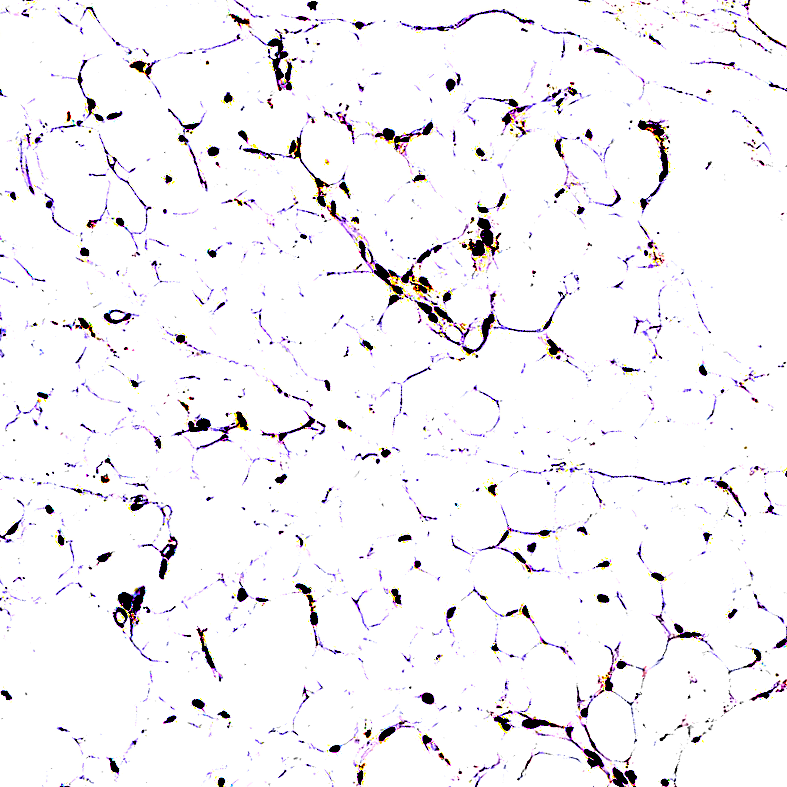

Supplement: Data S1. Raw experimental data generated in this study [file mmc1.zip › All original data/Morphological detection/IHC for Figure 3/IHC for UCP-1/HFD+ADSC-EXOs/HFD+ADSC-EXO-2.tif]

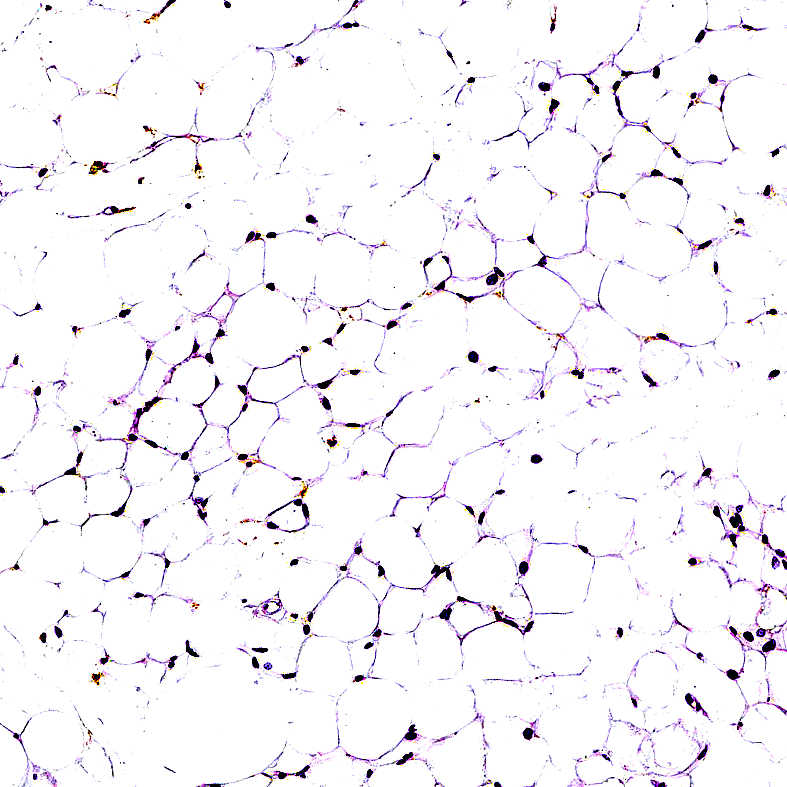

Supplement: Data S1. Raw experimental data generated in this study [file mmc1.zip › All original data/Morphological detection/IHC for Figure 3/IHC for UCP-1/HFD+ADSC-EXOs/HFD+ADSC-EXO-3.tif]

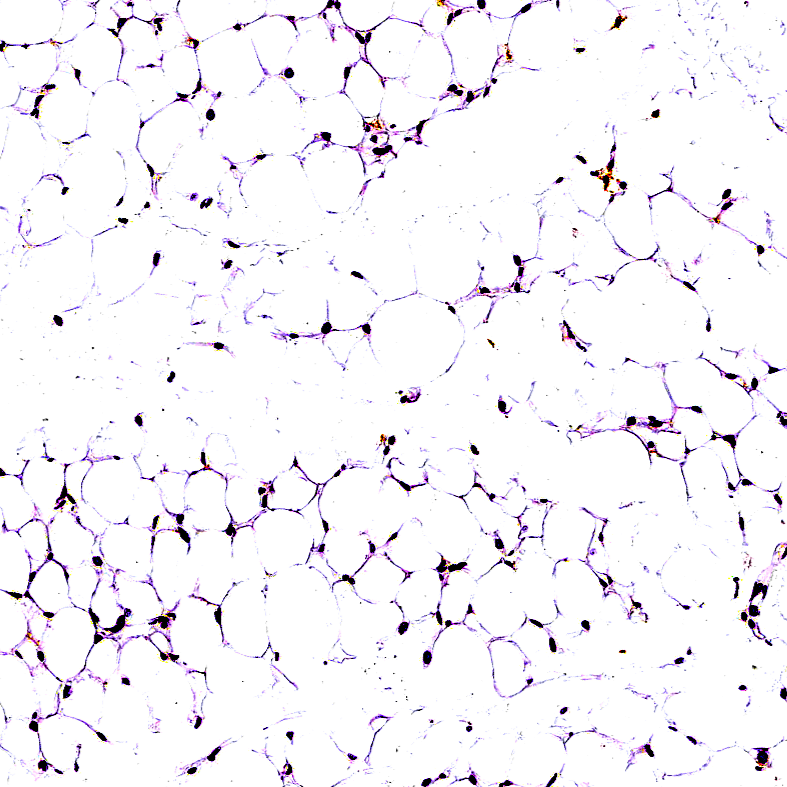

Supplement: Data S1. Raw experimental data generated in this study [file mmc1.zip › All original data/Morphological detection/IHC for Figure 3/IHC for UCP-1/HFD+ADSC-EXOs/HFD+ADSC-EXO-4.tif]

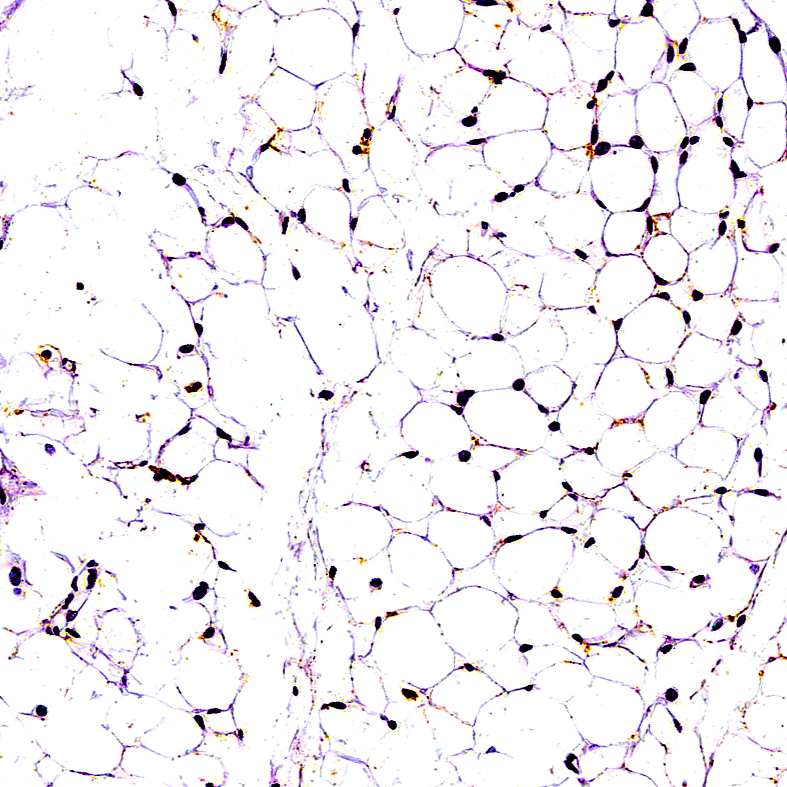

Supplement: Data S1. Raw experimental data generated in this study [file mmc1.zip › All original data/Morphological detection/IHC for Figure 3/IHC for UCP-1/HFD+ADSC-EXOs/HFD+ADSC-EXO-5.tif]

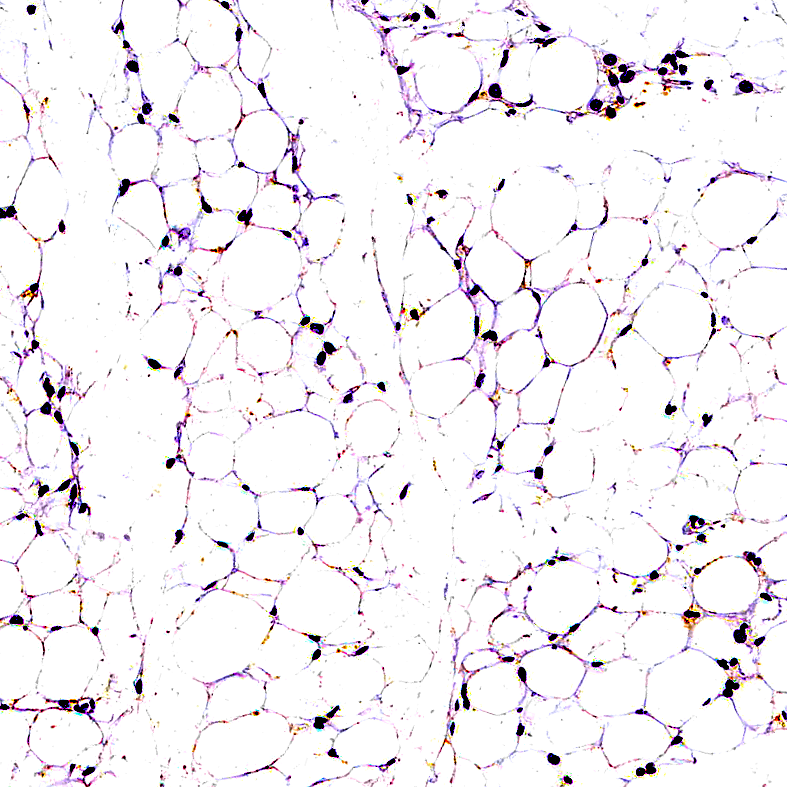

Supplement: Data S1. Raw experimental data generated in this study [file mmc1.zip › All original data/Morphological detection/IHC for Figure 3/IHC for UCP-1/HFD+ADSC-EXOs/HFD+ADSC-EXO-6.tif]

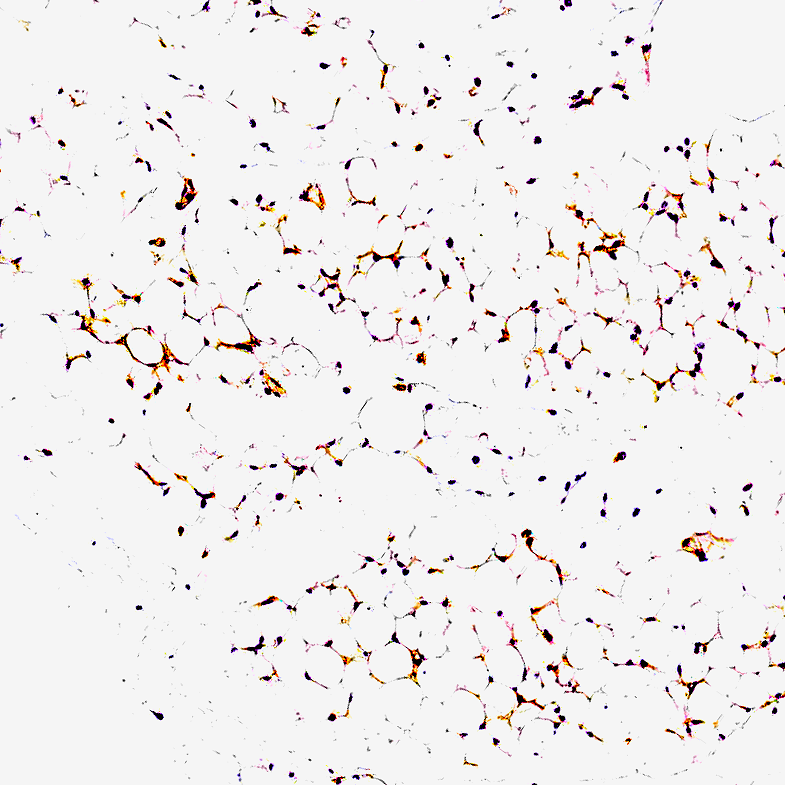

Supplement: Data S1. Raw experimental data generated in this study [file mmc1.zip › All original data/Morphological detection/IHC for Figure 3/IHC for UCP-1/NFD/NFD-1.tif]

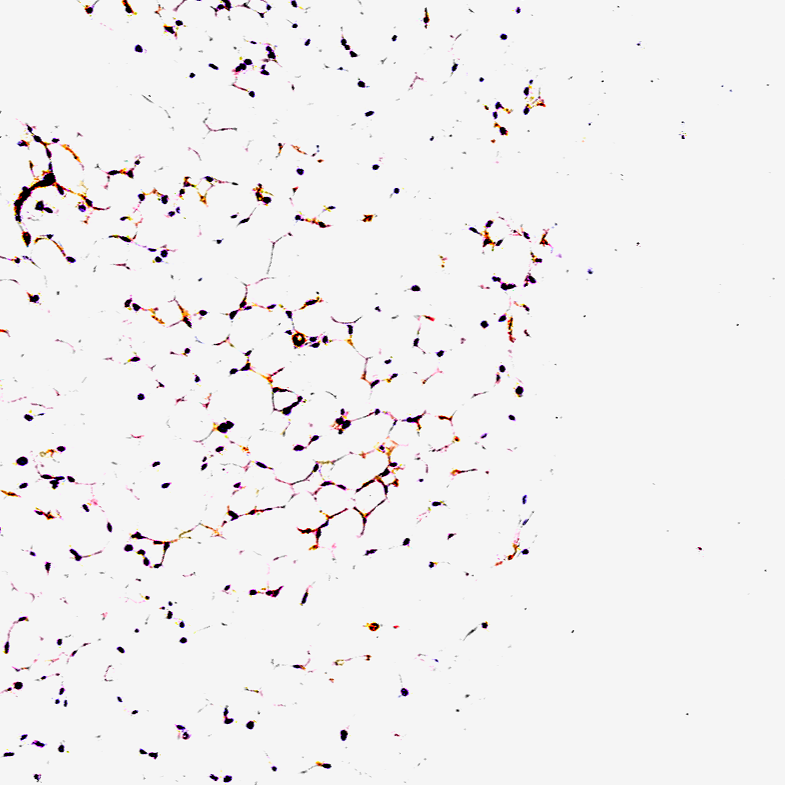

Supplement: Data S1. Raw experimental data generated in this study [file mmc1.zip › All original data/Morphological detection/IHC for Figure 3/IHC for UCP-1/NFD/NFD-2.tif]

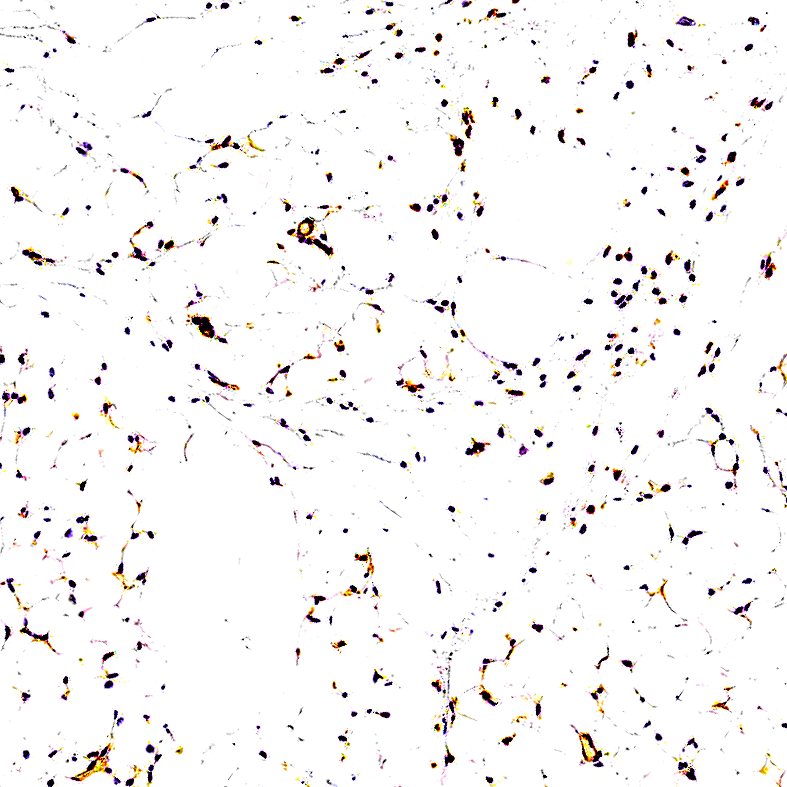

Supplement: Data S1. Raw experimental data generated in this study [file mmc1.zip › All original data/Morphological detection/IHC for Figure 3/IHC for UCP-1/NFD/NFD-3.tif]

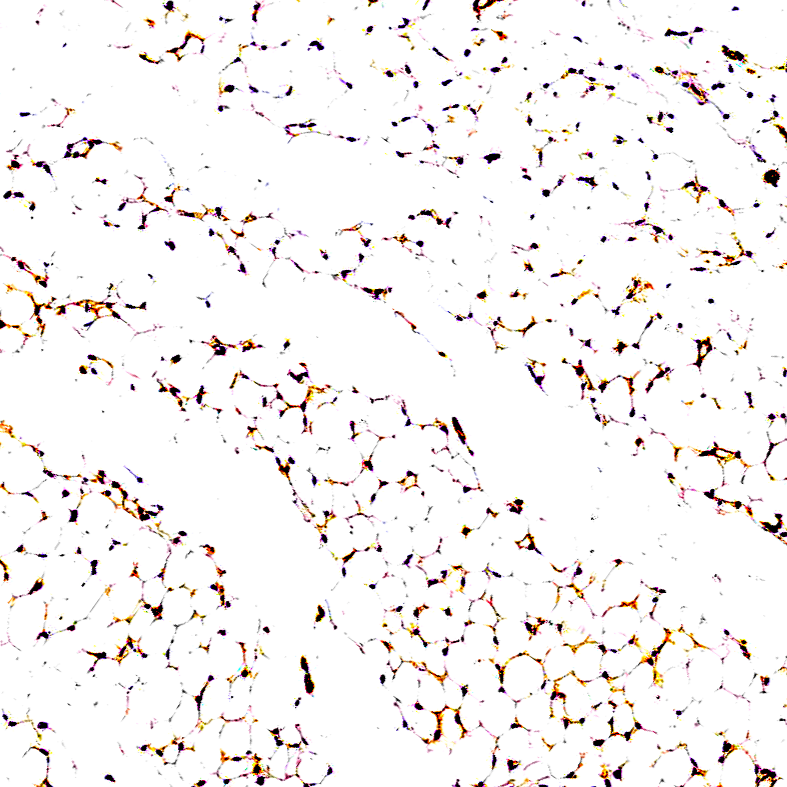

Supplement: Data S1. Raw experimental data generated in this study [file mmc1.zip › All original data/Morphological detection/IHC for Figure 3/IHC for UCP-1/NFD/NFD-4.tif]

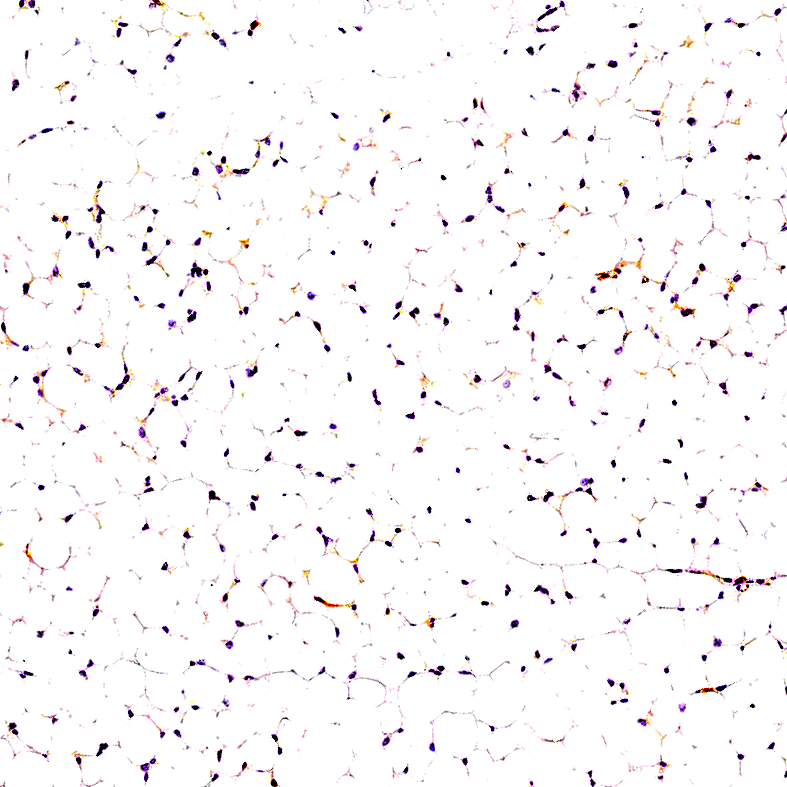

Supplement: Data S1. Raw experimental data generated in this study [file mmc1.zip › All original data/Morphological detection/IHC for Figure 3/IHC for UCP-1/NFD/NFD-5.tif]

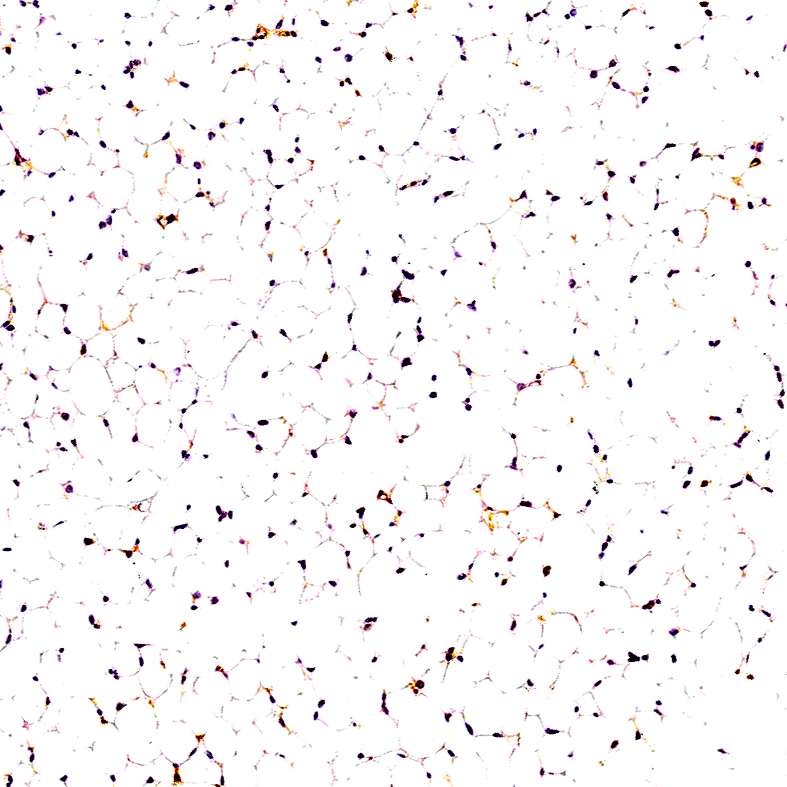

Supplement: Data S1. Raw experimental data generated in this study [file mmc1.zip › All original data/Morphological detection/IHC for Figure 3/IHC for UCP-1/NFD/NFD-6.tif]

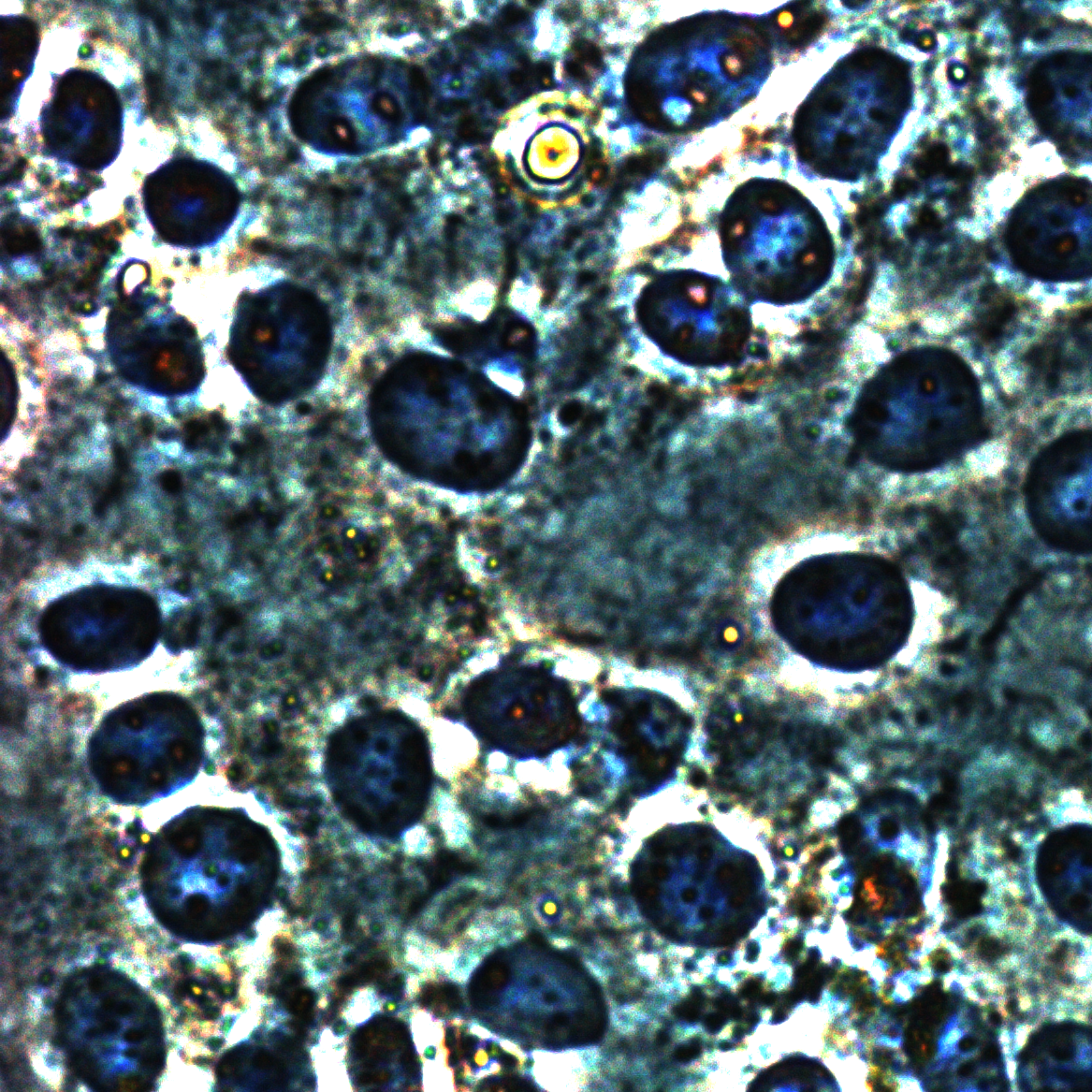

Supplement: Data S1. Raw experimental data generated in this study [file mmc1.zip › All original data/Morphological detection/Oil-red staining/Oil-red staining for Figure 4/Day0/Day0-1 used for manuscript.tif]

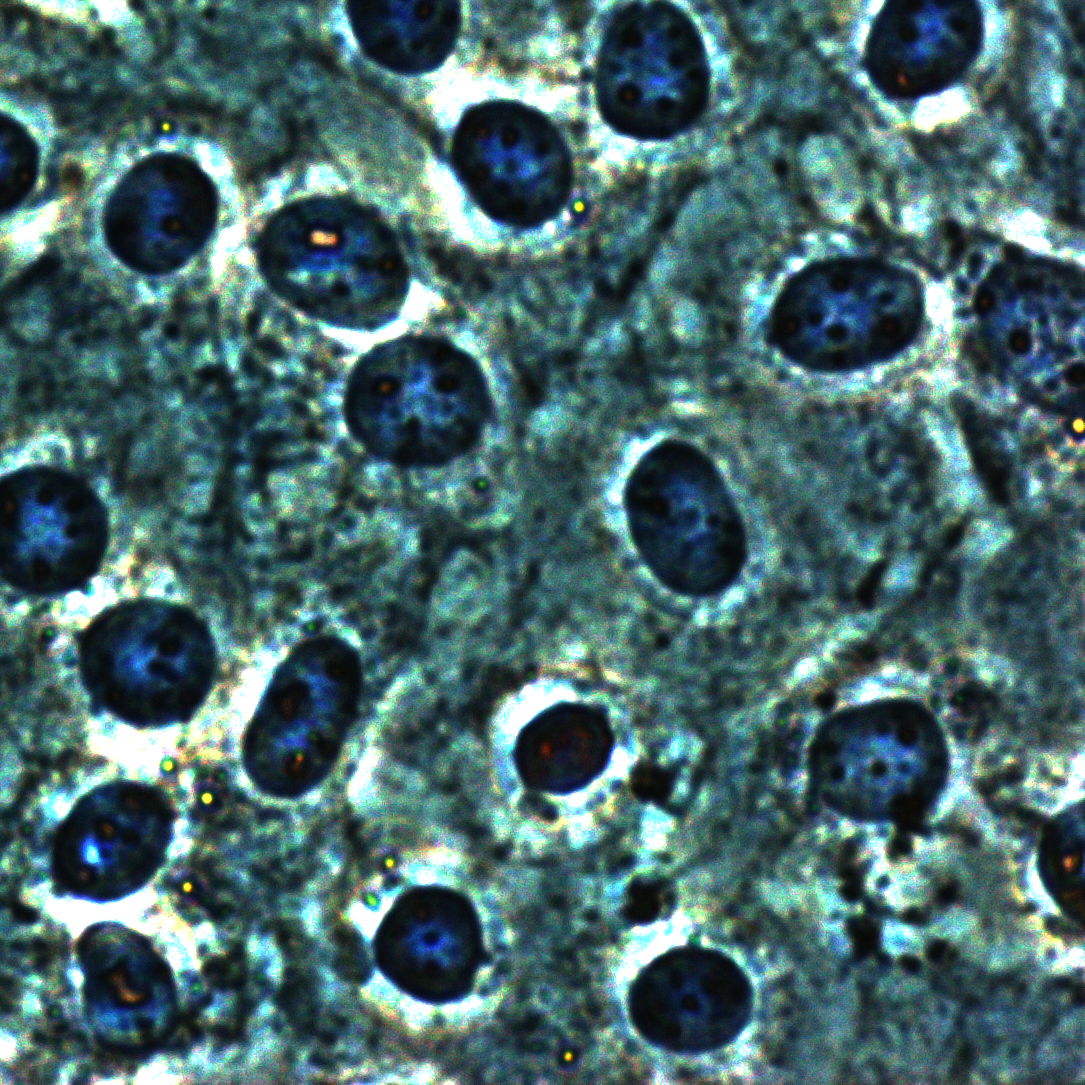

Supplement: Data S1. Raw experimental data generated in this study [file mmc1.zip › All original data/Morphological detection/Oil-red staining/Oil-red staining for Figure 4/Day0/Day0-2.tif]

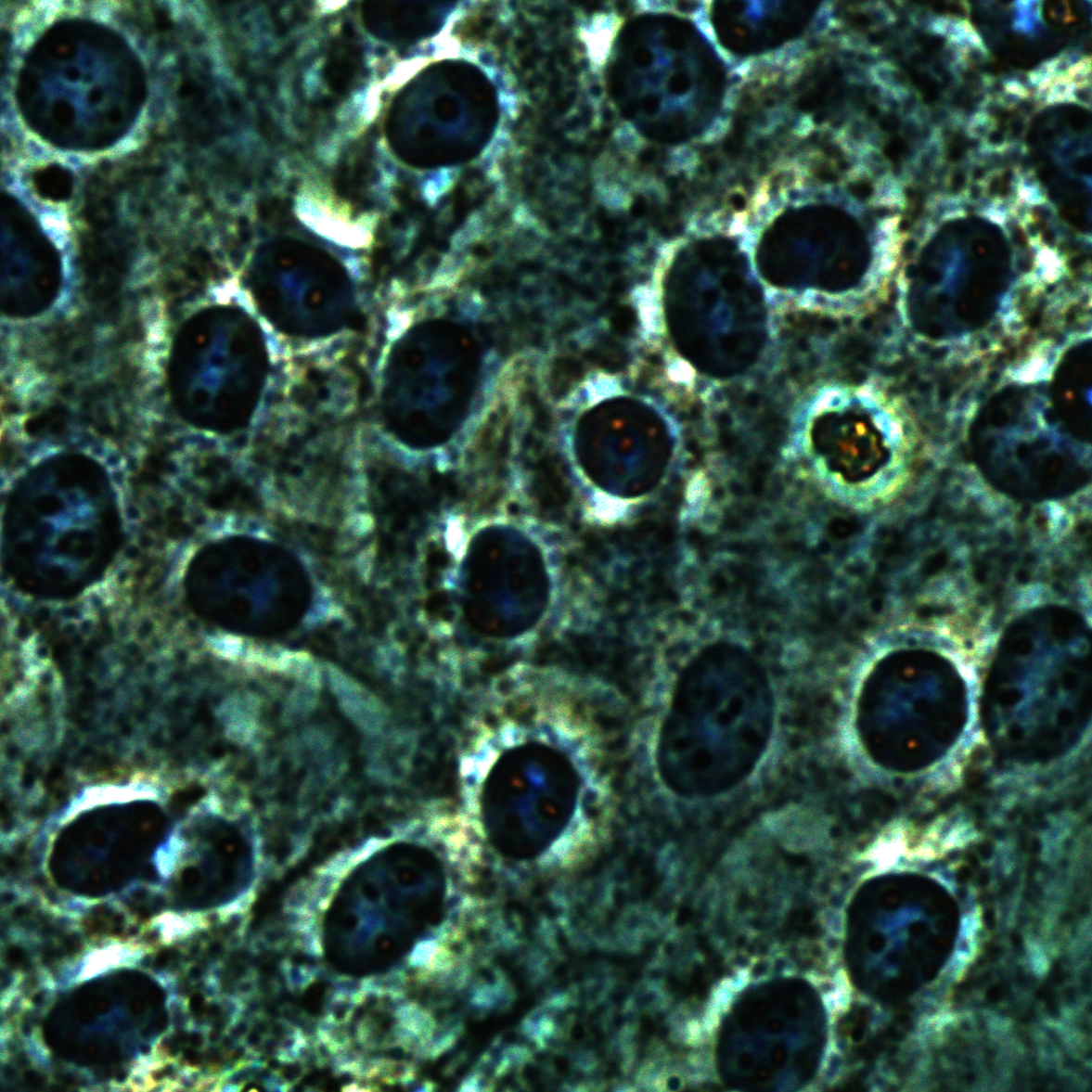

Supplement: Data S1. Raw experimental data generated in this study [file mmc1.zip › All original data/Morphological detection/Oil-red staining/Oil-red staining for Figure 4/Day0/Day0-3.tif]

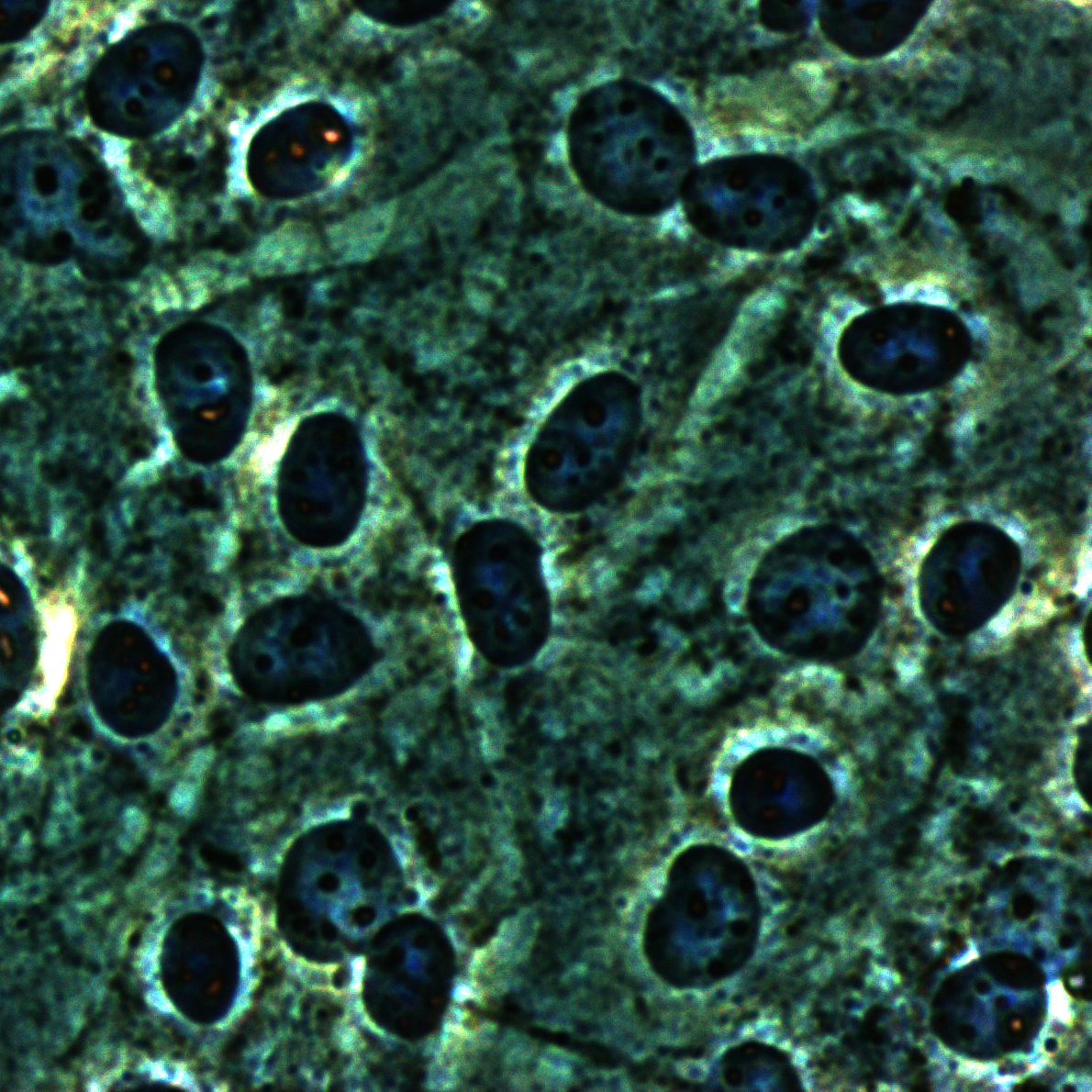

Supplement: Data S1. Raw experimental data generated in this study [file mmc1.zip › All original data/Morphological detection/Oil-red staining/Oil-red staining for Figure 4/Day0/Day0-4.tif]

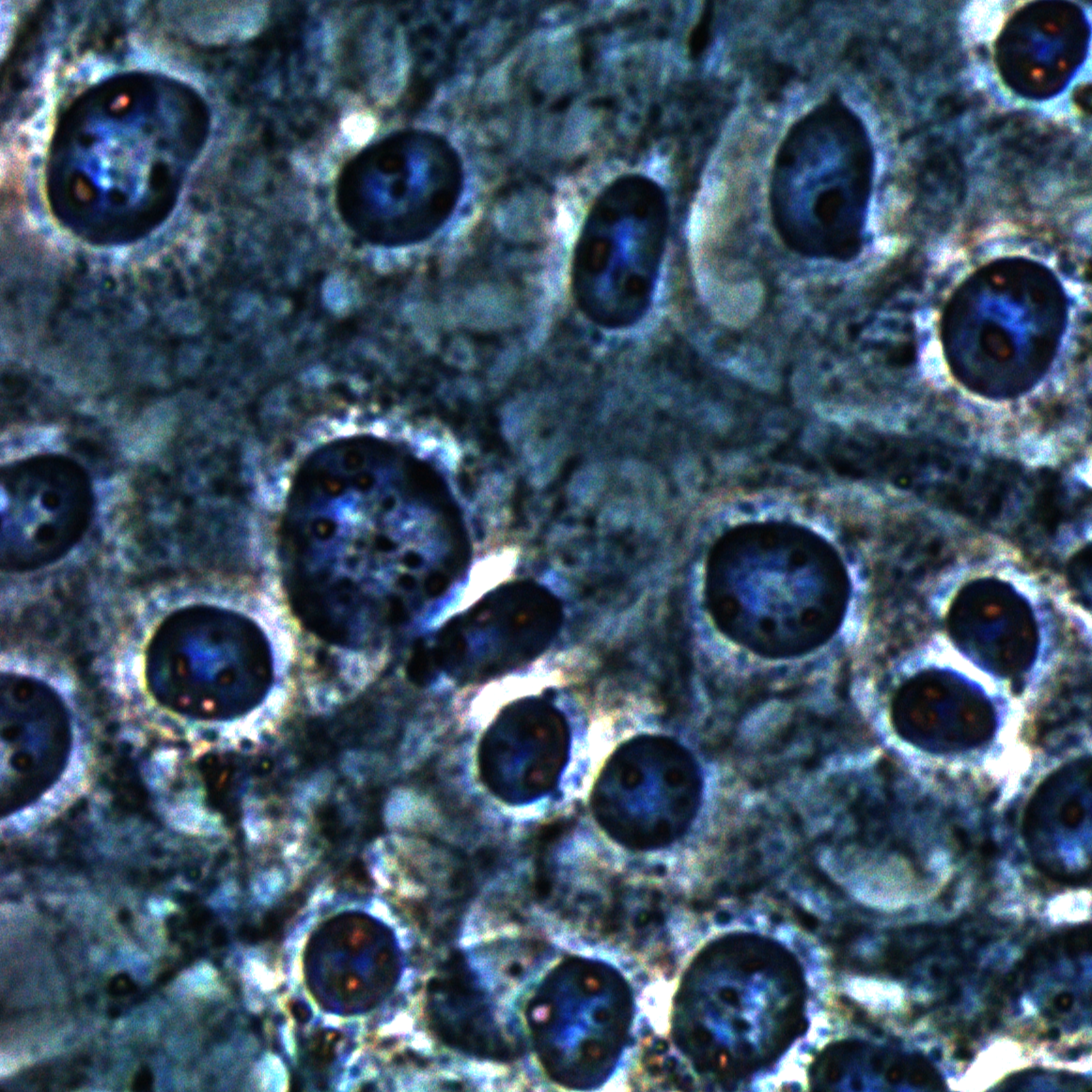

Supplement: Data S1. Raw experimental data generated in this study [file mmc1.zip › All original data/Morphological detection/Oil-red staining/Oil-red staining for Figure 4/Day0/Day0-5.tif]

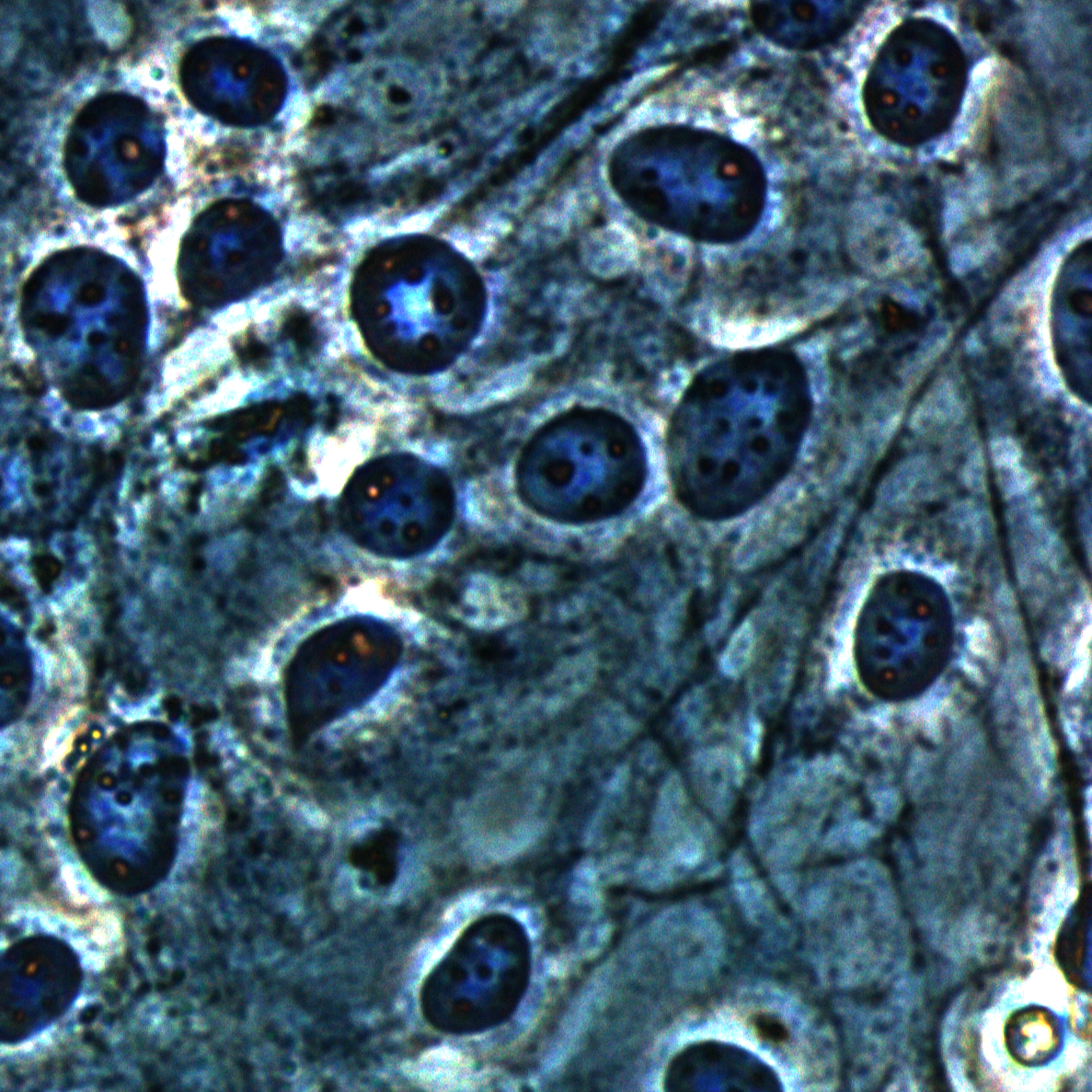

Supplement: Data S1. Raw experimental data generated in this study [file mmc1.zip › All original data/Morphological detection/Oil-red staining/Oil-red staining for Figure 4/Day0/Day0-6.tif]

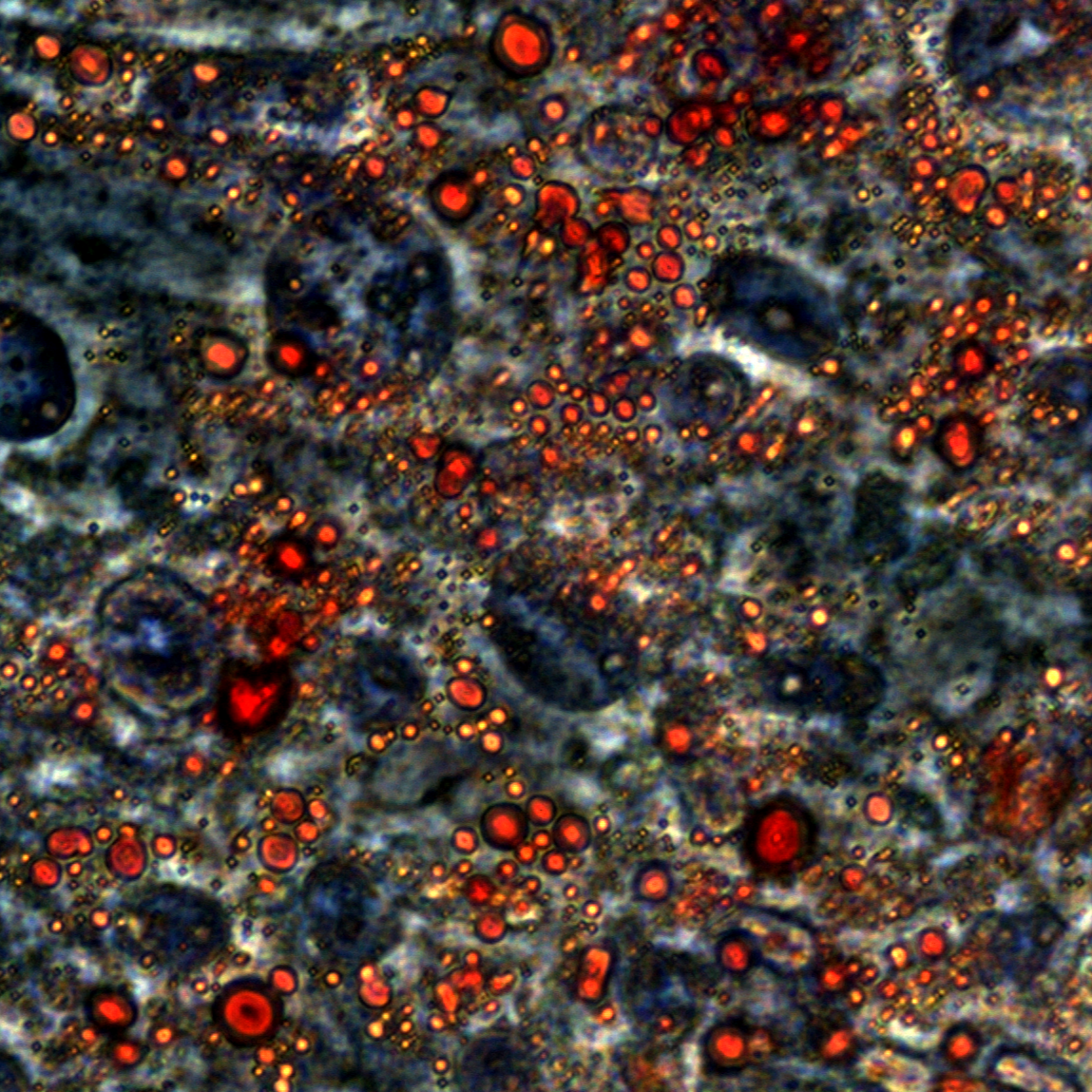

Supplement: Data S1. Raw experimental data generated in this study [file mmc1.zip › All original data/Morphological detection/Oil-red staining/Oil-red staining for Figure 4/Day10/Day10-1 used for manuscript.tif]

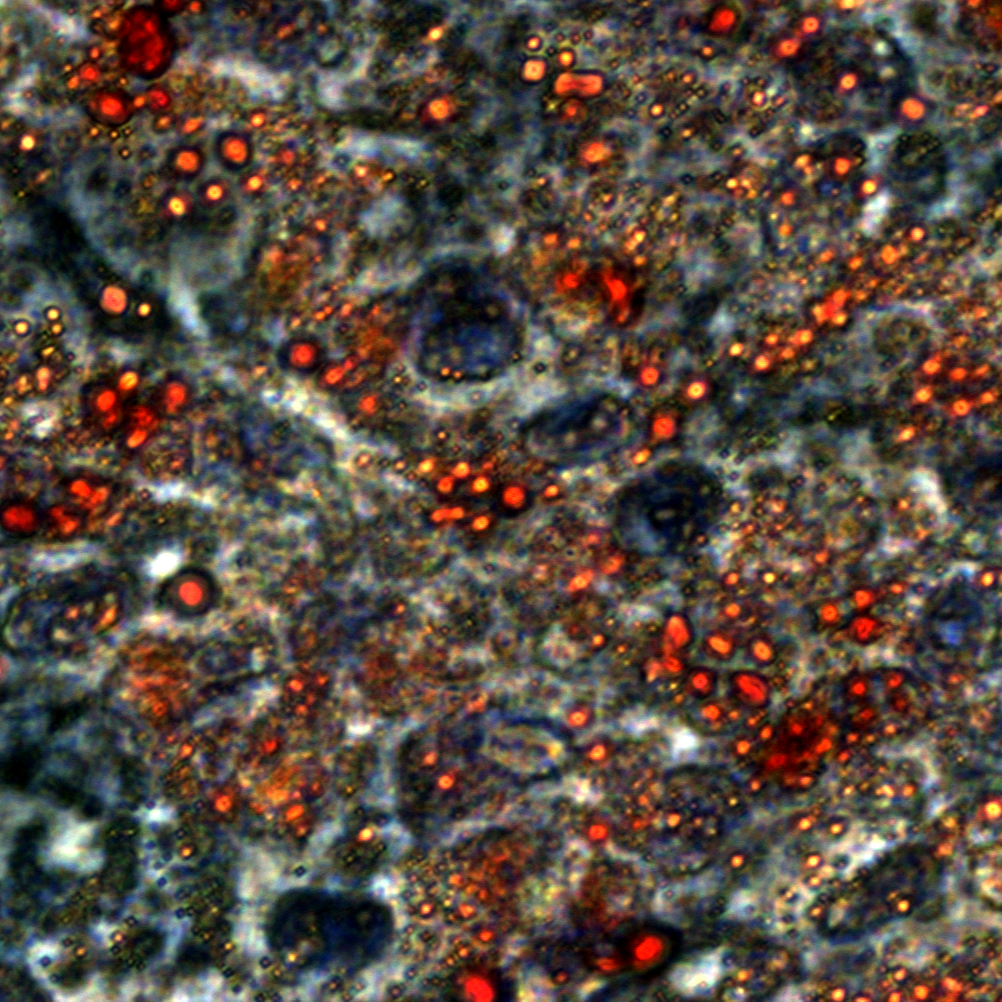

Supplement: Data S1. Raw experimental data generated in this study [file mmc1.zip › All original data/Morphological detection/Oil-red staining/Oil-red staining for Figure 4/Day10/Day10-2.tif]

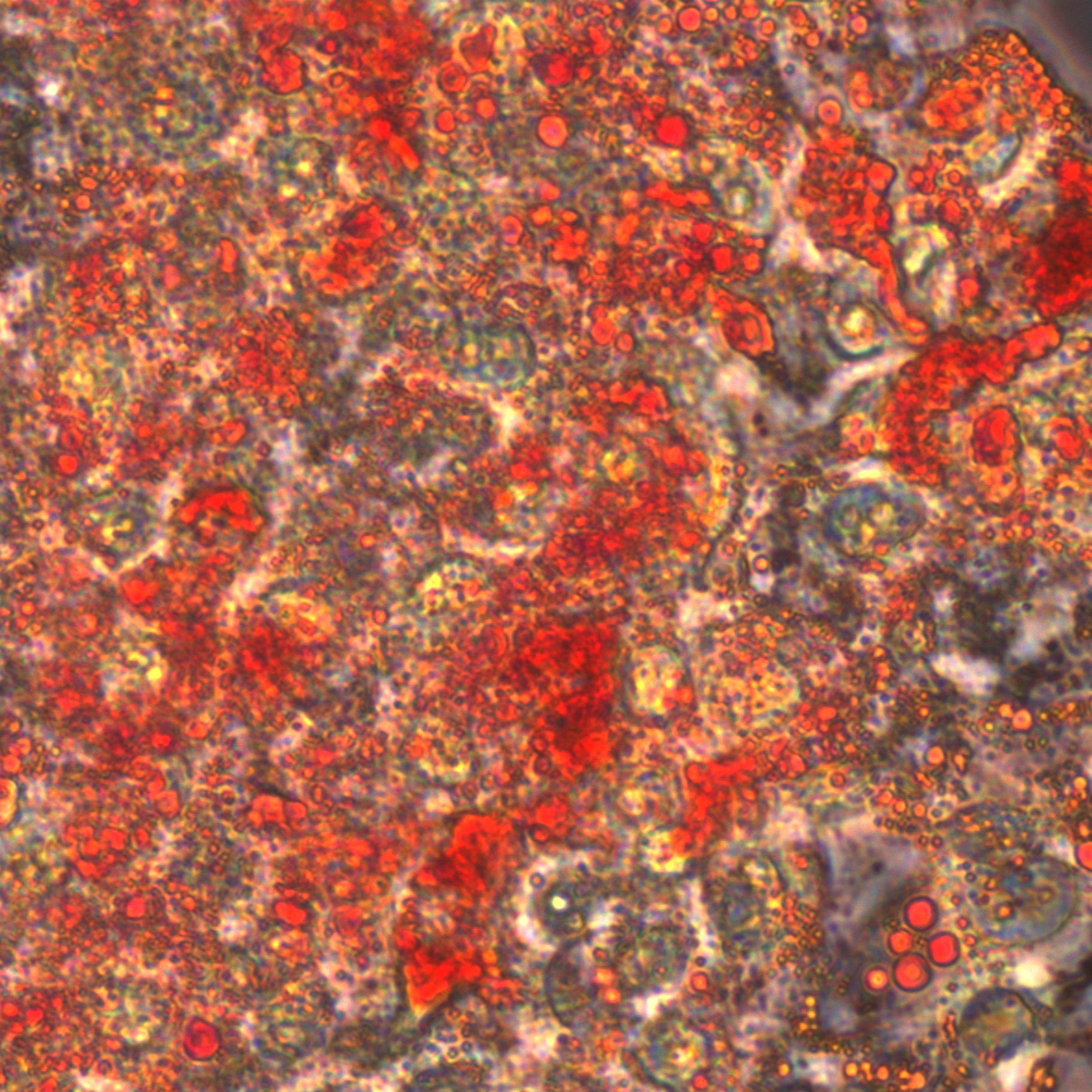

Supplement: Data S1. Raw experimental data generated in this study [file mmc1.zip › All original data/Morphological detection/Oil-red staining/Oil-red staining for Figure 4/Day10/Day10-3.tif]

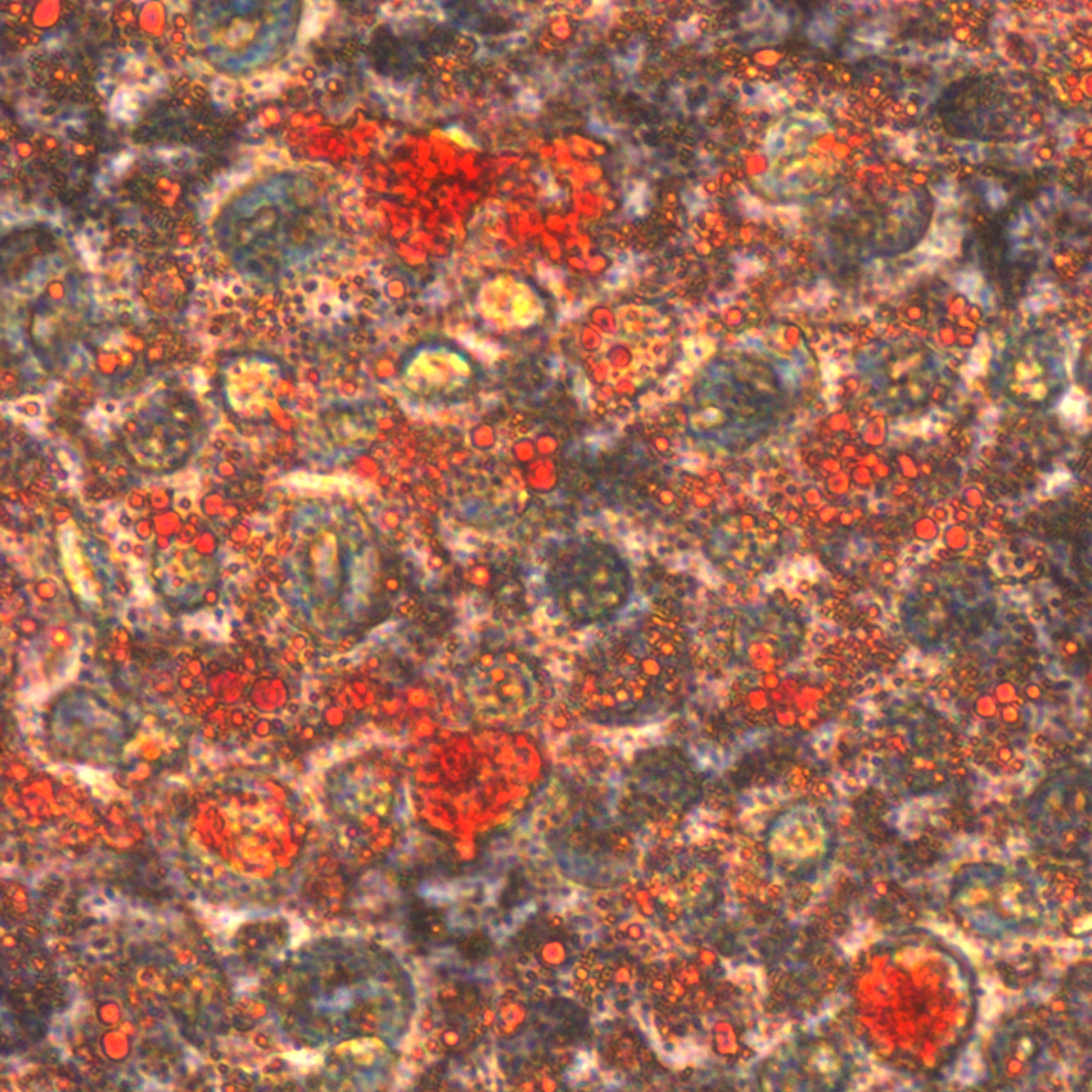

Supplement: Data S1. Raw experimental data generated in this study [file mmc1.zip › All original data/Morphological detection/Oil-red staining/Oil-red staining for Figure 4/Day10/Day10-4.tif]

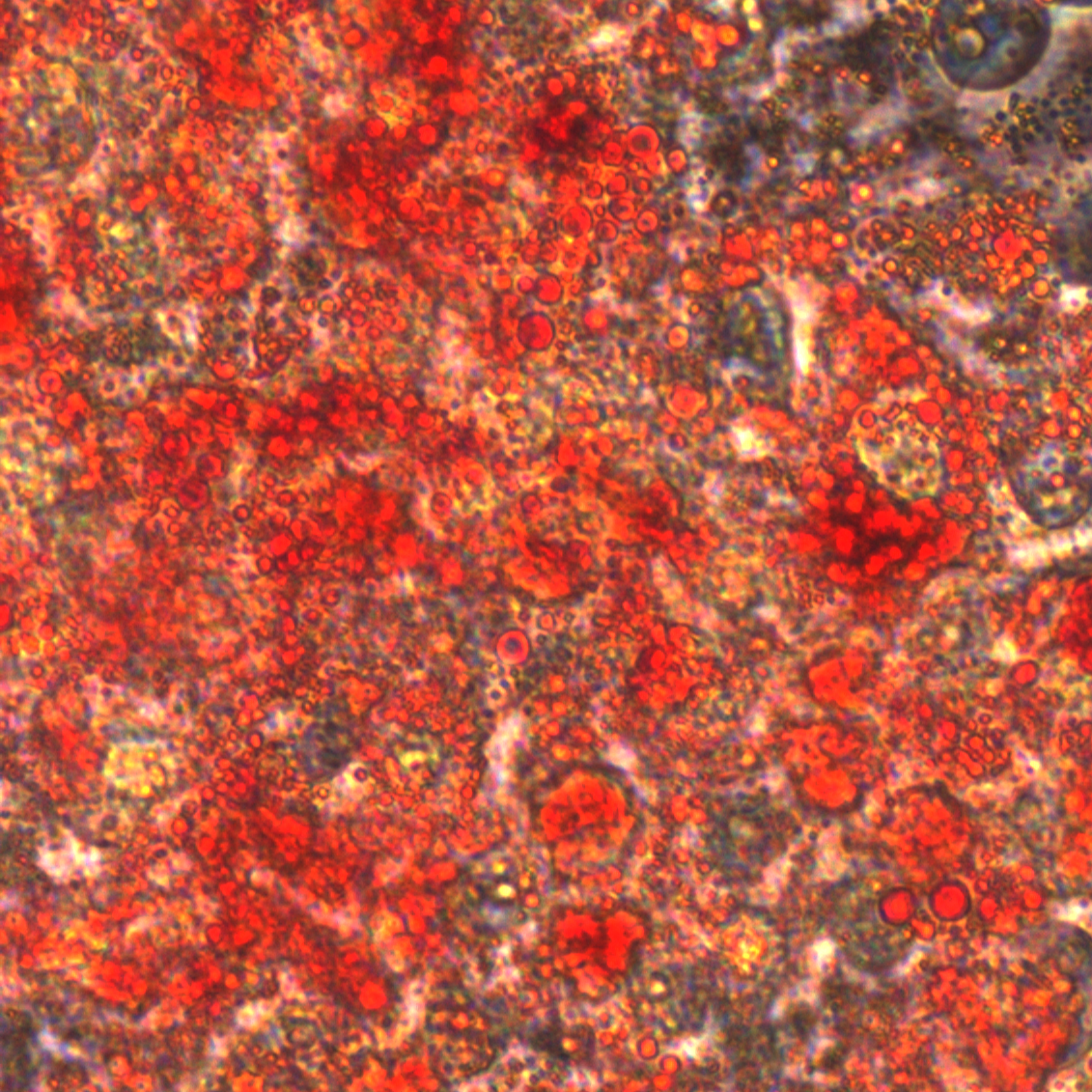

Supplement: Data S1. Raw experimental data generated in this study [file mmc1.zip › All original data/Morphological detection/Oil-red staining/Oil-red staining for Figure 4/Day10/Day10-5.tif]

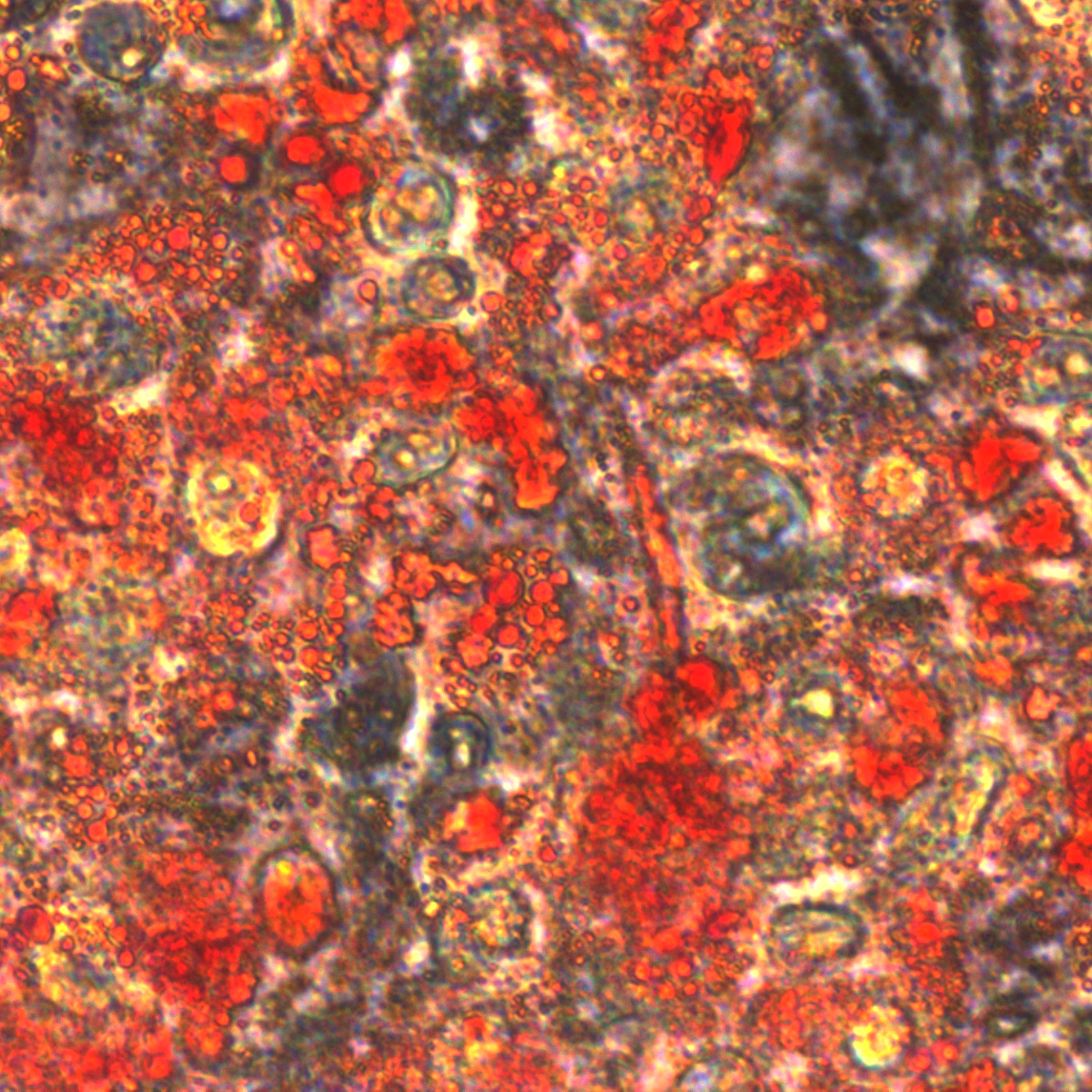

Supplement: Data S1. Raw experimental data generated in this study [file mmc1.zip › All original data/Morphological detection/Oil-red staining/Oil-red staining for Figure 4/Day10/Day10-6.tif]

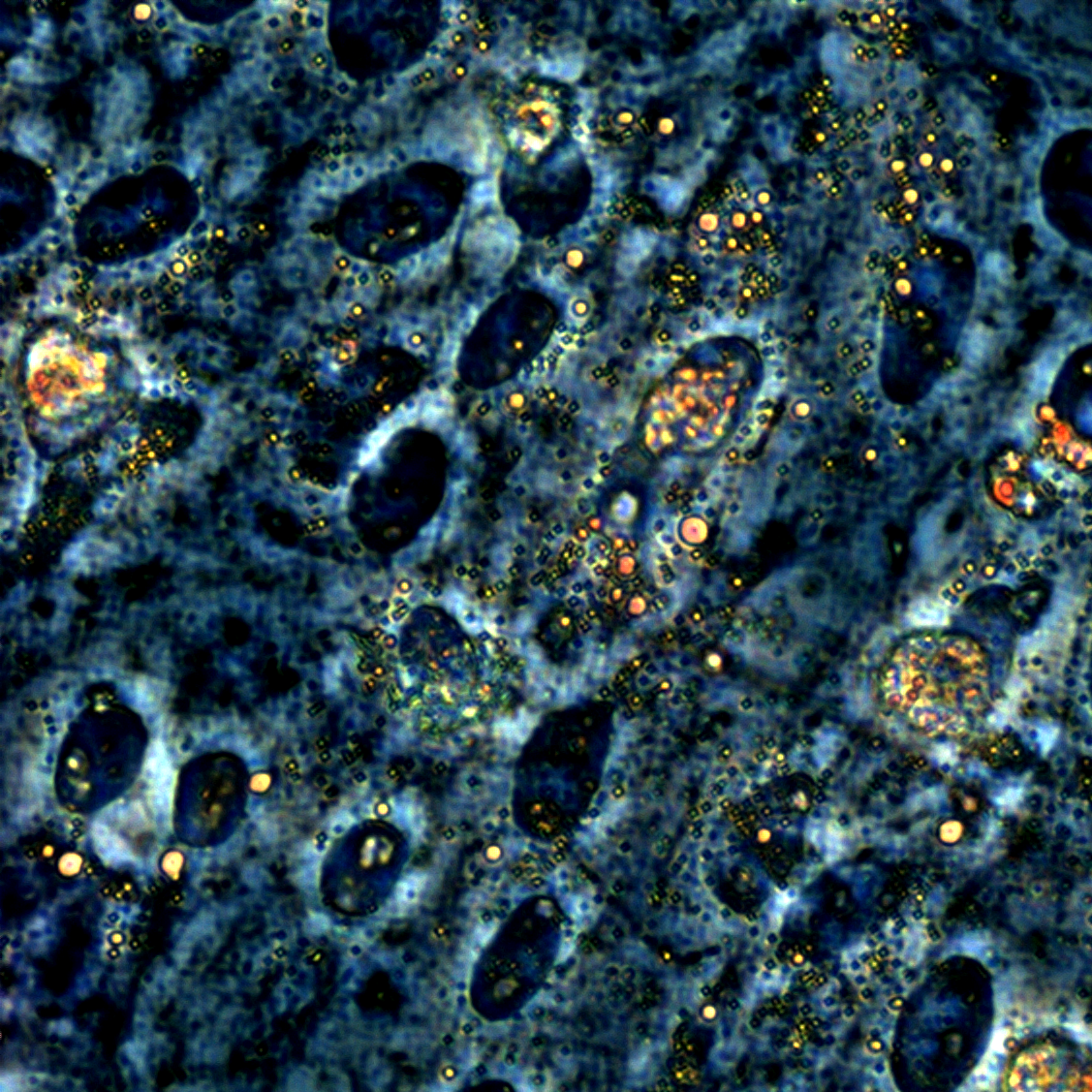

Supplement: Data S1. Raw experimental data generated in this study [file mmc1.zip › All original data/Morphological detection/Oil-red staining/Oil-red staining for Figure 4/Day4/Day4-1 used for manuscript.tif]

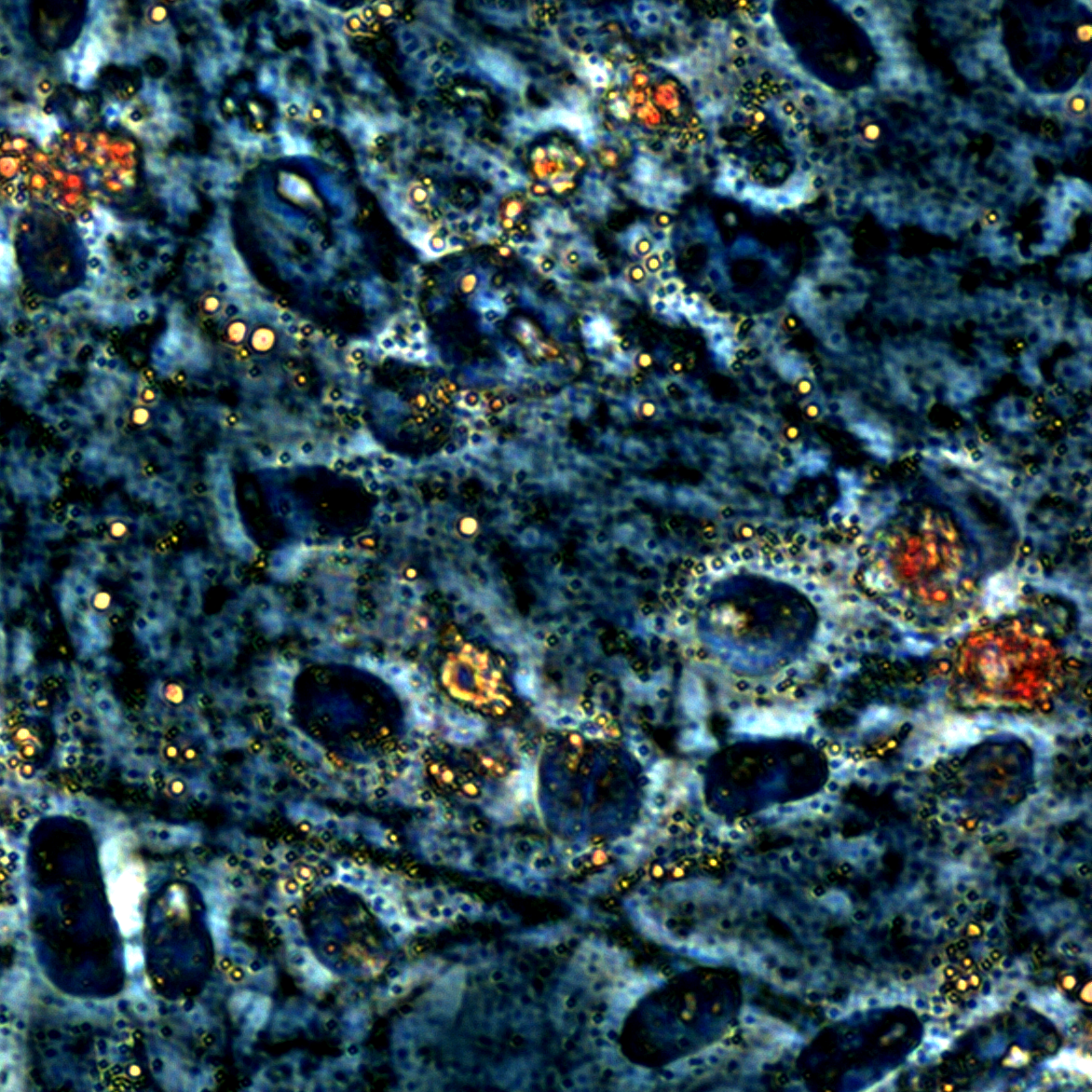

Supplement: Data S1. Raw experimental data generated in this study [file mmc1.zip › All original data/Morphological detection/Oil-red staining/Oil-red staining for Figure 4/Day4/Day4-2.tif]

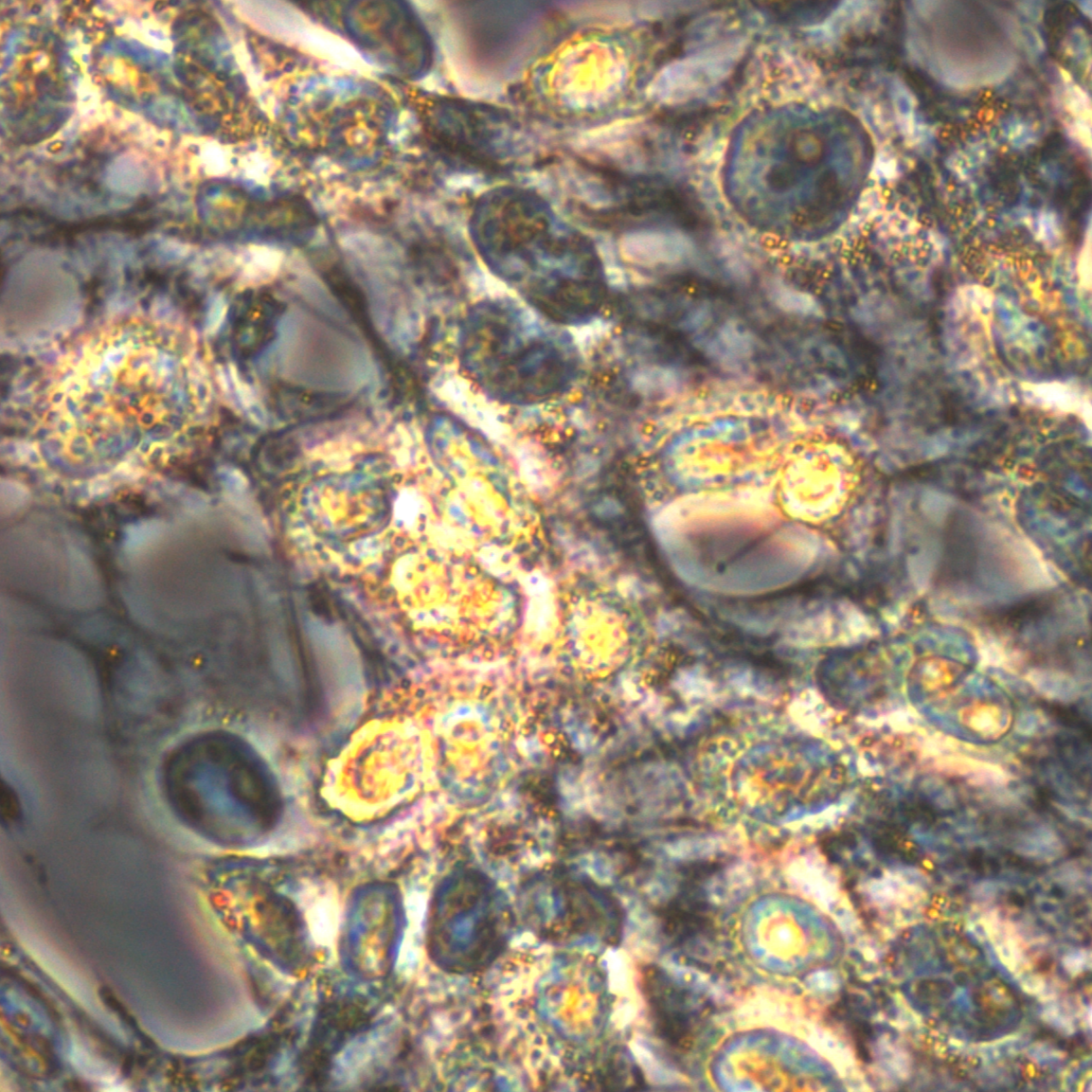

Supplement: Data S1. Raw experimental data generated in this study [file mmc1.zip › All original data/Morphological detection/Oil-red staining/Oil-red staining for Figure 4/Day4/Day4-3.tif]

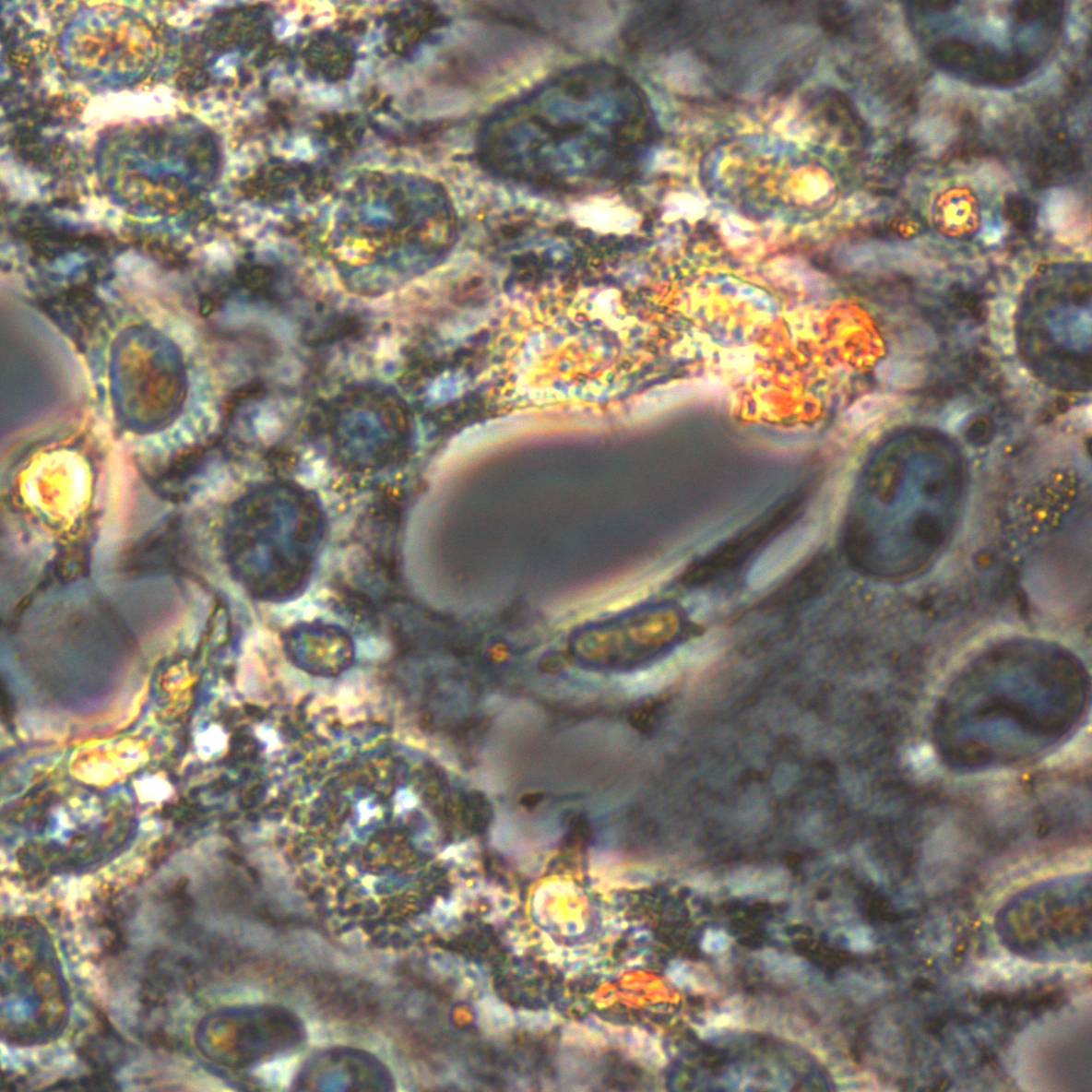

Supplement: Data S1. Raw experimental data generated in this study [file mmc1.zip › All original data/Morphological detection/Oil-red staining/Oil-red staining for Figure 4/Day4/Day4-4.tif]

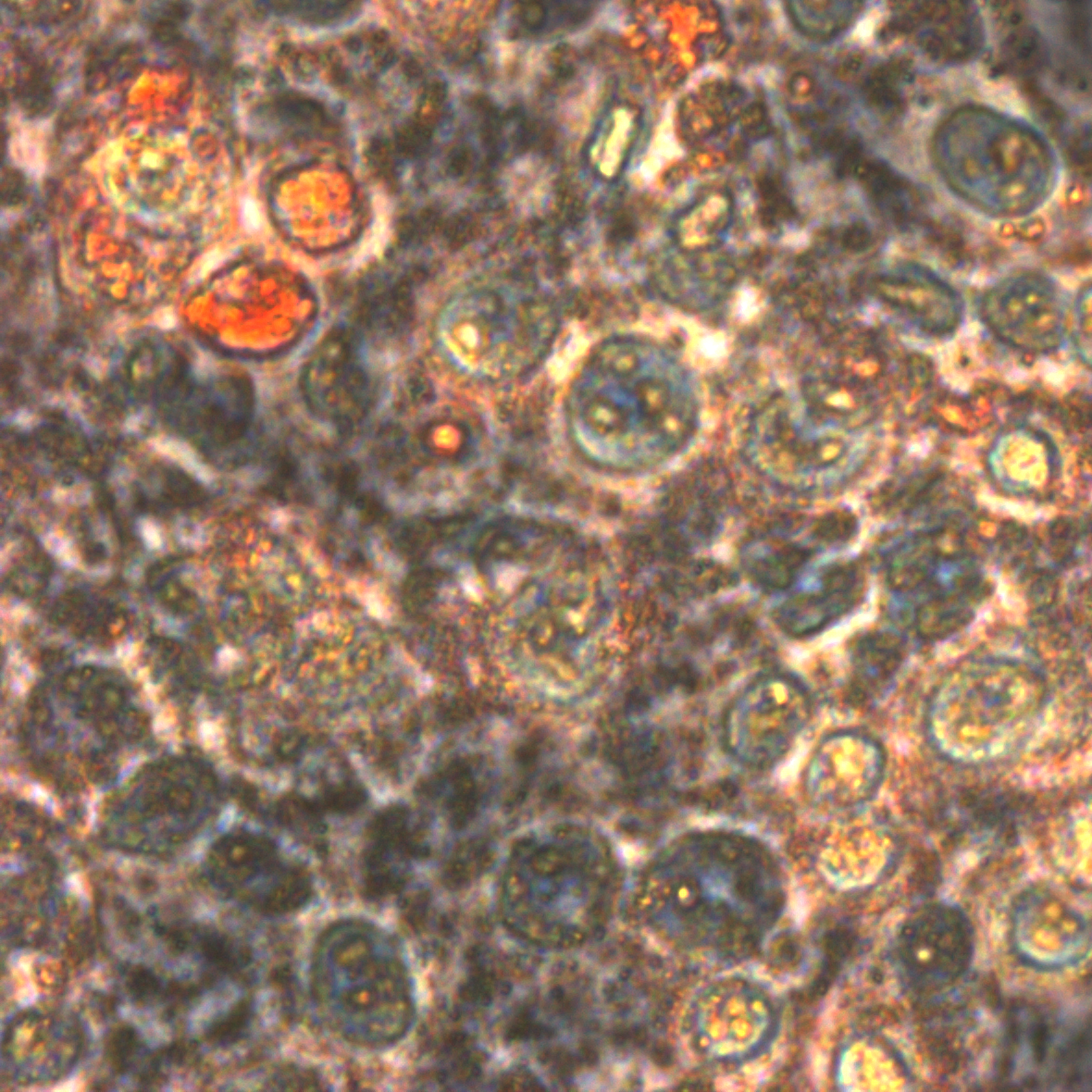

Supplement: Data S1. Raw experimental data generated in this study [file mmc1.zip › All original data/Morphological detection/Oil-red staining/Oil-red staining for Figure 4/Day4/Day4-5.tif]

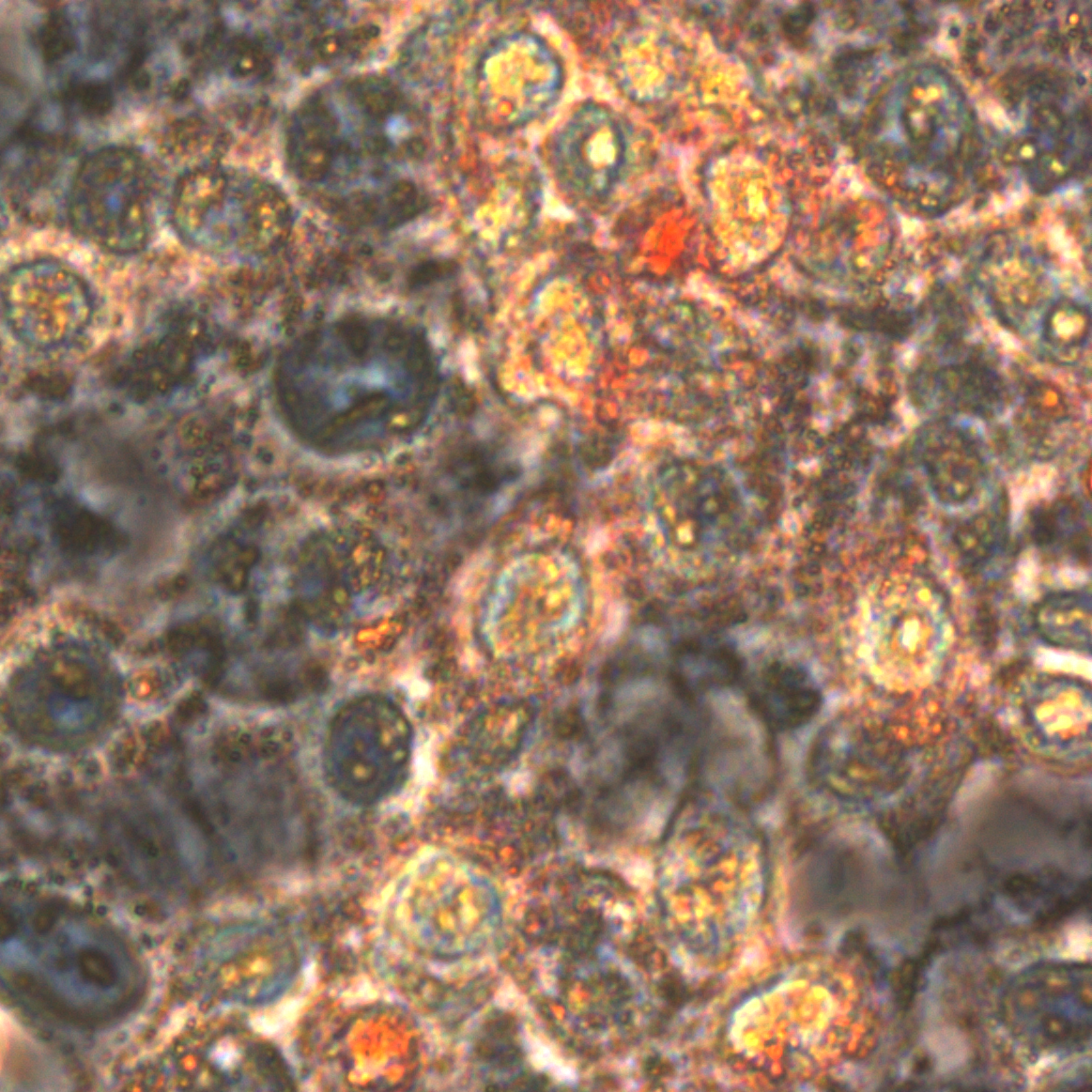

Supplement: Data S1. Raw experimental data generated in this study [file mmc1.zip › All original data/Morphological detection/Oil-red staining/Oil-red staining for Figure 4/Day4/Day4-6.tif]

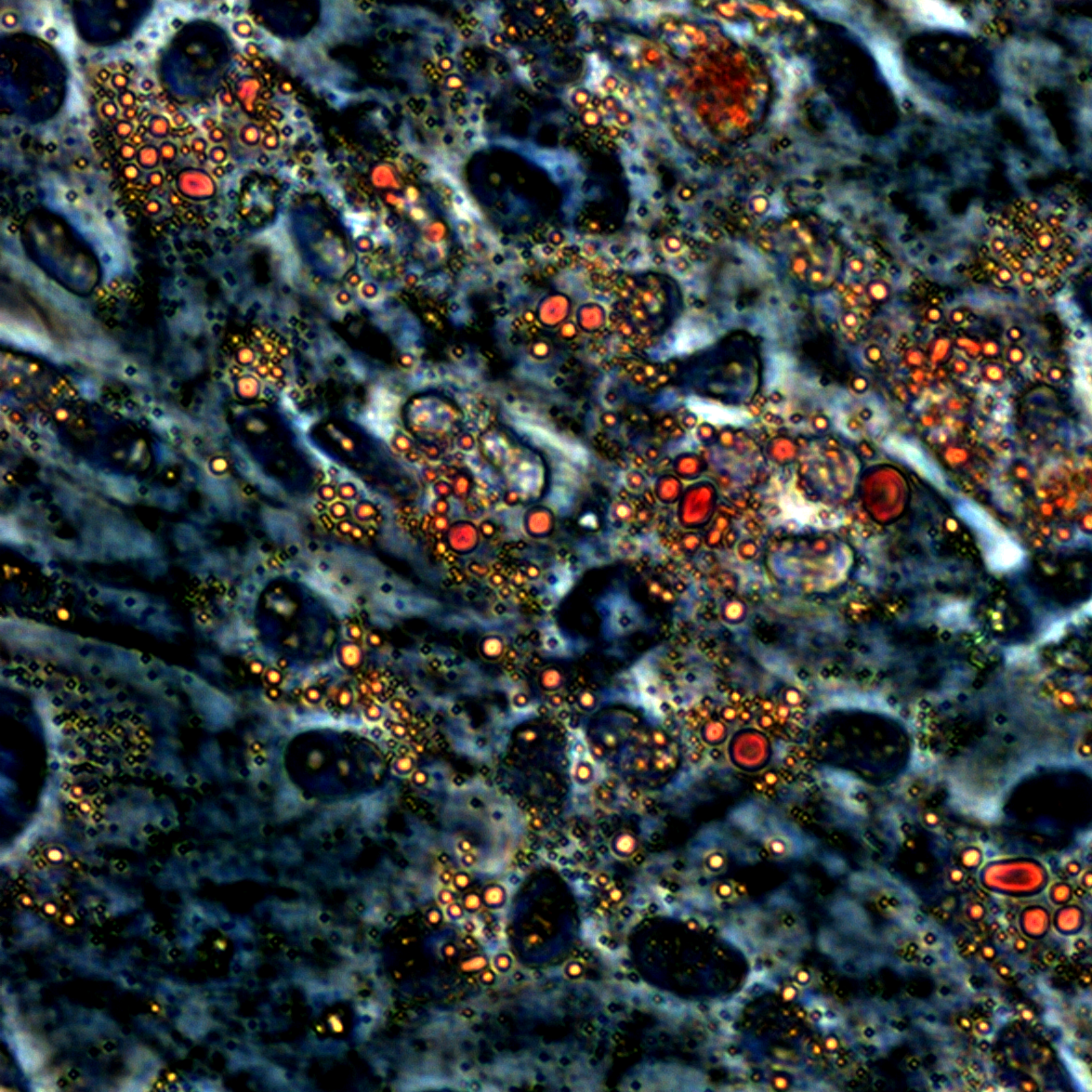

Supplement: Data S1. Raw experimental data generated in this study [file mmc1.zip › All original data/Morphological detection/Oil-red staining/Oil-red staining for Figure 4/Day8/Day8-1 used for manuscript.tif]

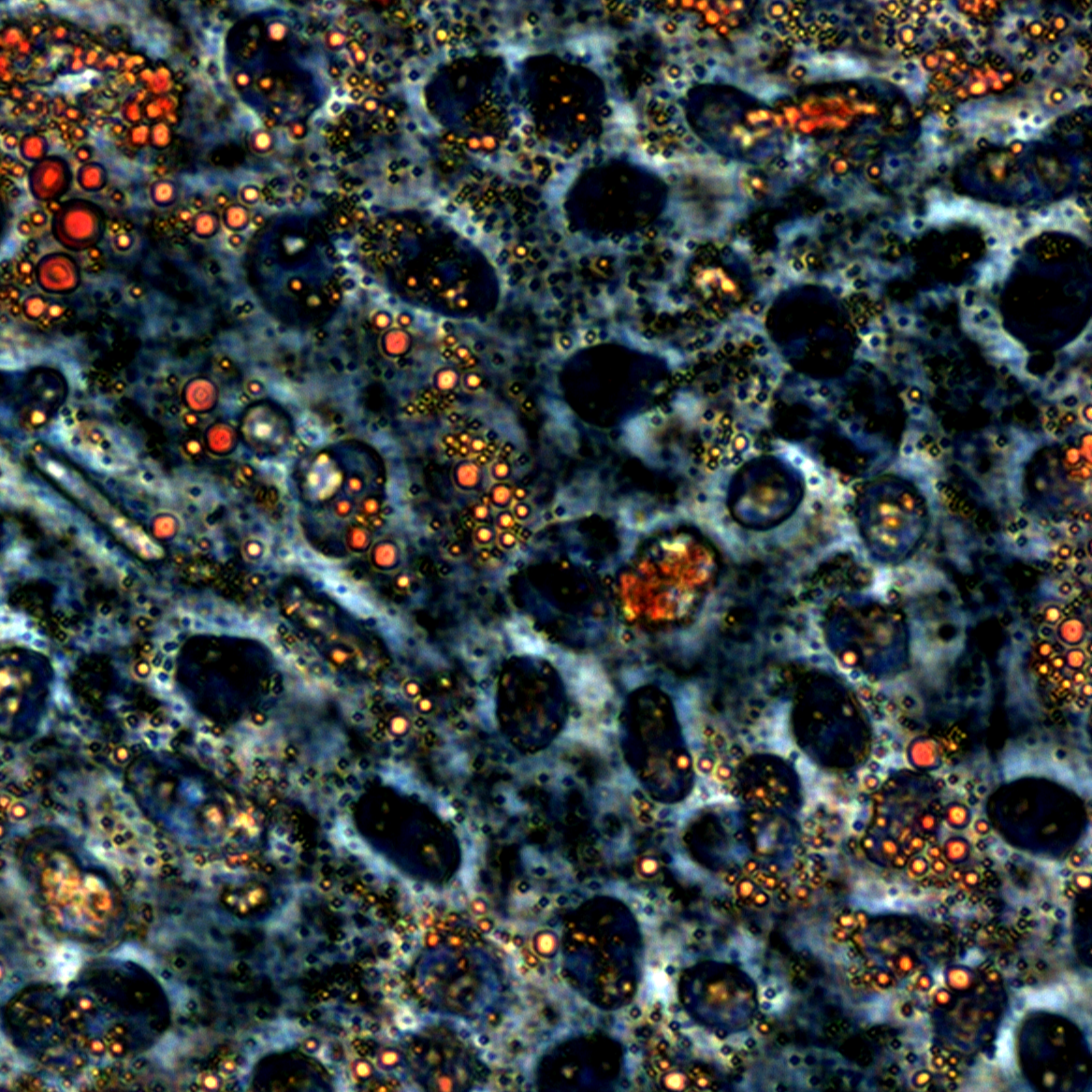

Supplement: Data S1. Raw experimental data generated in this study [file mmc1.zip › All original data/Morphological detection/Oil-red staining/Oil-red staining for Figure 4/Day8/Day8-2.tif]

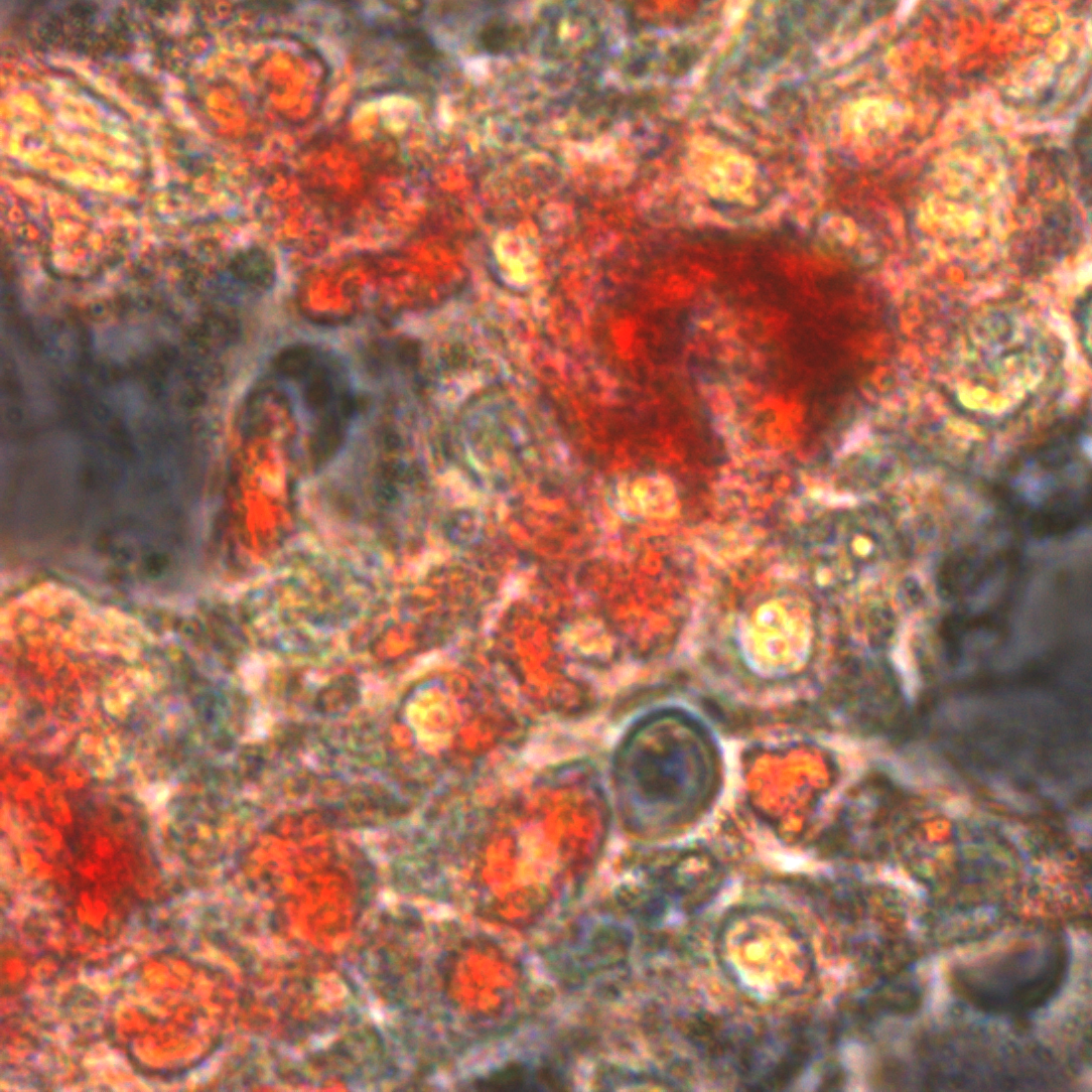

Supplement: Data S1. Raw experimental data generated in this study [file mmc1.zip › All original data/Morphological detection/Oil-red staining/Oil-red staining for Figure 4/Day8/Day8-3.tif]

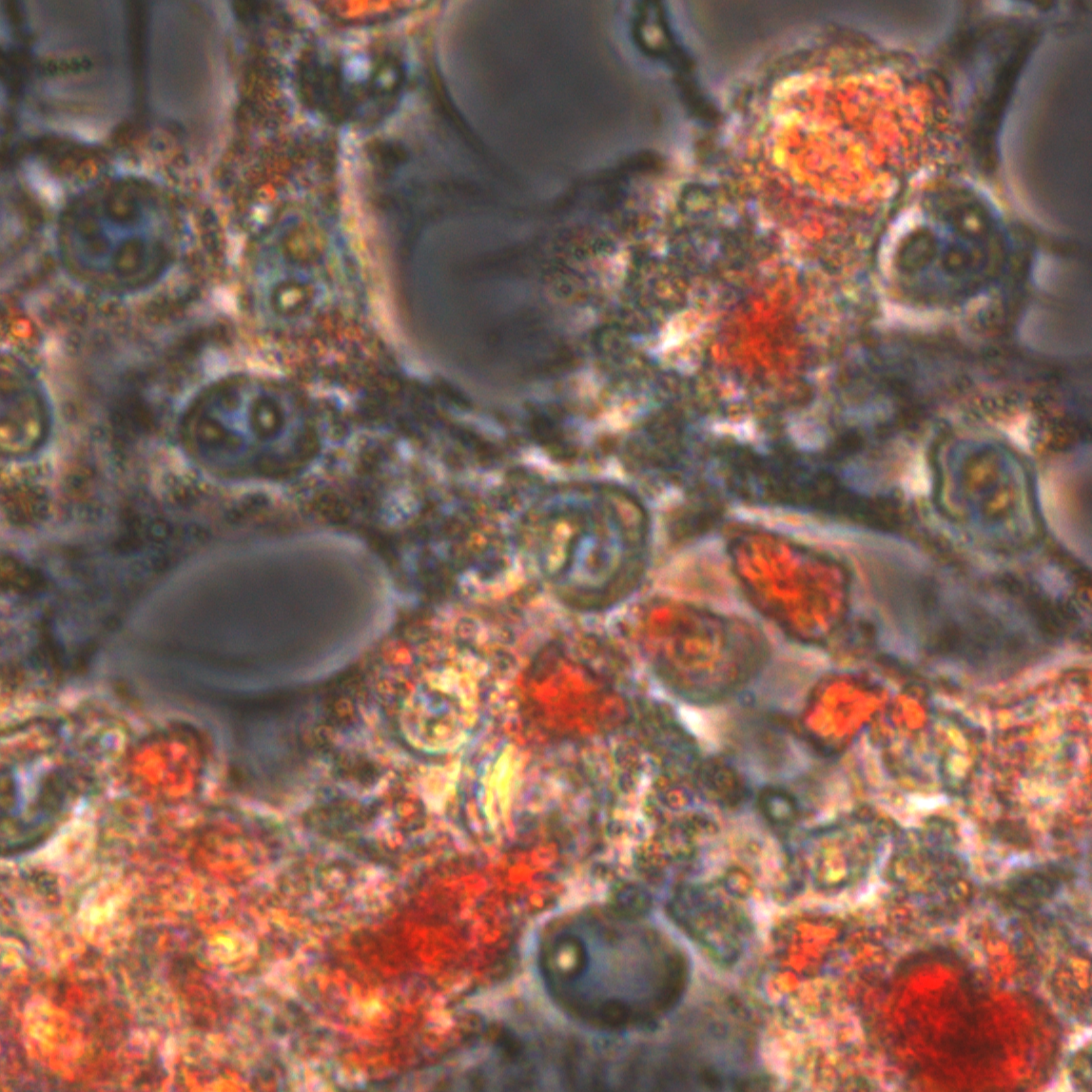

Supplement: Data S1. Raw experimental data generated in this study [file mmc1.zip › All original data/Morphological detection/Oil-red staining/Oil-red staining for Figure 4/Day8/Day8-4.tif]

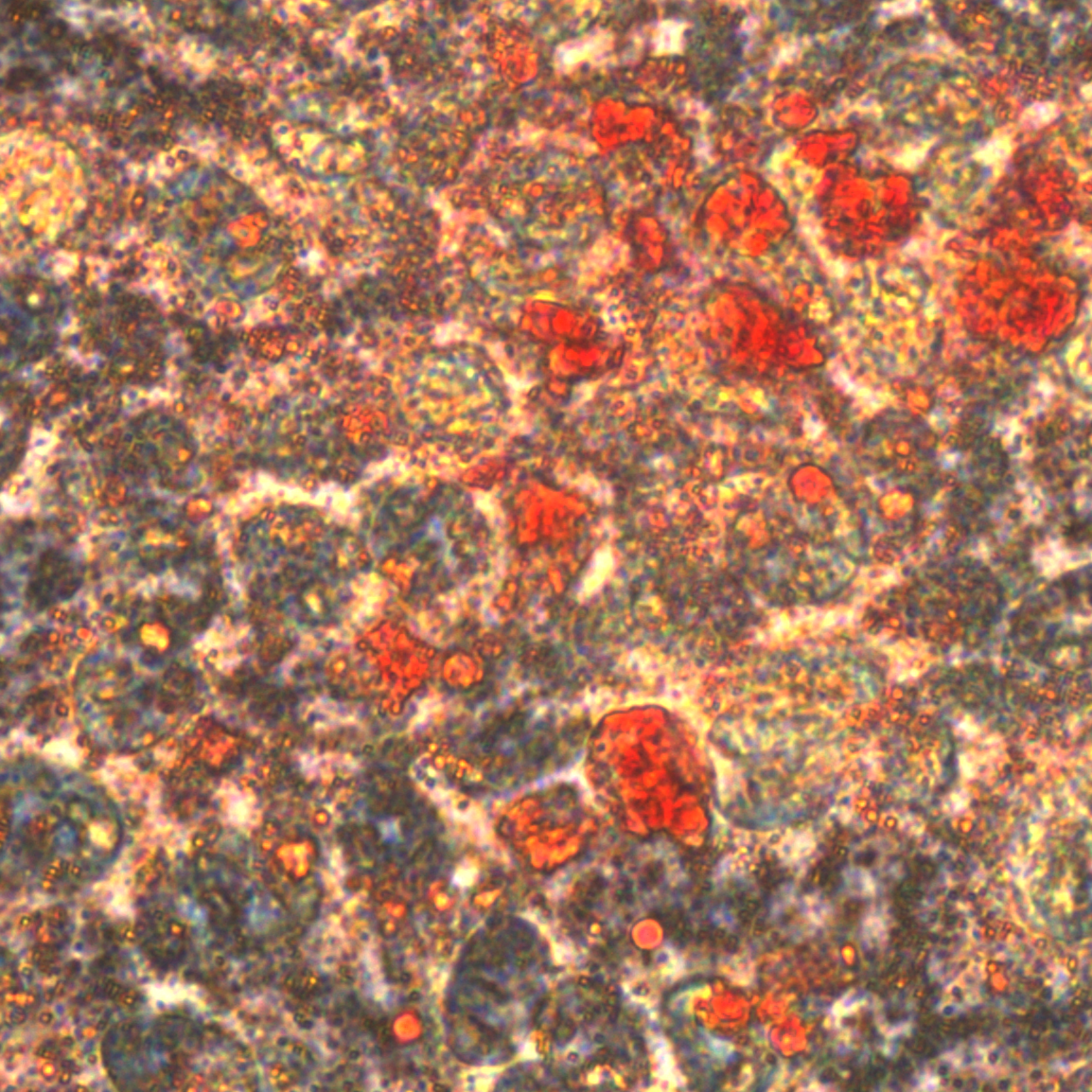

Supplement: Data S1. Raw experimental data generated in this study [file mmc1.zip › All original data/Morphological detection/Oil-red staining/Oil-red staining for Figure 4/Day8/Day8-5.tif]

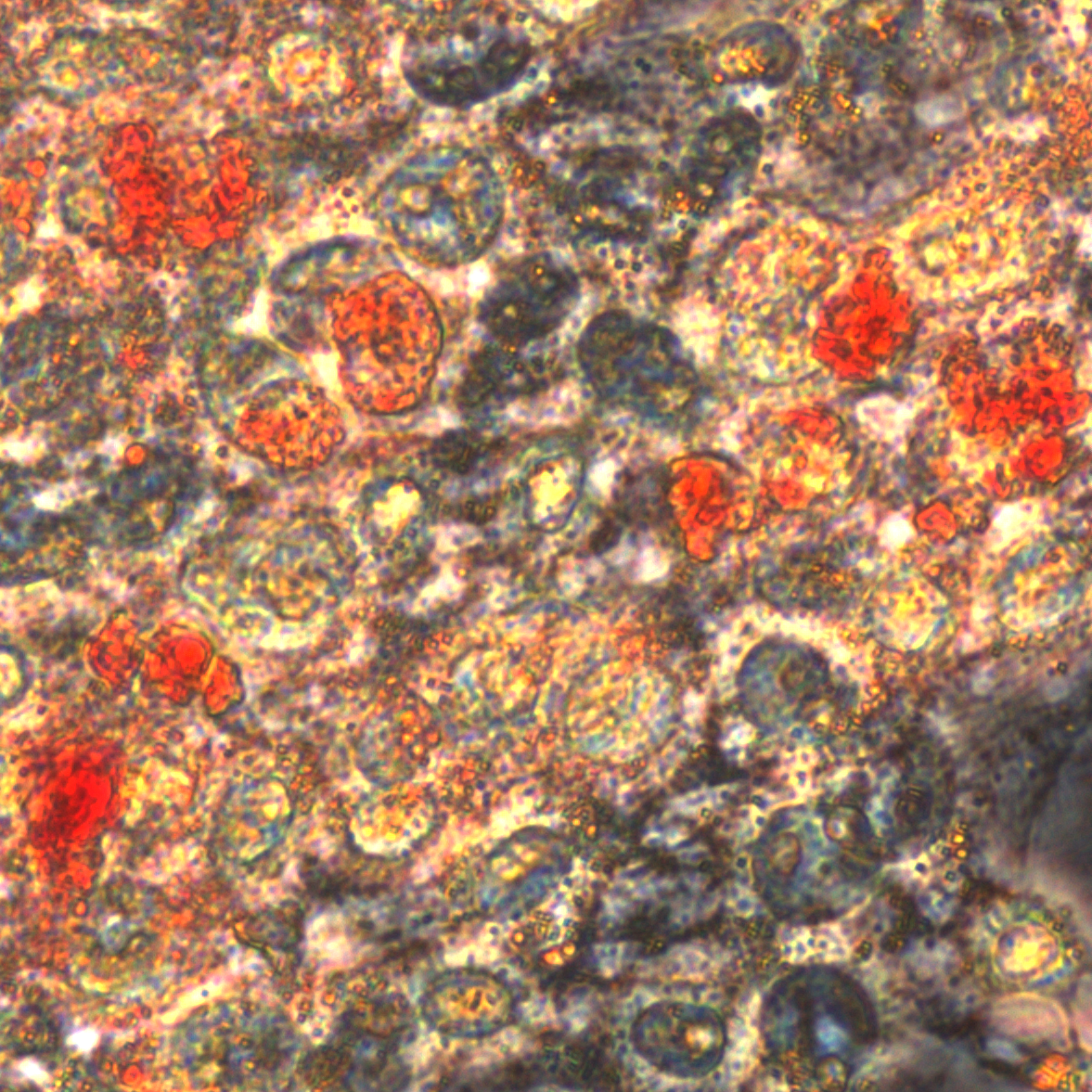

Supplement: Data S1. Raw experimental data generated in this study [file mmc1.zip › All original data/Morphological detection/Oil-red staining/Oil-red staining for Figure 4/Day8/Day8-6.tif]

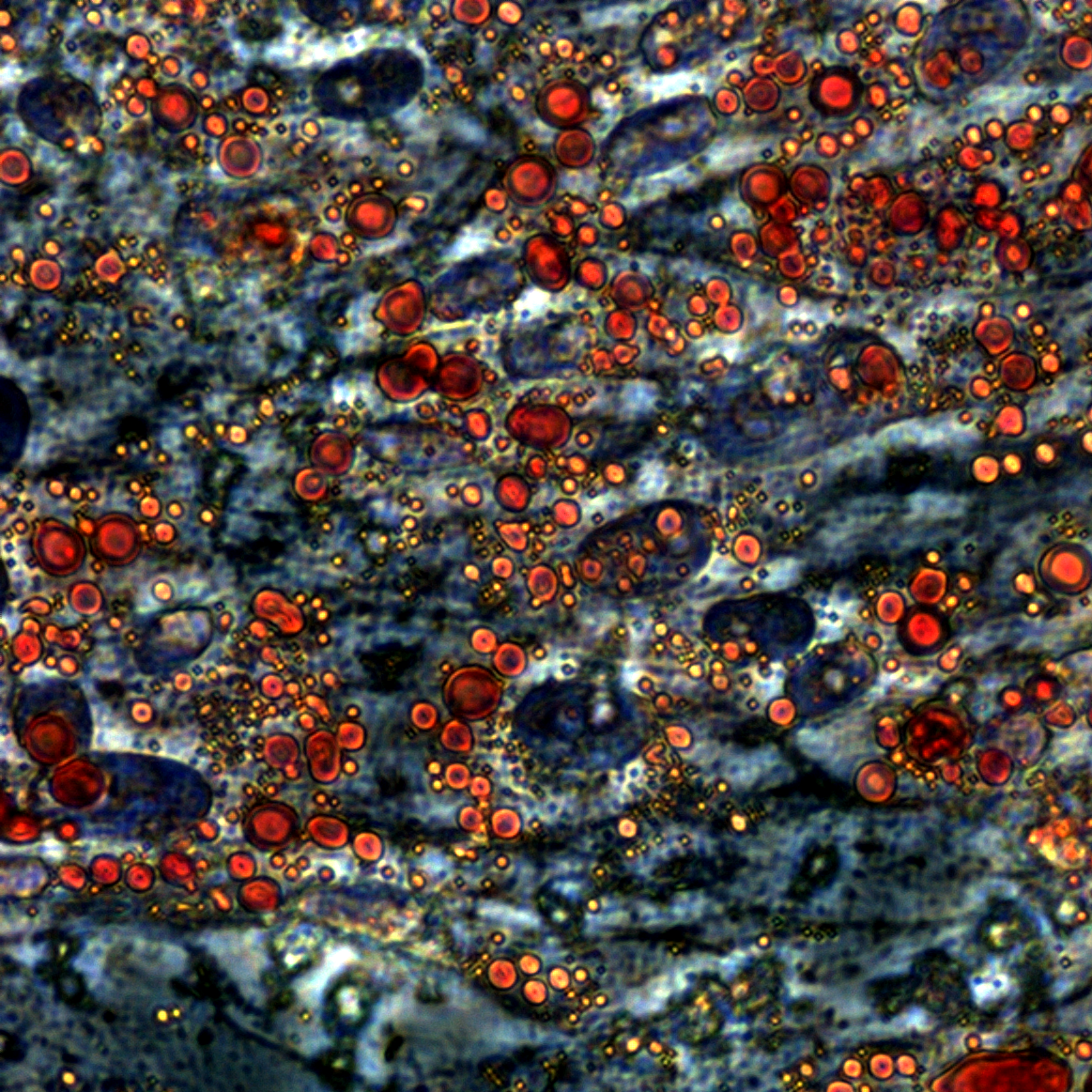

Supplement: Data S1. Raw experimental data generated in this study [file mmc1.zip › All original data/Morphological detection/Oil-red staining/Oil-red staining for Figure 5/miR-21a-5p Inhibitor/miR-21a-5p Inhibitor-1 used for manuscript.tif]

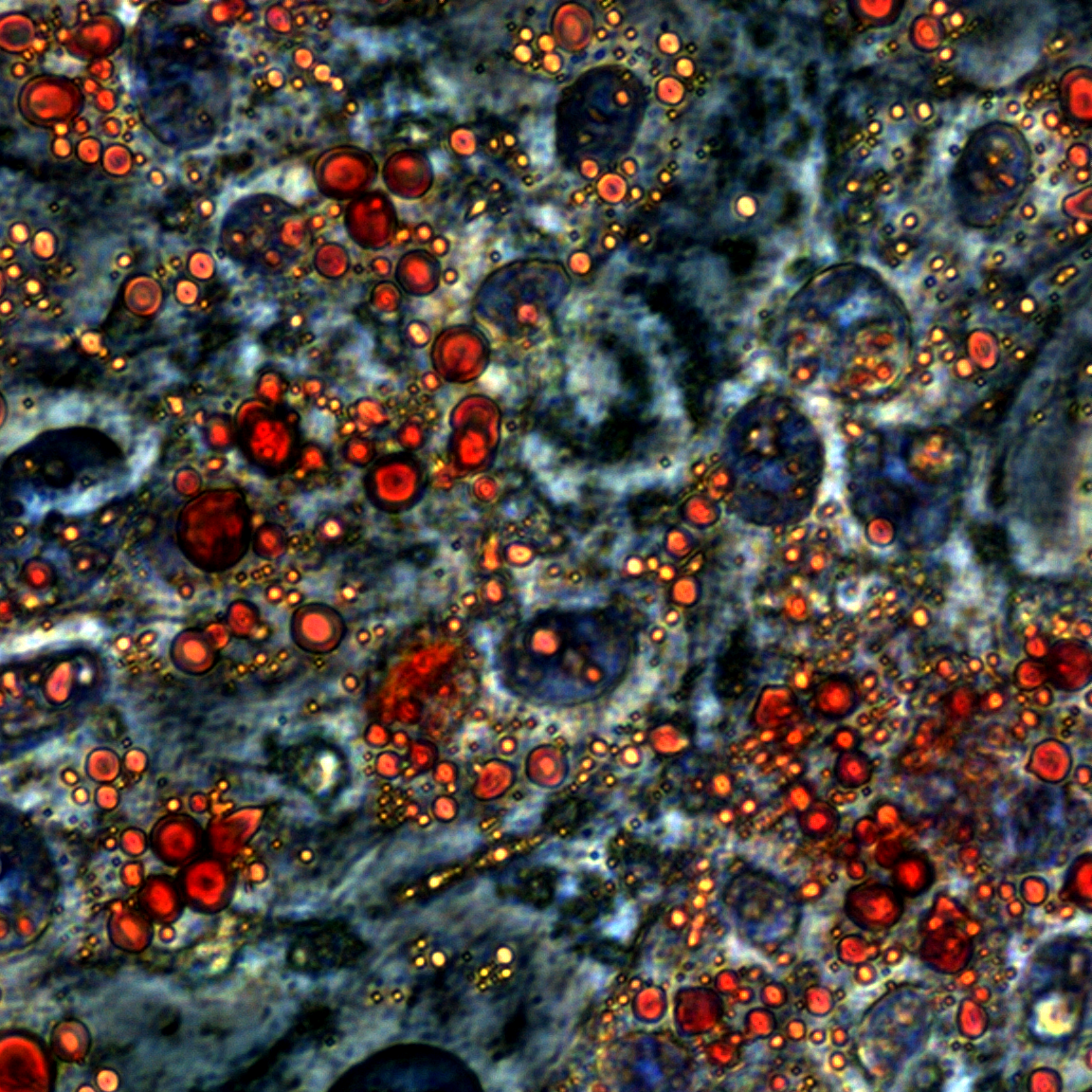

Supplement: Data S1. Raw experimental data generated in this study [file mmc1.zip › All original data/Morphological detection/Oil-red staining/Oil-red staining for Figure 5/miR-21a-5p Inhibitor/miR-21a-5p Inhibitor-2.tif]

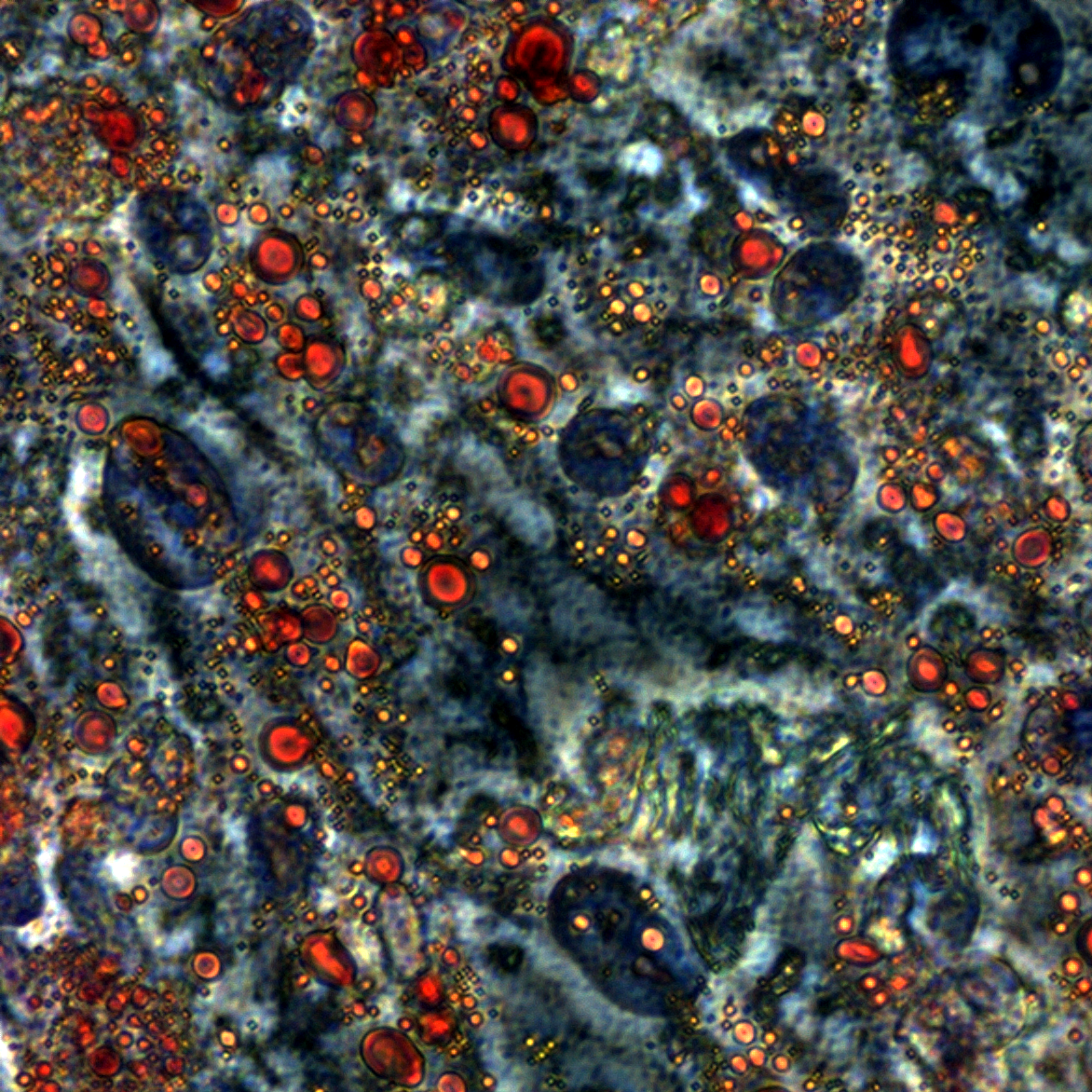

Supplement: Data S1. Raw experimental data generated in this study [file mmc1.zip › All original data/Morphological detection/Oil-red staining/Oil-red staining for Figure 5/miR-21a-5p Inhibitor/miR-21a-5p Inhibitor-3.tif]

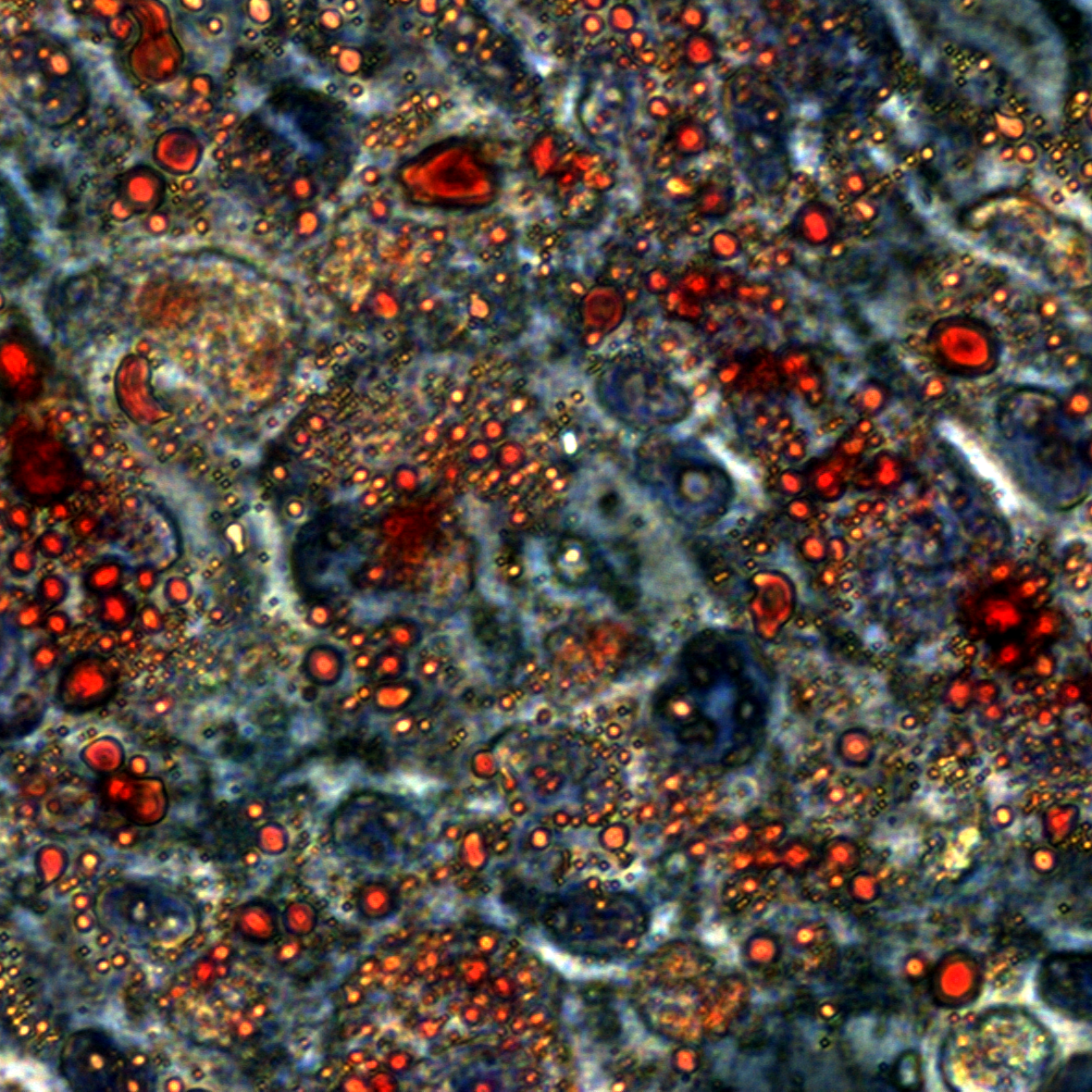

Supplement: Data S1. Raw experimental data generated in this study [file mmc1.zip › All original data/Morphological detection/Oil-red staining/Oil-red staining for Figure 5/miR-21a-5p Inhibitor/miR-21a-5p Inhibitor-4.tif]

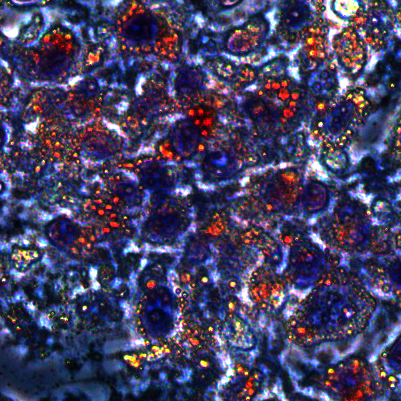

Supplement: Data S1. Raw experimental data generated in this study [file mmc1.zip › All original data/Morphological detection/Oil-red staining/Oil-red staining for Figure 5/miR-21a-5p Inhibitor/miR-21a-5p Inhibitor-5.tif]

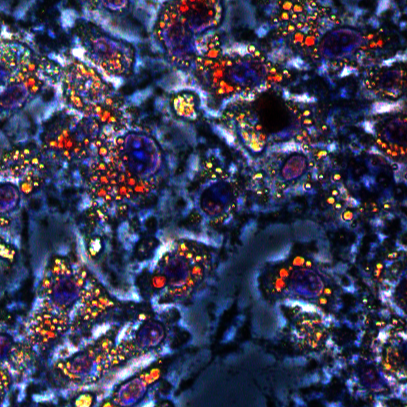

Supplement: Data S1. Raw experimental data generated in this study [file mmc1.zip › All original data/Morphological detection/Oil-red staining/Oil-red staining for Figure 5/miR-21a-5p Inhibitor/miR-21a-5p Inhibitor-6.tif]

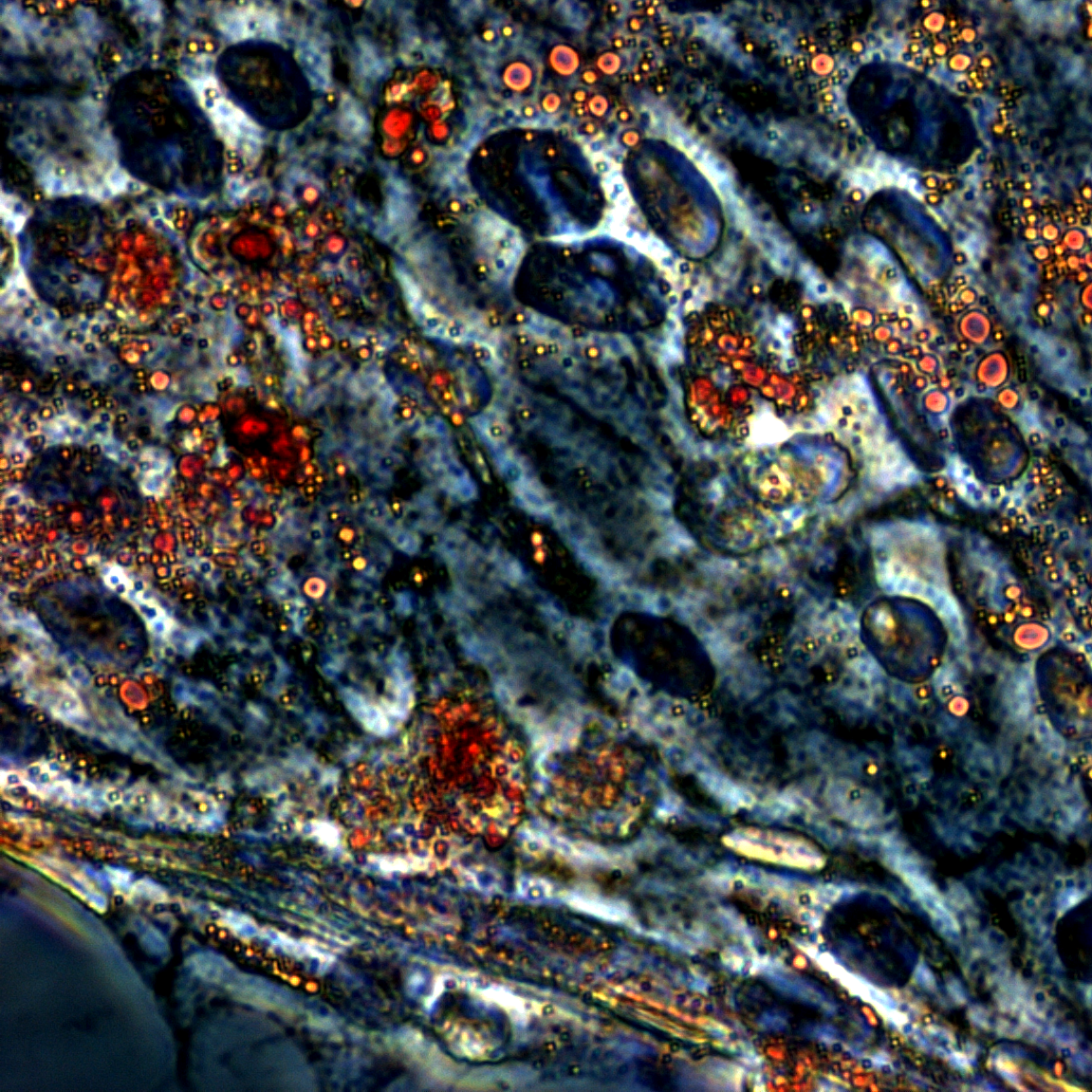

Supplement: Data S1. Raw experimental data generated in this study [file mmc1.zip › All original data/Morphological detection/Oil-red staining/Oil-red staining for Figure 5/miR-21a-5p Mimic/miR-21a-5p Mimic-1 used for manuscript.tif]

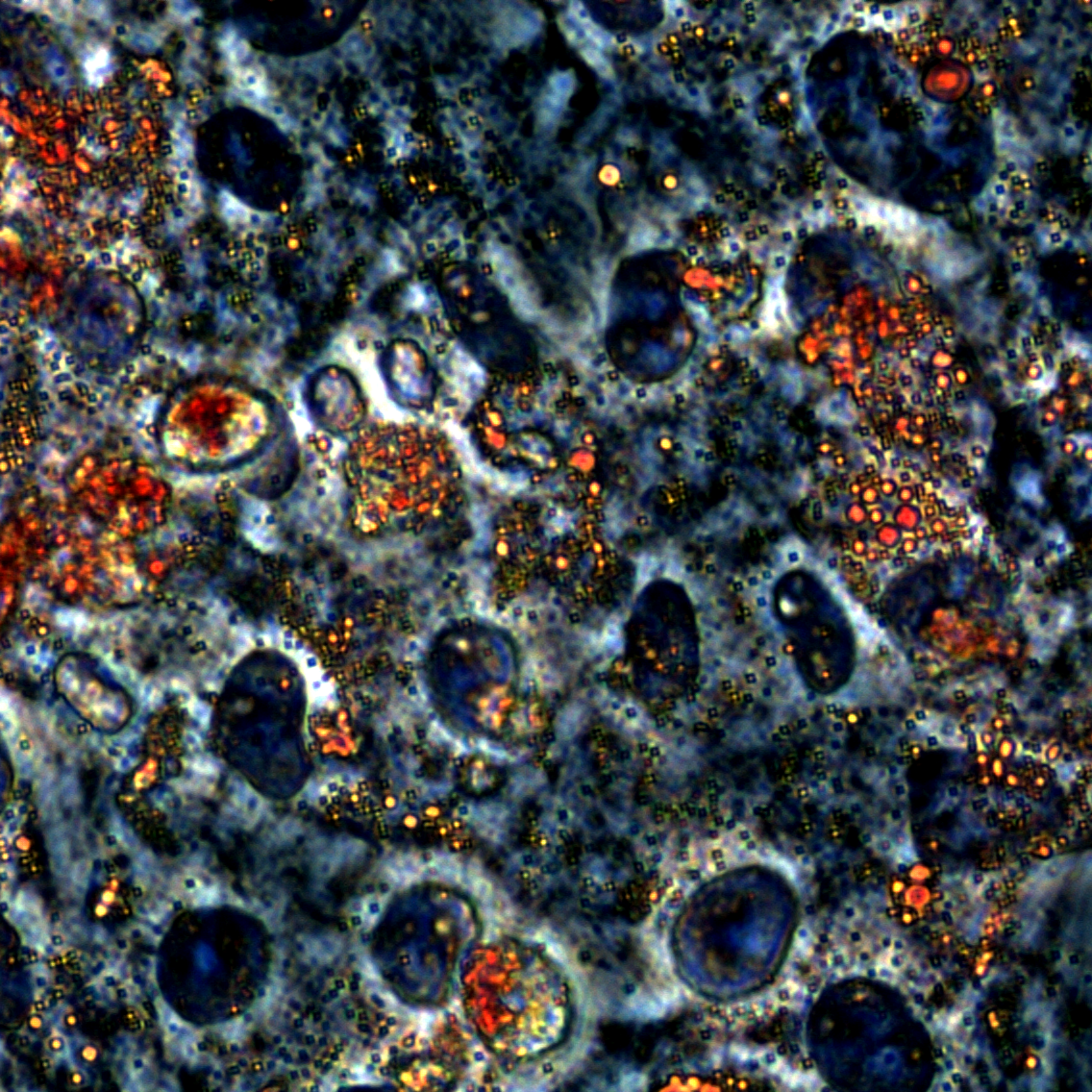

Supplement: Data S1. Raw experimental data generated in this study [file mmc1.zip › All original data/Morphological detection/Oil-red staining/Oil-red staining for Figure 5/miR-21a-5p Mimic/miR-21a-5p Mimic-2.tif]

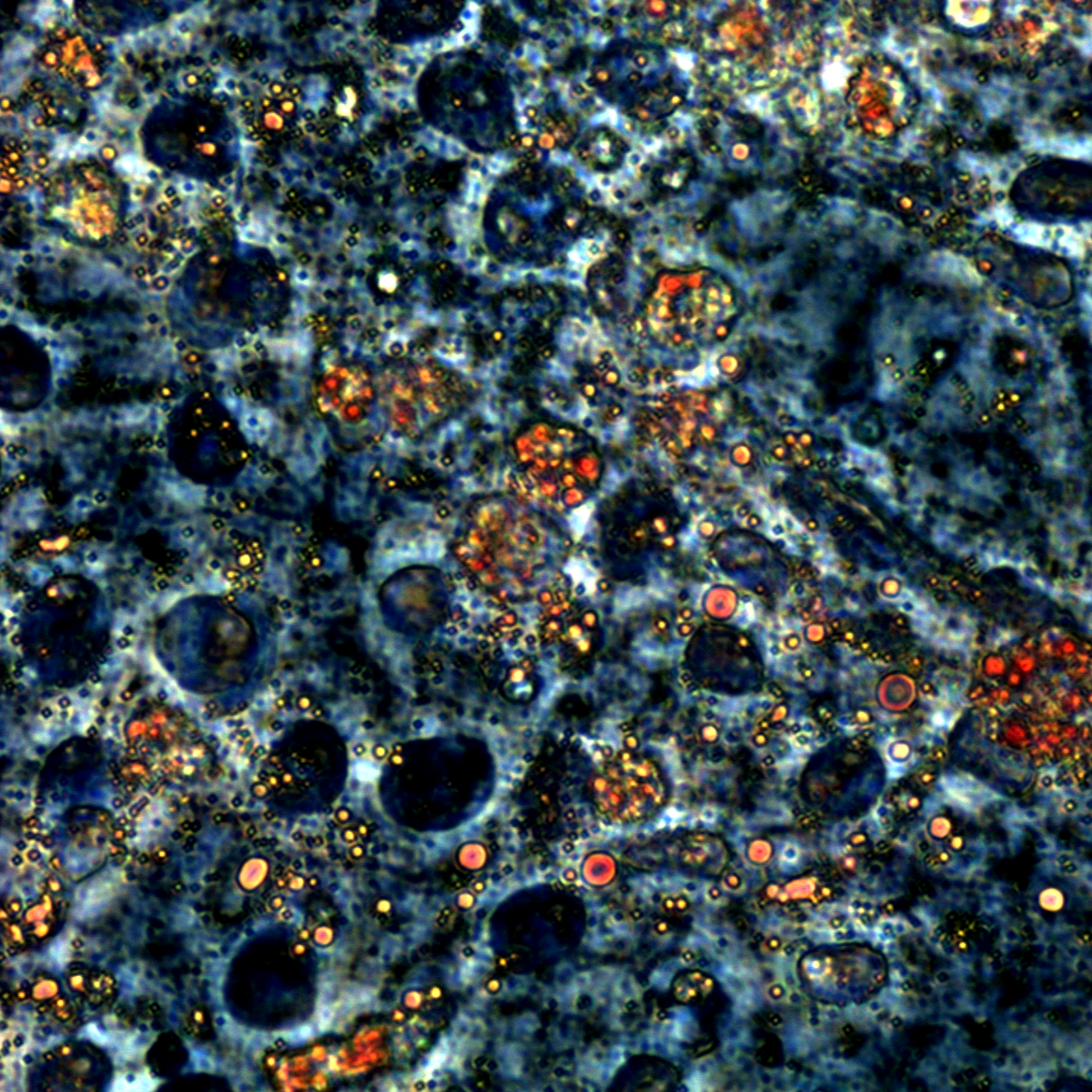

Supplement: Data S1. Raw experimental data generated in this study [file mmc1.zip › All original data/Morphological detection/Oil-red staining/Oil-red staining for Figure 5/miR-21a-5p Mimic/miR-21a-5p Mimic-3.tif]

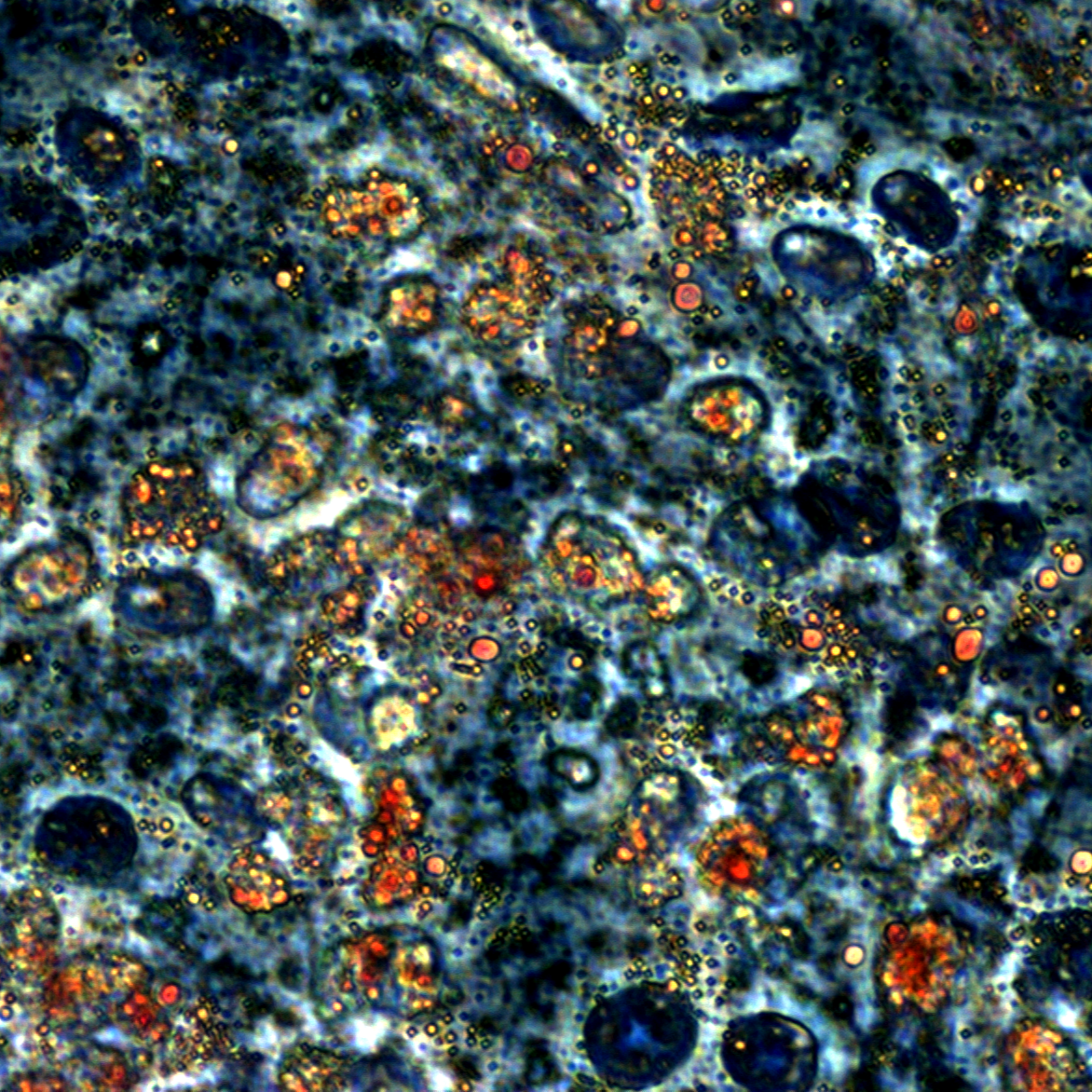

Supplement: Data S1. Raw experimental data generated in this study [file mmc1.zip › All original data/Morphological detection/Oil-red staining/Oil-red staining for Figure 5/miR-21a-5p Mimic/miR-21a-5p Mimic-4.tif]

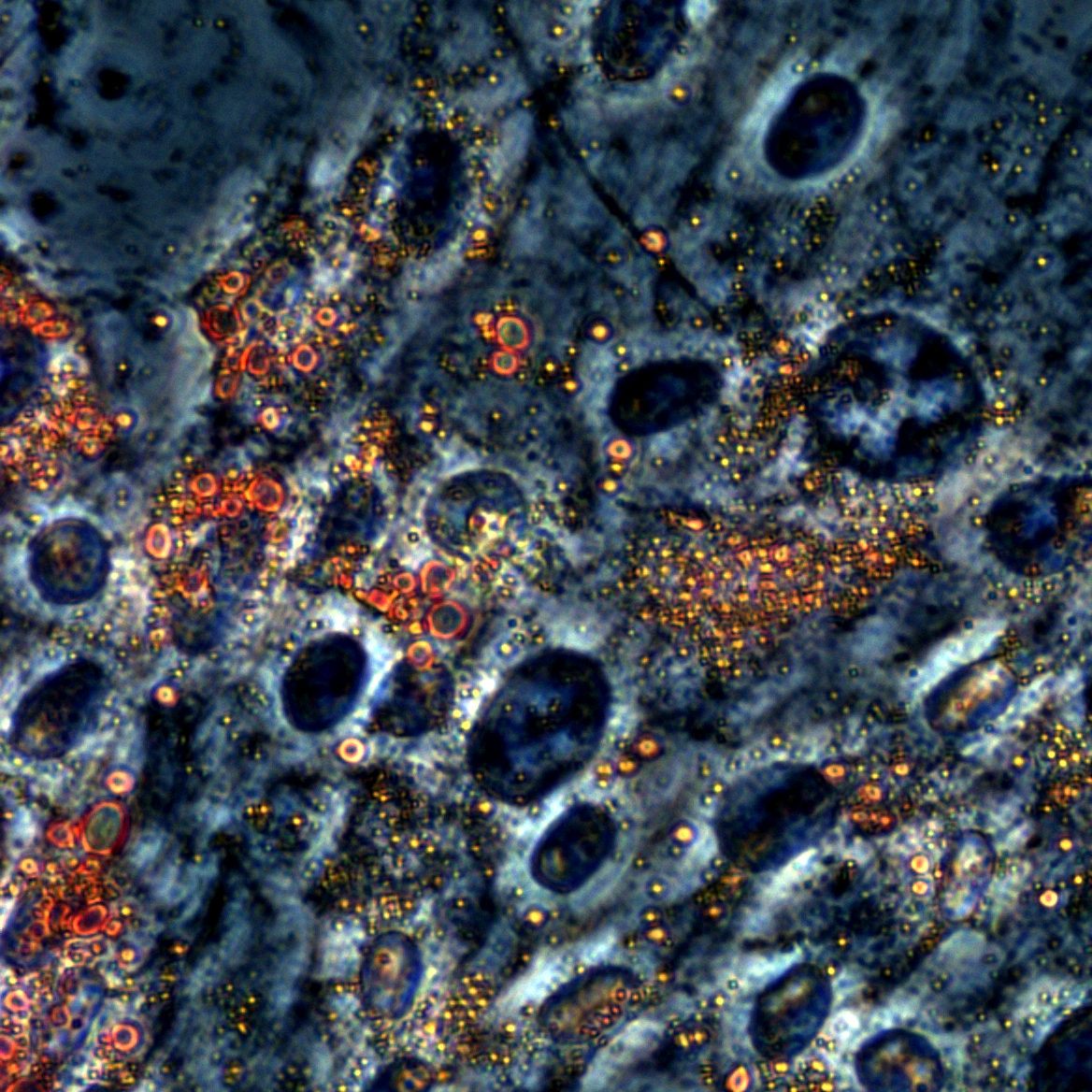

Supplement: Data S1. Raw experimental data generated in this study [file mmc1.zip › All original data/Morphological detection/Oil-red staining/Oil-red staining for Figure 5/miR-21a-5p Mimic/miR-21a-5p Mimic-5.tif]

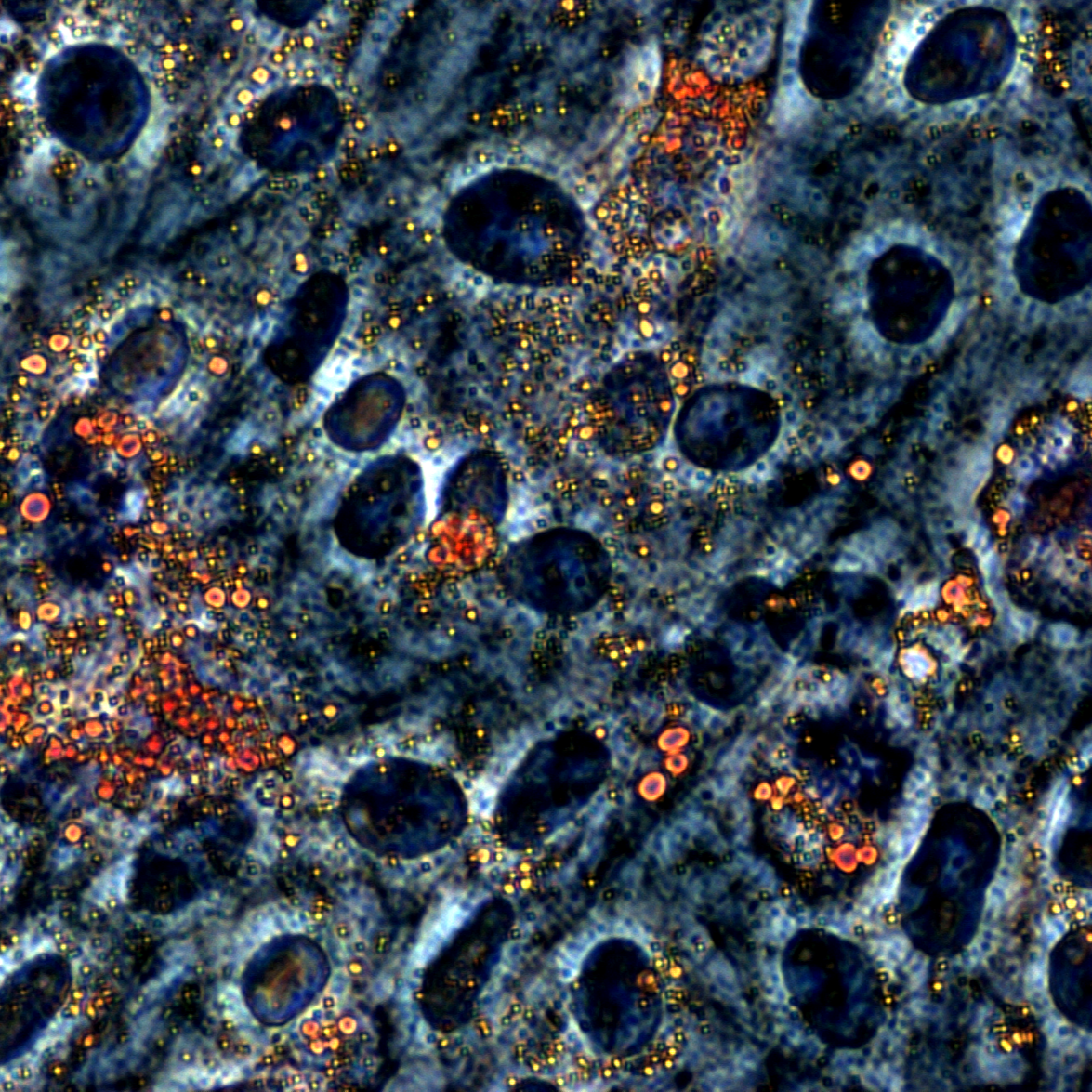

Supplement: Data S1. Raw experimental data generated in this study [file mmc1.zip › All original data/Morphological detection/Oil-red staining/Oil-red staining for Figure 5/miR-21a-5p Mimic/miR-21a-5p Mimic-6.tif]

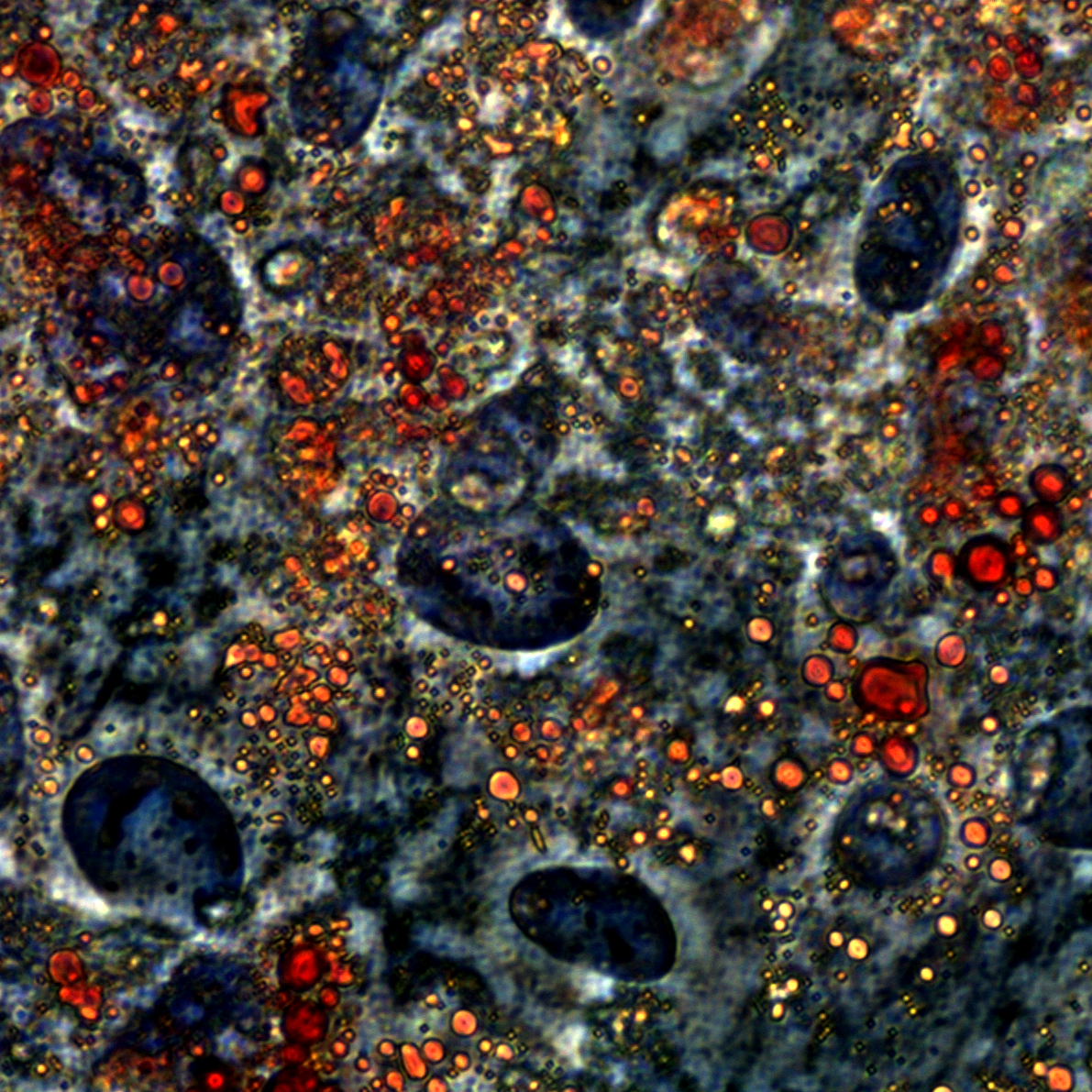

Supplement: Data S1. Raw experimental data generated in this study [file mmc1.zip › All original data/Morphological detection/Oil-red staining/Oil-red staining for Figure 5/NC/NC-1 used for manuscript.tif]

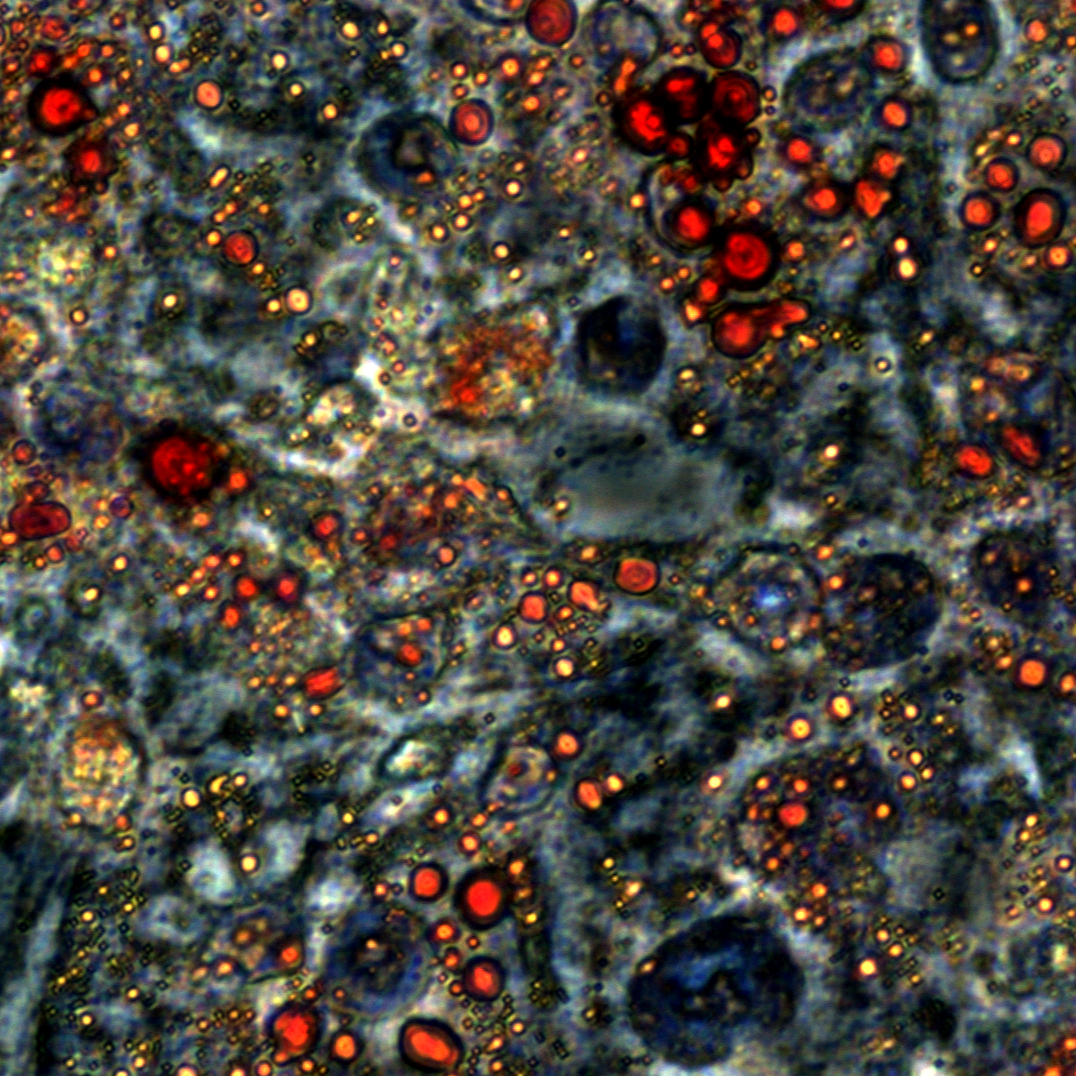

Supplement: Data S1. Raw experimental data generated in this study [file mmc1.zip › All original data/Morphological detection/Oil-red staining/Oil-red staining for Figure 5/NC/NC-2.tif]
