# Supplementary material for: A cell-free browning strategy: Exosomal miR-21a-5p from ADSCs targets PDCD4 to reshape adipose metabolism
Source: iScience. 2026 Jul 14;29(8):116765. doi: 10.1016/j.isci.2026.116765 (PMC13382807; doi:10.1016/j.isci.2026.116765)
Supplement: Data S1. Raw experimental data generated in this study [file WB original images.pdf › All original data/All data for New Figure 6 and New Figure 7/WB original images.pdf]

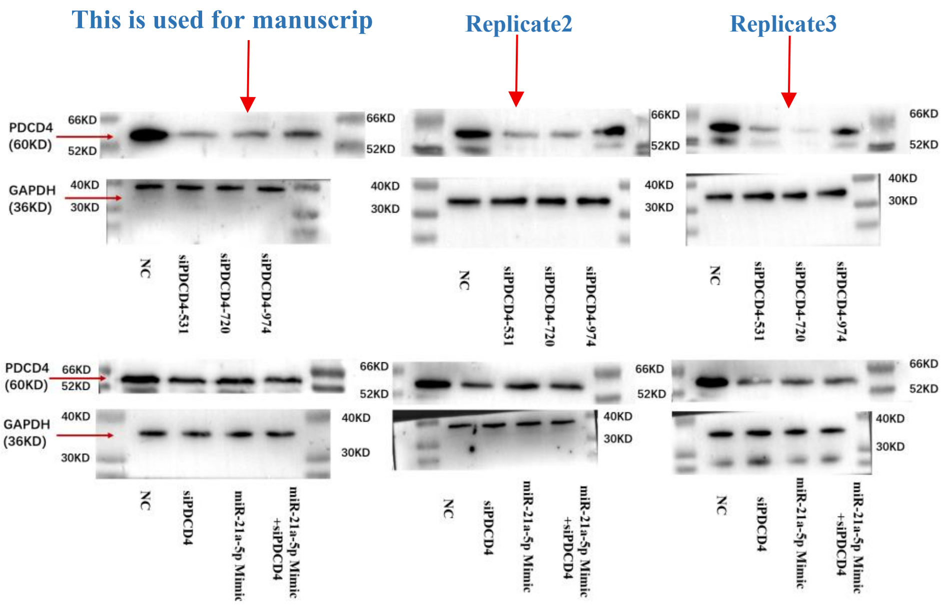

Original images for Western blotting for PDCD4/GAPDH in New Figure 6.

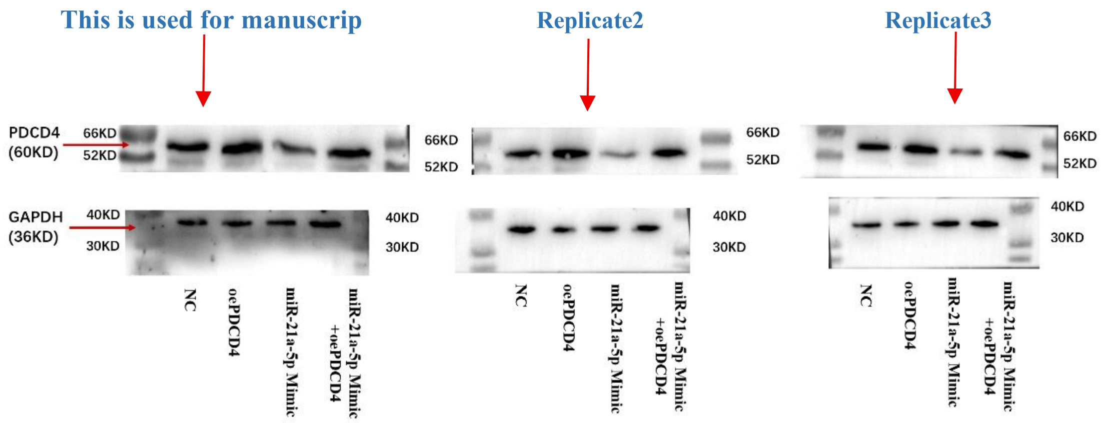

Original images for Western blotting for PDCD4/GAPDH in New Figure 7.
